# Isolation of Ten New Sesquiterpenes and New Abietane-Type Diterpenoid with Immunosuppressive Activity from Marine Fungus *Eutypella* sp.

**DOI:** 10.3390/ph18050737

**Published:** 2025-05-16

**Authors:** Nina Wang, Chunmei Chen, Qin Li, Qiqiang Liang, Yingjie Liu, Zongze Shao, Xiupian Liu, Qun Zhou

**Affiliations:** 1 Hubei Key Laboratory of Natural Medicinal Chemistry and Resource Evaluation, School of Pharmacy, Tongji Medical College, Huazhong University of Science and Technology, Wuhan 430030, China; 2 Key Laboratory of Marine Genetic Resources, Technology Innovation Center for Exploitation of Marine Biological Resources, Third Institute of Oceanography, Ministry of Natural Resources, Xiamen 361005, China

**Keywords:** *Eutypella* sp., immunosuppressive activity, secondary metabolites, sesquiterpenes

## Abstract

**Background**:**** Ten new sesquiterpenes, including eight eremophilane-type sesquiterpenes (**1**–**8**) and two compounds (**9**–**10**) with a cyclopentane ring, representing an undescribed subtype of sesquiterpene, along with a new abietane-type diterpenoid (**11**), were isolated and identified from a deep-sea-derived fungus: *Eutypella* sp. **Methods:** Their structures were elucidated on the basis of various spectroscopic analyses, mainly including nuclear magnetic resonance (NMR) and high-resolution electrospray ionization mass spectrometry (HRESIMS) data, ^13^C NMR calculations with DP4+ probability analyses, electronic circular dichroism (ECD) calculations, and single-crystal X-ray diffraction experiments. **Results:** Furthermore, compound **11** exhibited potent immunosuppressive activity with IC_50_ values of 8.99 ± 1.08 μM in a lipopolysaccharide (LPS) model and 5.39 ± 0.20 μM in a concanavalin A (ConA) model.

## 1. Introduction

**Figure 1:**
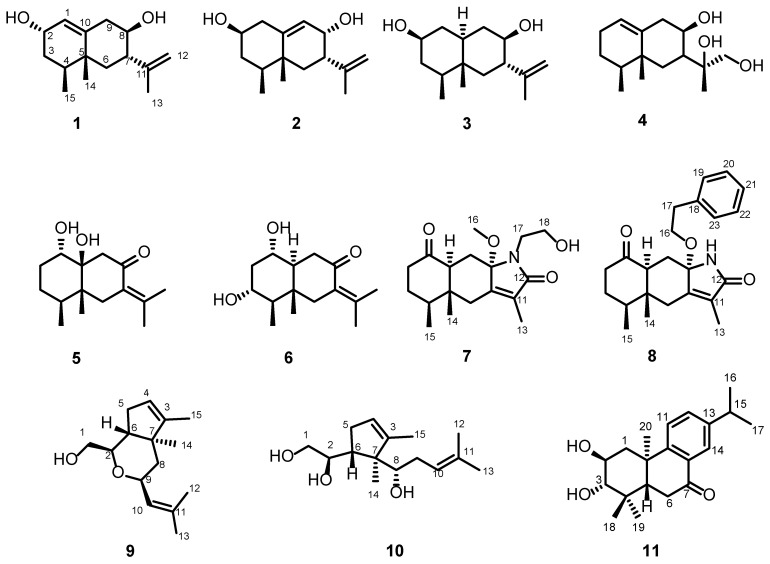
Structures of compounds **1**–**11**.

The dynamic environmental conditions of the ocean, characterized by significant fluctuations in pressure, salinity, temperature, pH, nutrient availability, and light intensity, exert selective pressures that drive marine microorganisms to evolve unique metabolic pathways for enhanced adaptation. This evolutionary process endows marine-derived secondary metabolites with remarkable bioactivities such as antitumor, antibacterial, and antiviral properties, while their distinct chemical scaffolds provide invaluable molecular resources for innovative drug discovery [1]. Marine fungus-derived bioactive compounds are potently applied for treatments of diseases [2,3], such as the antibiotics cephalosporin C, griseofulvin, and fusidic acid, used for the treatment of various pathogenic infections [4]. Research on fungi of the genus *Eutypella* over the past two decades has yielded diverse compounds, including terpenoids, cytochalasins, steroids, lactones, and diketopiperazines, which demonstrate notable cytotoxic, antibacterial, and anti-inflammatory activities [5,6,7,8,9,10]. The researchers isolated γ-lactones, sesquiterpenoids, and diterpenoids with moderate cytotoxic activity from the fermentation broth of *Eutypella* sp. BCC-13199, an endophyte of Zingiber species native to Thailand [6]. Epigenetic manipulation activated the sesquiterpene pathway in the fungus *Eutypella* sp., and twenty-six new eremophilane-type sesquiterpenoids were isolated, and two of their compounds exerted significant inhibition on NO production [11]. Jiao Binghua et al. isolated seven pimarane-type diterpenoid derivatives from *Eutypella* sp. D-1, a fungal strain obtained from high-latitude Arctic soils [8]. These compounds exhibited cytotoxic activity against human cancer cell lines and inhibited lipopolysaccharide (LPS)-induced NO production in lymphocytes. Terpenoids, with their unique chemical structures and broad spectrum of biological activities, demonstrate significant potential in the field of immunosuppression. Representative terpenoid immunosuppressive agents include triptolide [12], oridonin [13], and pseudopterosins [14]. With the aim of discovering bioactive natural products from deep sea organisms, we examined the metabolites of an unidentified fungus species, *Eutypella* sp. MCCC 3A00281, that was obtained from the deep sea sediment at a depth of 5610 m in the South Atlantic Ocean. Chromatographic separation of the EtOAc extract from the fermented fungus yielded a series of terpenes, including ten new sesquiterpenes (**1**–**10**) and one novel abietane-type diterpene (**11**) (Figure 1). Herein, we report their isolation and structural elucidation, and an evaluation of immunosuppressive activities.

## 2. Results and Discussion

### 2.1. Structural Elucidation

**Figure 2:**
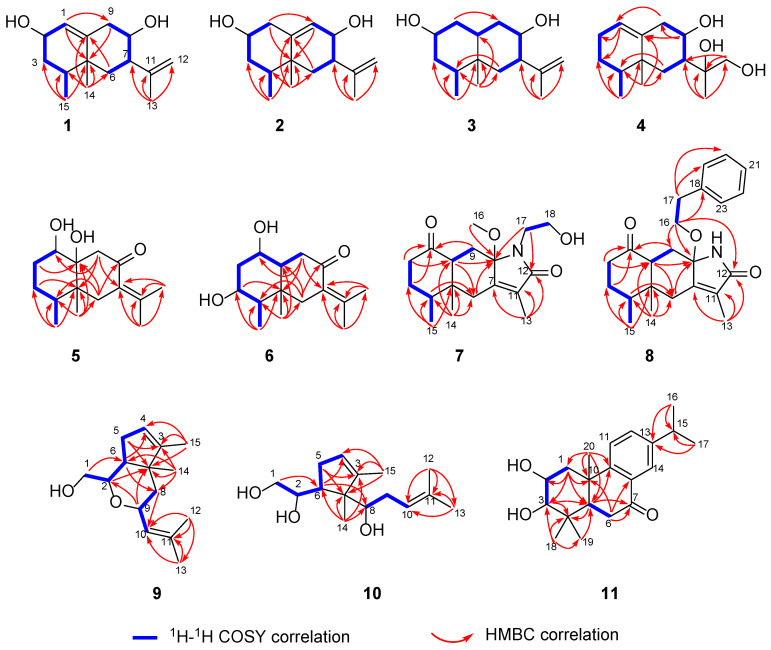
The key ^1^H−^1^H COSY and HMBCs of compounds **1**–**11**.

Compound **1** was isolated as a colorless oil. The molecular formula was established as C_15_H_24_O_2_ on the basis of an HREIMS peak at *m*/*z* 259.1663 [M + Na^+^] (calcd for C_15_H_24_O_2_Na^+^, 259.1674), consistent with 4 degrees of unsaturation. The ^1^H NMR data (Table 1) and heteronuclear single quantum coherence (HSQC) spectrum of compound **1** (Figure S4) reveal the following signals: three methyls (*δ*_H_ 1.65, 0.84, and 0.80), two terminal olefinic protons (*δ*_H_ 4.72), three methylenes (*δ*_H_ 1.35/1.49, 1.06/1.61, 2.21), two oxygenated methines (*δ*_H_ 3.84, 3.26), two methines (*δ*_H_ 1.64, 2.18), one olefinic methine (*δ*_H_ 5.38), and two exchangeable proton signals (*δ*_H_ 4.43, 4.45). The ^13^C NMR data (Table 2) and DEPT-135 spectrum (Figure S3) confirm the presence of 15 carbons, including the following: three methyls (*δ*_C_ 15.5, 16.5, 19.5), three methylenes (*δ*_C_ 36.2, 41.6, 42.7), one olefinic methylene (*δ*_C_ 111.2), two oxygenated methines (*δ*_C_ 62.3, 71.2), one olefinic methine (*δ*_C_ 124.2), two methines (*δ*_C_ 33.9, 48.5), one quaternary carbon (*δ*_C_ 37.2), and two olefinic quaternary carbons (*δ*_C_ 143.9, 147.4). The ^13^C NMR data of compound **1** closely align with those of the known eremophilane-type sesquiterpene 7*β*H-eremophil-1(10),11(12)-dien-2*β*,8*β*-diol [15]. However, distinct differences are observed in the chemical shifts and coupling constants of H-1, H-2, and H_2_-3 and their corresponding carbons (C-1, C-2, C-3), suggesting potential structural modifications in these regions. The planar structure of Compound **1** was determined to be identical to that of the known compound based on heteronuclear multiple bond correlation (HMBC) and ^1^H-^1^H correlation spectroscopy (^1^H-^1^H COSY) correlations (Figure 2).

**Figure 3:**
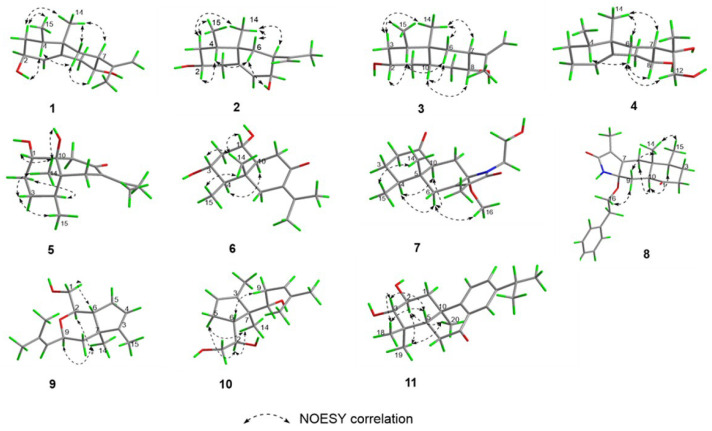
The key NOESY correlations of compounds **1**–**11**.

**Figure 4:**
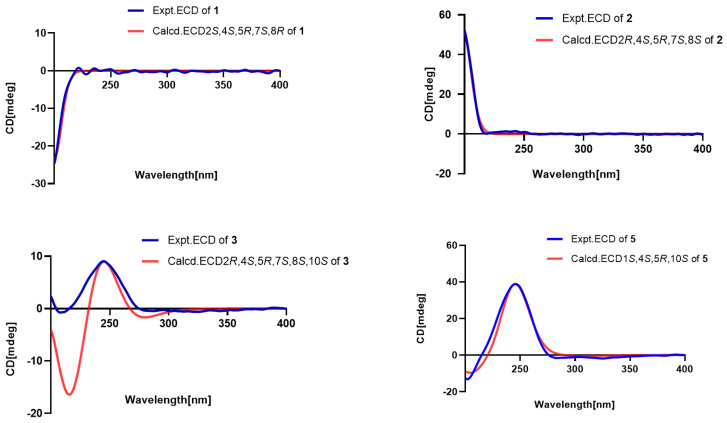
Experimental and calculated ECD spectra of compounds **1**–**3**, **5**.

The relative configurations were established on the basis of nuclear Overhauser effect spectroscopy (NOESY) cross peaks. As a general tendency, biosynthesis results in eremophilane structures with CH_3_-14 in *β*-orientation; consequently, this position was assigned accordingly. The key NOESY cross-peaks (Figure 3) between H-3ax (*δ*_H_ 1.49)/CH_3_-15 (*δ*_H_ 0.80)/CH_3_-14 (*δ*_H_ 0.84), CH_3_-14/H-6eq (*δ*_H_ 1.61)/H-7 (*δ*_H_ 2.18), OH-2 (*δ*_H_ 4.43)/H-4 (*δ*_H_ 1.64), H-4/H-6ax (*δ*_H_ 1.06), and H-6ax/H-8 (*δ*_H_ 3.26) collectively support the β-orientations of CH_3_-14, CH_3_-15, and H-7, while H-2, H-4, and H-8 adopt α-orientations. Therefore, the relative configuration of compound **1** was deduced as 2S*,4S*,5R*,7S*,8R*. Finally, the absolute configuration of compound **1** was assigned as 2*S*,4*S*,5*R*,7*S*,8*R* by comparison of its experimental and computed ECD curves (Figure 4).

Compound **2** was isolated as a colorless oil. The molecular formula was established as C_15_H_24_O_2_ on the basis of a HREIMS peak at *m*/*z* 259.1668 [M + Na^+^] (calcd for C_15_H_24_O_2_Na^+^, 259.1674). The HMBC and ^1^H-^1^H COSY correlations revealed that the double bond position in compound **2** was altered compared to compound **1**, shifting from C-1–C-10 to C-9–C-10 (Figure 2). The key NOESY cross-peaks of CH_3_-15 (*δ*_H_ 0.80)/CH_3_-14 (*δ*_H_ 0.84) and H-3ax (*δ*_H_ 1.49), CH_3_-14 and H-7 (*δ*_H_ 2.18), and OH-2 (*δ*_H_ 4.43)/H-4 (*δ*_H_ 1.64)/H-6ax (*δ*_H_ 1.06)/H-8 (*δ*_H_ 3.26) suggested that H-7, CH_3_-14, and CH_3_-15 were on the same side with *β*-orientations, while H-2, H-4, and H-8 was on the opposite side (*α*-orientation). The absolute configuration of compound 2 (2*R*,4*S*,5*R*,7*S*,8*S*) was determined by comparative analysis of ECD calculations and experimental data (Figure 4).

Compound **3** was determined to have the molecular formula C_15_H_26_O_2_, as deduced from the HRESIMS data (*m*/*z* 261.1826 [M + Na^+^], calcd for C_15_H_26_O_2_Na^+^, 261.1830). The ^1^H NMR data (Table 1) displayed three methyls (*δ*_H_ 1.63, 0.72, and 0.68), five methylenes (*δ*_H_ 4.67, 1.52/1.13, 1.44/0.91, 1.45/1.24, and 1.40/1.04), five methines (*δ*_H_ 3.39, 3.29, 2.05, 1.10, and 1.09), and two hydroxyls (*δ*_H_ 4.43 and 4.19). The ^13^C NMR spectrum (Table 2) showed 15 carbon signals attributed to three methyls (*δ*_C_ 19.6, 15.3, and 11.2), five methylenes (*δ*_C_ 110.9, 42.9, 40.1, 40.0, and 37.8), five methines (*δ*_C_ 70.9, 68.7, 48.6, 41.8, and 40.6), and two non-protonated carbons (*δ*_C_ 148.0 and 35.4). The ^1^D-NMR data of **3** combined with the sequential ^1^H-^1^H COSY correlations of Me-15/H-4/CH_2_-3/H-2/CH_2_-1/H-10/CH_2_-9/H-8/H-7/CH_2_-6, as well as the key HMBC from Me-15 to C-3/C-4/C-5, Me-14 to C-4/C-5/C-6/C-10, C-6 to C-4, and Me-13 to C-12/C-7/C-11 suggested an eremophilane sesquiterpene with a 6/6-fused ring system skeleton.

Key NOESY correlations (Figure 3) revealed spatial proximities: CH_3_-14 (*δ*_H_ 0.68) to H-7 (*δ*_H_ 2.05) and H-3ax (*δ*_H_ 1.13), and Me-15 (*δ*_H_ 0.72) to H-3ax, supporting β-orientations for CH_3_-14, CH_3_-15, and H-7; H-2 (*δ*_H_ 3.39)/H-4 (*δ*_H_ 1.09)/H-10 (*δ*_H_ 1.10)/H-8 (*δ*_H_ 3.29)/H-6ax (*δ*_H_ 0.91), indicating α-orientations for these protons. The absolute configuration of compound 3 (2*R*,4*S*,5*R*,7*S*,8*S*,10*S*) was determined by comparative analysis of ECD calculations and experimental data.

**Figure 5:**
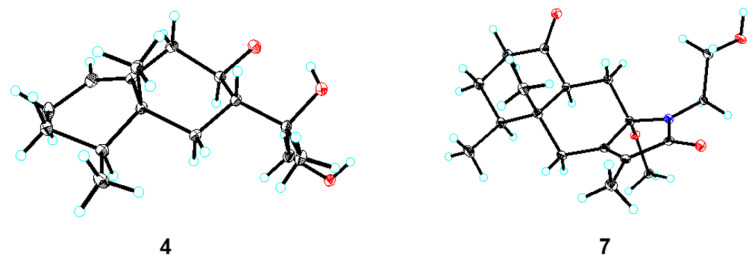
X-ray crystal structures of compounds **4** and **7**. Black represents C atoms, blue represents H atoms, red represents O atoms.

Compound **4** gave a molecular formula of C_15_H_26_O_3_, as evidenced via its HRESIMS data. The ^1^H and ^13^C NMR signals (Table 1 and Table 2), along with the ^1^H−^1^H COSY and HMBC spectrum, showed that the planar structure of compound **4** is similar to valenc-1(10)-ene-8,11-diol [16]. Unlike known compounds, the hydroxymethyl group was assigned to C-12 based on the HMBC long-range correlations between the proton signal at *δ*_H_ 3.75 (H-12) and C-7, C-11, and C-13. The NOESY correlations (Figure 3) from CH_3_-14 (*δ*_H_ 0.95) to H-6*β* (*δ*_H_ 1.83)/H-7 (*δ*_H_ 1.90), and cross-peaks of H-4 (*δ*_H_ 1.33)/H-6ax (*δ*_H_ 0.78)/CH_3_-12 (*δ*_H_ 3.75) determined that CH_3_-14, CH_3_-15, and H-7 were on the same side with *β*-orientations. Considering the biosynthetic pathway and single-crystal X-ray diffraction experiment, the absolute configuration of compound **4** was confirmed as 4*S*,5*R*,7*R*,8*R*,11*S* by the single-crystal X-ray diffraction analysis using Cu Kα radiation (Figure 5) [Flack parameter = 0.03(12), CCDC 2431789].

Compound **5** was isolated as a colorless oil and provided a molecular formula of C_15_H_24_O_3_, according to its HRESIMS data (*m*/*z* 275.1631 [M + Na^+^]), which suggested four degrees of unsaturation. The ^1^H NMR data (Table 1) displayed four methyls (*δ*_H_ 1.81, 1.75, 0.83, and 0.77), four methylenes (*δ*_H_ 2.66/2.31, 2.44/2.37, 1.63/1.33, and 1.43/1.30), two methines (*δ*_H_ 3.53 and 1.69), and two hydroxyls (*δ*_H_ 4.45 and 4.35). The ^13^C NMR spectrum (Table 2) of compound **5** showed 15 carbon signals attributed to four methyls (*δ*_C_ 22.2, 21.2, 16.0, and 14.2), four methylenes (*δ*_C_ 45.0, 35.9, 30.9, and 28.4), two methines (*δ*_C_ 71.3 and 32.4), and five non-protonated carbons (*δ*_C_ 203.4, 137.7, 131.1, 78.3, and 42.2). The planar structure of compound **5** was inferred to be identical to eutyperemophilane R by ^1^H-^1^H COSY and HMBC data (Figure 2) [11]. The difference between compound **5** and eutyperemophilane R was that C-10 was hydroxylated in compound **5**.

The NOE correlations of Me-14 (*δ*_H_ 0.83) with OH-10 (*δ*_H_ 4.45)/ H-1 (*δ*_H_ 3.53), H-3ax (*δ*_H_ 1.30) with Me-14 (*δ*_H_ 0.83)/ Me-15 (*δ*_H_ 0.77), and H-4 (*δ*_H_ 1.69) with H-2ax (*δ*_H_ 1.32) revealed relative configuration. The absolute configuration of compound **5** (1*S*,4*S*,5*R*,10*S*) was determined by comparative analysis of ECD calculations and experimental data (Figure 4).

Compound **6** gave a molecular formula of C_15_H_24_O_3_, as evidenced via its HRESIMS data. Although compound **6** shares the same molecular formula as compound **5**, its planar structure was deduced to have a 1,3-dihydroxy substitution based on ^1^H-^1^H COSY and HMBCs (Figure 2). Analysis of the NOESY correlations (Figure 3), the spatial proximities between CH_3_-14 and H-1, H-3, and CH_3_-15 indicate *β*-configurations for H-1, H-3, and CH_3_-15. The correlation between H-10 and H-4 suggests an *α*-configuration for H-10. The relative configuration of compound **6** was determined as 1S*,3R*,4R*,5R*,10S*, while absolute configuration was confirmed by comparative analysis of ECD calculations and experimental data.

Compound **7** was isolated as a colorless needle crystal. The molecular formula was established as C_18_H_27_NO_4_ on the basis of a HREIMS peak at *m*/*z* 344.1835 [M + Na^+^] (calcd for C_18_H_27_NO_4_Na^+^, 344.1838). The ^1^H and ^13^C NMR data (Table 3 and Table 4) of compound **7** were very similar to those of the known compound eremophilane lactam, which indicated that both compounds shared the same core skeleton [11]. The difference between compound **7** and eremophilane lactam was the substitution of the N-H hydrogen with a hydroxyethyl group, as supported by the key HMBCs from H_2_-17 to C-8 and C-12. Considering the NOE correlations and single-crystal X-ray diffraction experiment (Figure 5), the absolute configuration of compound **7** was confirmed as 4*S*,5*R*, 8*S*,10*S* by the single-crystal X-ray diffraction analysis using Cu Kα radiation [Flack parameter = 0.01(6), CCDC 2431799].

Compound **8** possessed a molecular formula of C_23_H_29_NO_3_, as determined by the HRESIMS and NMR data. Comprehensive analysis of the NMR data of compound **7** and compound **8** suggested that both compounds were structural analogs; the difference lies in the substitution of a methyl group in compound **7** with a phenethyl group in compound **8**, accompanied by *N*-reduction. This structural modification was corroborated by HMBCs between H_2_-16 and C-18, as well as between H_2_-17 and C-18, C-19, and C-20 (Figure 2).

By analysis of the NOESY data, Me-14 (*δ*_H_ 0.47) showed strong NOESY correlations to Me-15 (*δ*_H_ 1.00) and H-9ax (*δ*_H_ 1.35), indicating that Me-15, Me-14, and H-9ax were on the same face with *β*-orientations, while cross-peaks of H-4 (*δ*_H_ 2.02)/H-10 (*δ*_H_ 2.73) suggested that H-10 was on the opposite side (*α*-orientation). The correlation of H-16 (*δ*_H_ 3.45)/H-9eq (*δ*_H_ 2.26) suggested that hyphenated oxygen groups were *α*-oriented. The absolute configuration was determined by a comparative analysis of ECD calculations and experimental data.

Compound **9** was isolated as a colorless oil. The molecular formula was established as C_15_H_24_O_2_ on the basis of a HREIMS peak at *m*/*z* 259.1668 [M + Na^+^] (calcd for C_15_H_24_O_2_Na^+^, 259.1674), bearing four degrees of unsaturation. The ^13^C NMR spectrum exhibited 15 carbon resonances, including four olefinic carbons for two double bonds, a sp^3^ quaternary carbon, three methylenes, three methines, and four methyl groups (Table 3 and Table 4). The COSY correlations of H-5 (*δ*_H_ 1.92, 2.04) with H-4 (*δ*_H_ 5.28) and H-6 (*δ*_H_ 1.71), together with the HMBCs from CH_3_-15 (*δ*_H_ 1.65) to C-3 (*δ*_C_ 150.9), C-4 (*δ*_C_ 122.5), and C-7 (*δ*_C_ 45.6), and from CH_3_-14 (*δ*_H_ 0.95) to C-3, C-6 (*δ*_C_ 50.7), and C-7, established a dimethylcyclopentene unit. The COSY correlations from H-6 to H-2 (*δ*_H_ 3.81) and CH_2_-1 (*δ*_H_ 3.68), and from H-9 (*δ*_H_ 4.54) to CH_2_-8 (*δ*_H_ 1.32, 1.54) and H-10 (*δ*_H_ 5.19), in association with the HMBC from CH_2_-1 to C-2 (*δ*_C_ 75.8) and C-6, and from H-9 to C-7 and C-2, fused a pyran ring to the cyclopentene nucleus. In addition, a 2-methylpropenyl side chain was deduced based on the HMBCs of CH_3_-12 (*δ*_H_ 1.73)/CH_3_-13 (*δ*_H_ 1.69) with C-10 (*δ*_C_ 126.6) and C-11 (*δ*_C_ 135.3). This side chain was connected to C-9 via the HMBCs between CH_2_-8 and C-10 (Figure 2).

**Figure 6:**
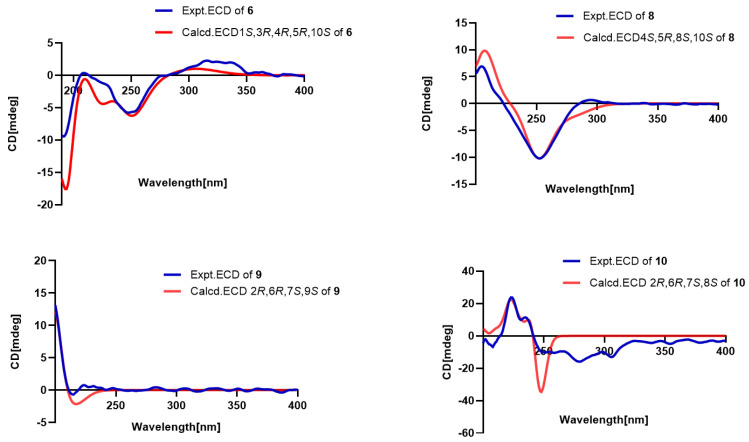
Experimental and calculated ECD spectra of compounds **6**, **8**–**10**.

The NOE correlations from CH_3_-14 and H-2/H-9, as well as between H-1 and H-6, indicate that H-2, CH_3_-14, and H-9 are positioned on the opposite face relative to H-6 (Figure 3). The experimental ECD data of compound 9 were similar to the calculated data for (2*R*,6*R*,7*S*,9*S*)-9 (Figure 6), determining the absolute configuration.

Comparative analysis of 1D NMR data combined with COSY and HMBCs revealed that compound **10** shares identical partial structures (cyclopentene unit and side chain) with compound **9**. The key structural divergence lies in the formation of a pyran ring in compound **9** via dehydration–condensation between OH-9 and OH-2, whereas in compound **10**, C-8 is oxidized to a hydroxyl group without oxygen ring formation.

**Figure 7:**
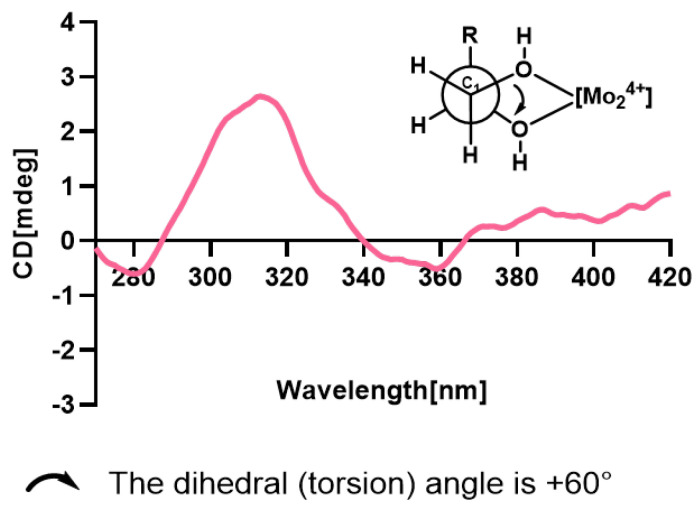
ECD spectra of the Mo_2_(AcO)_4_ complex of compound **10**.

**Figure 8:**
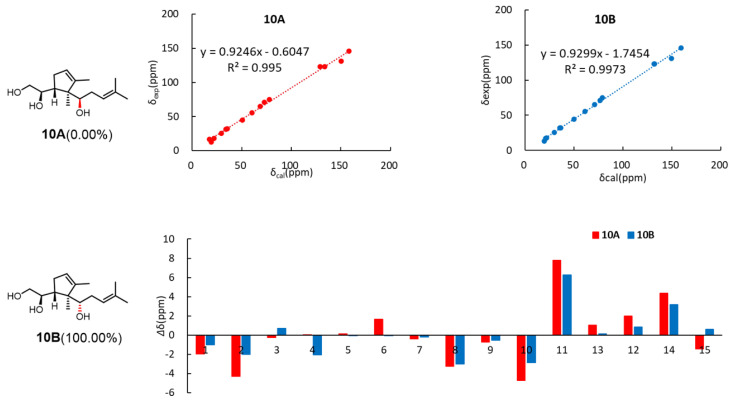
^13^ C NMR calculation results of two possible isomers of compound **10**.

In the NOESY spectrum, H-6 showed correlations with H-9, and CH_3_-14 exhibited mutual correlations with H-2 and H-5ax. A weak correlation between H-6 and H-5ax, contrasted with a strong correlation between H-6 and H-5eq, indicated that CH_3_-14, H-5ax, and H-2 are positioned on the opposite face of the ring relative to H-6. The relative configuration was deduced as 2R*,6R*,7S*. Based on Snatzke’s method [17], the treatment of **10** with Mo_2_(OAc)_4_ derived an Mo_2_(OAc)_4_ complex, whose ECD data showed a positive cotton effect at 311 nm and 400 nm in DMSO (Figure 7), reflecting the 2*R* configuration. To identify the final structure for compound **10**, the theoretical ^13^C NMR calculation with DP4+ probability analyses were employed. The ^13^C NMR chemical shifts in two possible isomers, **10A** (2*R*,6*R*,7*S*,8R*) and **10B** (2*R*,6*R*,7*S*,8S*), were calculated at the B3LYP-D3(BJ)/6-31G* level, and the calculated results for **10B** (R^2^ = 0.9973) showed a better match to the experimental data than **10A** (R^2^ = 0.995). Furthermore, according to the DP4+ probability analyses, **10B** was assigned with a 100% probability (Figure 8). The above messages demonstrated **10B** to be the right relative structure for compound **10**. The absolute configuration of compound **10** was finally determined as 2*R*,6*R*,7*S*,8*S* by comparison of the experimental and computed ECD curves (Figure 6).

**Figure 9:**
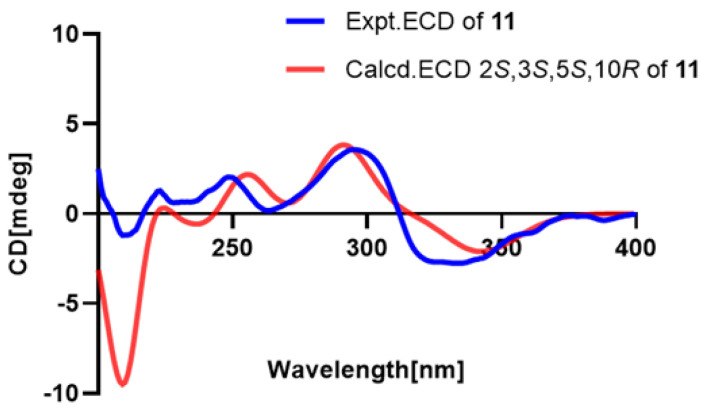
Experimental and calculated ECD spectra of compound **11**.

The molecular formula of compound **11** was established as C_20_H_28_O_3_ by the HRESIMS data. The ^1^H and ^13^C NMR data of compound **11** were comparable to the known compound hydroxyldecandrin G [18], but exhibited an additional proton signal *δ*_H_ 2.84 and distinct chemical shift differences in the side chain (C-11–C-17): C-11 (Δ*δ*_C_ +0.6), C-12 (Δ*δ*_C_ +1.7), C-13 (Δ*δ*_C_ +1.7), C-14 (Δ*δ*_C_ +1.1), C-15 (Δ*δ*_C_ −37.8), and C-16/17 (Δ*δ*_C_ −7.5) (Table 5). These discrepancies could only be rationalized by proposing the reduction of the OH-15 group. This hypothesis was further corroborated by ^1^H–^1^H COSY and HMBC analyses (Figure 2), which confirmed the planar structure. The NOESY correlations from CH_3_-18 to H-5 and H-3 and from H-2 to CH_3_-19 and CH_3_-20, along with the coupling constant between H-2 and H-3 (*J* = 9.7 Hz), revealed the relative configuration of compound **11**. The absolute configuration of compound **11** (2*S*,3*S*,5*S*,10*R*) was determined by comparing its 1D NMR data with the known compound hydroxyldecandrin G, as well as by analyzing the agreement between the calculated and experimentally measured ECD spectra (Figure 9).

### 2.2. Immunosuppressive Activity

In the concanavalin A (ConA)-induced lymphocyte proliferation assay, compounds **1** and **9** exhibited moderate inhibitory activity with half-maximal inhibitory concentration (IC_50_) values of 13.55 ± 1.40 μM and 29.25 ± 1.56 μM, respectively. Notably, compound **11** demonstrated more potent suppression against both lipopolysaccharide (LPS)- and ConA-induced lymphocyte proliferation, showing IC_50_ values of 8.99 ± 1.08 μM (LPS model) and 5.39 ± 0.20 μM (ConA model). Compound **11** exhibited potent activity, presumably attributed to its diterpenoid nature, while the enhanced activity of compound **1** among sesquiterpenoid analogs may be due to the presence of a double bond between C-1 and C-10.

### 2.3. Spectroscopic Data of Compounds

#### 2.3.1. Compound **1**

Colorless oil; ${\left[\alpha \right]}_{D}^{24}$ −191 (*c* 0.1, MeOH), UV (MeCN) *λ*_max_ (log *ε*) = 194 (4.16), 245 (2.50), 279 (2.86), 335(0.23) nm; ECD (MeCN) λ_max_ (Δ*ε*) = 199 (−7.87), 222 (+0.26) nm; for ^1^H and ^13^C NMR data, see Table 1 and Table 2; HRESIMS *m*/*z* 259.1663 [M + Na^+^] (calcd. for C_15_H_24_O_2_Na^+^, 259.1674).

#### 2.3.2. Compound **2**

Colorless oil; ${\left[\alpha \right]}_{D}^{24}$ +238 (*c* 0.1, MeOH), UV (MeCN) *λ*_max_ (log *ε*) = 195 (4.02), 226 (2.27) nm; ECD (MeCN) λ_max_ (Δ*ε*) = 198 (+15.25), 218 (+0.01) nm; IR (KBr) *ν*_max_ 3360, 2964, 2931, 2856, 1685, 1461, 1443, 1034, 1019 and 888cm^−1^ cm^−1^; for ^1^H and ^13^C NMR data, see Table 1 and Table 2; HRESIMS *m*/*z* 259.1668 [M + Na^+^] (calcd. for C_15_H_24_O_2_Na^+^, 259.1674).

#### 2.3.3. Compound **3**

Colorless oil; ${\left[\alpha \right]}_{D}^{24}$ −61 (*c* 0.1, MeOH), UV (MeCN) *λ*_max_ (log *ε*) = 200 (4.20), 220 (2.93) nm; ECD (MeCN) λ_max_ (Δ*ε*) = 209 (−0.13), 245 (+1.64), 258 (−0.08) nm; IR (KBr) *ν*_max_ 3325, 2969, 2926, 2857, 1648, 1466, 1444, 1032 and 883 cm^−1^; for ^1^H and ^13^C NMR data, see Table 1 and Table 2; HRESIMS *m*/*z* 261.1826 [M + Na^+^] (calcd. for C_15_H_26_O_2_Na^+^, 261.1830).

#### 2.3.4. Compound **4**

Colorless needle crystals; ${\left[\alpha \right]}_{D}^{24}$ −391 (*c* 0.1, MeOH), UV (MeCN) *λ*_max_ (log *ε*) = 192 (4.48), 373 (1.00) nm; ECD (MeCN) λ_max_ (Δ*ε*) = 204 (−0.70) nm; IR (KBr) *ν*_max_ 3379, 2963, 2923, 2855, 1663, 1459, 1381 and 1039 cm^−1^; for ^1^H and ^13^C NMR data, see Table 1 and Table 2; HRESIMS *m*/*z* 277.1771 [M + Na^+^] (calcd. for C_15_H_26_O_3_Na^+^, 277.1780).

#### 2.3.5. Compound **5**

Colorless oil; ${\left[\alpha \right]}_{D}^{24}$ −339 (*c* 0.1, MeOH), UV (MeCN) *λ*_max_ (log *ε*) = 200 (3.74), 215 (3.32), 249 (3.88), 383 (1.05) nm; ECD (MeCN) λ_max_ (Δ*ε*) = 202 (−3.98), 246 (+11.87) nm; for ^1^H and ^13^C NMR data, see Table 1 and Table 2; HRESIMS *m*/*z* 275.1631 [M + Na^+^] (calcd. for C_15_H_24_O_3_Na^+^, 275.1623).

#### 2.3.6. Compound **6**

White amorphous powders; ${\left[\alpha \right]}_{D}^{24}$ +126 (*c* 0.1, MeOH), UV (MeCN) *λ*_max_ (log *ε*) = 191 (3.89), 215 (3.36), 249 (3.84), 393 (1.46) nm; ECD (MeCN) λ_max_ (Δ*ε*) = 192 (−2.48), 209 (+0.15), 226 (−0.37), 228 (−0.31), 245 (−1.52) nm; IR (KBr) *ν*_max_ 3393, 2968, 2923, 2854, 1671, 1453 and 1043 cm^−1^; for ^1^H and ^13^C NMR data, see Table 3 and Table 4; HRESIMS *m*/*z* 275.1626 [M + Na^+^] (calcd. for C_15_H_24_O_3_Na^+^, 275.1623).

#### 2.3.7. Compound **7**

Colorless needle crystals; ${\left[\alpha \right]}_{D}^{24}$ −286 (*c* 0.1, MeOH), UV (MeCN) *λ*_max_ (log *ε*) = 214 (3.85), 375 (−0.54) nm; ECD (MeCN) λ_max_ (Δ*ε*) = 192 (+1.20), 203 (+2.29), 223 (−2.01), 237 (−0.77), 253 (−1.26) nm; IR (KBr) *ν*_max_ 3429, 2966, 2921, 2850, 1717, 1657 and 1407 cm^−1^; ^1^H and ^13^C NMR data, see Table 3 and Table 4; for HRESIMS *m*/*z* 344.1835 [M + Na^+^] (calcd. for C_18_H_27_NO_4_Na^+^, 344.1838).

#### 2.3.8. Compound **8**

White amorphous powders; ${\left[\alpha \right]}_{D}^{24}$ −341 (*c* 0.1, MeOH), UV (MeCN) *λ*_max_ (log *ε*) = 210 (4.21), 371 (−0.18) nm; ECD (MeCN) λ_max_ (Δ*ε*) = 205 (+5.16), 252 (−7.58) nm; IR (KBr) *ν*_max_ 3337, 2962, 2929, 1709, 1692, 1670, 1454, 1434, 1416, 701 cm^−1^; for ^1^H and ^13^C NMR data, see Table 3 and Table 4; HRESIMS *m*/*z* 390.2045 [M + Na^+^] (calcd. for C_23_H_29_NO_3_Na^+^, 390.2045).

#### 2.3.9. Compound **9**

Colorless oil; ${\left[\alpha \right]}_{D}^{24}$ +76 (*c* 0.1, MeOH), UV (MeCN) *λ*_max_ (log *ε*) = 196 (4.03) nm; ECD (MeCN) λ_max_ (Δ*ε*) = 199 (+4.53), 215 (−0.25) nm; for ^1^H and ^13^C NMR data, see Table 3 and Table 4; HRESIMS *m*/*z* 259.1668 [M + Na^+^] (calcd. for C_15_H_24_O_2_Na^+^, 259.1674).

#### 2.3.10. Compound **10**

White amorphous powders; ${\left[\alpha \right]}_{D}^{24}$ −10 (*c* 0.1, MeOH), UV (MeCN) *λ*_max_ (log *ε*) = 200 (3.96) nm; ECD (MeCN) λ_max_ (Δ*ε*) = 193 (−16), 200 (−0.35), 208 (−2.54), 222 (+7.60), 236 (+3.35), 250 (−3.18) nm; for ^1^H and ^13^C NMR data, see Table 3 and Table 4; HRESIMS *m*/*z* 277.1784 [M + Na^+^] (calcd. for C_15_H_26_O_3_Na^+^, 277.1780).

#### 2.3.11. Compound **11**

Pale yellow oil; ${\left[\alpha \right]}_{D}^{24}$ +7 (*c* 0.1, MeOH), UV (MeCN) *λ*_max_ (log *ε*) = 210 (4.22), 230 (3.37) nm; ECD (MeCN)λ_max_ (Δ*ε*) = 192 (+2.48), 213 (−0.83), 218 (+0.43), 251 (+0.51), 264 (0.00), 294 (+0.98), 319 (−0.67) nm; for ^1^H and ^13^C NMR data, see Table 5; HRESIMS *m*/*z* 339.1938 [M + Na^+^] (calcd. for C_20_H_28_O_3_Na^+^, 339.1936).

## 3. Materials and Methods

### 3.1. General Experimental Procedures

Thin-layer chromatography (TLC) was performed with silica gel GF254 glass plates (200−250 μm thickness, Qingdao Marine Chemical Inc., Qingdao, China). Compounds were observed by TLC, and spots were visualized by dipping heated silica gel plates with 10% H_2_SO_4_ in EtOH. Column chromatography (CC) was implemented with Sephadex LH-20 (Pharmacia Biotech AB, Uppsala, Sweden), octadecylsilyl (ODS) (50 μm, YMC Co., Ltd., Kyoto, Japan), and silica gel for column chromatography (100−200 mesh and 200−300 mesh; Qingdao Marine Chemical Inc., China). Compounds were purified using a Waters HPLC system using a reversed-phase (RP) C-18 column (5 µm, 10 × 250 mm, QuikSep SP ODS-A, H&E Co., Ltd., Beijing, China). Optical rotations were obtained using a PerkinElmer 341 polarimeter (PerkinElmer Inc., Fremont, CA, USA). ECD spectra were recorded with a JASCO-810 ECD spectrometer (JASCO Corporation, Tokyo, Japan). Ultraviolet–visible spectroscopy (UV) spectra were measured on a Lambda 35 instrument (PerkinElmer Inc., Waltham, MA, USA) in MeOH. Infrared spectroscopy (IR) spectra were recorded with a Vertex 70 FT-IR spectrophotometer (Bruker, Karlsruhe, Germany). The NMR experiments were conducted on Bruker AM-400 or AM-600 spectrometers (Bruker Corporation, Karlsruhe, Germany), and chemical shifts are reported in parts per million (δ) using the DMSO-d_6_ signal (δ_H_ 2.50; δ_C_ 39.5), methanol-d4 signal (δ_H_ 3.31; δ_C_ 49.0), and chloroform-d signal (δ_H_ 7.26; δ_C_ 77.2) as internal standards for ^1^H and ^13^C NMR, respectively. HRESIMS data were acquired using SolariX 7.0T Bruker Daltonics (Bruker Corporation, Karlsruhe, Germany).

### 3.2. Fungus Material

Fungus *Eutypella* sp. MCCC 3A00281 (family: Diatrypaceae) was isolated from the South Atlantic Ocean deep-sea sediment (5610 m) (GPS 6°25′48″ S, 27°54′00″ W, accession number KT366012). The fungal identification was described in a previous publication [11].

### 3.3. Fermentation, Extraction and Isolation

Based on the previously established experimental protocols [19], to obtain the seed culture, *Eutypella* sp. was incubated on potato dextrose agar (PDA) medium at 25 °C for 7 days. Then, the agar was cut into pieces, and the mycelia of the strains grown on PDA were inoculated in autoclaved rice medium (250 g of rice and 250 mL of tap water were placed in 1000 mL Erlenmeyer flasks, with 50 kg of rice in total) and cultured at 25 °C for 35 days. Thereafter, fermented rice was extracted with ethyl alcohol ten times. After the solvent had been evaporated, a nut-brown pasty fluid was obtained, which was evenly dispersed in water and extracted with ethyl acetate eight times. Ultimately, 431 g of crude extract was obtained, which was separated by silica gel column chromatography (100−200 mesh, 800 g) and eluted with a system of petroleum ether-ethyl acetate-methanol (10:1:0–10:10:0–10:10:1, *v*/*v*/*v*) to afford six primary fractions (Fr.1–Fr.6).

Fr.4 (86.3 g) was subjected to ODS column chromatography (CC, MeOH–H_2_O, 40–90%) to obtain 7 fractions (Fr.4.1–Fr.4.7). Fr.4.4 was submitted to silica gel CC (petroleum ether–ethyl acetate) to obtain three fractions (Fr.4.4.1–Fr.4.4.3). Compound **1** (1.0 mg; MeOH-H_2_O, 64%, *v*/*v*, *t*_R_ = 33 min, 3.0 mL/min), compound **4** (9.2 mg; MeCN-H_2_O, 48%, *v*/*v*, *t*_R_ = 25 min, 3.0 mL/min), compound **8** (2.4 mg; MeOH-H_2_O, 70%, *v*/*v*, *t*_R_ = 30 min, 3.0 mL/min), and compound **9** (1.0 mg; MeOH-H_2_O, 80%, *v*/*v*, *t*_R_ = 35 min, 2.0 mL/min) were purified by semipreparative high-performance liquid chromatography (HPLC) from Fr.4.4.1. Fr.4.4.2 was subjected to Sephadex LH-20, silica gel CC, and HPLC purification, isolating compound **2** (5.0 mg; MeOH-H_2_O, 57%, *v*/*v*, *t*_R_ = 67 min, 3.0 mL/min), compound **3** (4.1 mg; MeOH-H_2_O, 59%, *v*/*v*, *t*_R_ = 40 min, 3.0 mL/min), compound **5** (1.0 mg; MeOH-H_2_O, 61%, *v*/*v*, *t*_R_ = 40 min, 3.0 mL/min), and compound **11** (1.0 mg; MeOH-H_2_O, 60%, *v*/*v*, *t*_R_ = 76 min, 3.0 mL/min). Fr.5 (24.0 g) was subjected to ODS column chromatography (CC, MeOH−H_2_O, 20–100%) to obtain 9 fractions (Fr.5.1–Fr.5.9). Compound **6** (6.0 mg; MeCN-H_2_O, 24%, *v*/*v*, *t*_R_ = 38 min, 3.0 mL/min), compound **7** (10.0 mg; MeCN-H_2_O, 33%, *v*/*v*, *t*_R_ = 43 min, 3.0 mL/min), and compound **10** (0.8 mg; MeOH-H_2_O, 65%, *v*/*v*, *t*_R_ = 38 min, 3.0 mL/min) were purified by semipreparative HPLC from Fr.5.3.

The compounds isolated in this study are terpenoids, exhibiting Rf values of 0.4–0.7 (developed in dichloromethane/methanol, 20:1, *v*/*v*), with bluish-purple spots observed after spraying with the detection reagent.

### 3.4. X-Ray Crystal Structure Analysis

Suitable crystals of compounds **4** and **7** were obtained by slow evaporation from MeOH–THF (10:1) at 20 °C. The intensity data for these compounds were collected with a Bruker APEX DUO diffractometer (Bruker Corporation, Karlsruhe, Germany) equipped with an APEX II CCD using graphite-monochromated Cu Kα radiation. Crystallographic data for the reported structures were deposited in the Cambridge Crystallographic Data Center (CCDC) with deposition number CCDC 2431789 for compound **4** and CCDC 2431799 for compound **7**.

Crystal Data for Compound **4**. C_15_H_26_O_3_, *M* = 254.36 g/mol, *a* = 11.79120 (10) Å, *b* = 8.47850 (10) Å, *c* = 15.1542(2) Å, *α* = 90°, *β* = 106.3940 (10)°, *γ* = 90°, *V* = 1453.40 (3) Å^3^, *T* = 99.99 (10) K, space group C_2_, *Z* = 4, *μ* (Cu Kα) = 0.627 mm^−1^, 7208 reflections measured, 2842 independent reflections (R*int* = 0.0409). The final *R*_1_ value was 0.0365 (*I* > 2*σ*(*I*)). The final *wR*(*F*^2^) value was 0.0978 (I > 2*σ*(I)). The final *R*_1_ value was 0.0372 (all data). The final wR(*F*^2^) value was 0.0983 (all data). The goodness of fit on *F*^2^ was 1.063. The flack parameter was 0.03 (12).

Crystal Data for Compound **7**. C_18_H_27_NO_4_, *M* = 321.19 g/mol, *a* = 7.07560 (10) Å, *b* = 12.18480 (10) Å, *c* = 23.0375 (2) Å, *α* = 90°, *β* = 90°, *γ* = 90°, *V* = 1986.17 (4) Å^3^, *T* = 99.99 (10) K, space group P2_1_2_1_2_1_, *Z* = 4, *μ* (Cu Kα) = 0.794 mm^−1^, 20,759 reflections measured, 4009 independent reflections (*R_int_* = 0.0427). The final *R*_1_ value was 0.0277 (*I* > 2*σ*(*I*)). The final *wR*(*F*^2^) value was 0.0698 (*I* > 2*σ*(*I*)). The final *R*_1_ value was 0.0285 (all data). The final *wR*(*F*^2^) value was 0.0702 (all data). The goodness of fit on *F*^2^ was 1.048. The flack parameter was 0.01 (6).

### 3.5. ECD Calculations

In general, conformational analyses were carried out via random searching in the Sybyl-X 2.0 using the MMFF94S force field with an energy cutoff of 5 kcal/mol. The results showed the nine lowest energy conformers for compound **1**, the seven lowest energy conformers for compound **2**, the five lowest energy conformers for compound **3**, the ten lowest energy conformers for compound **5**, the two lowest energy conformers for compound **6**, the thirty lowest energy conformers for compound **8**, the nine lowest energy conformers for compound **9**, the twenty lowest energy conformers for compound **10**, and the nine lowest energy conformers for compound **11**. Subsequently, geometry optimizations and frequency analyses were implemented at the B3LYP-D3(BJ)/6-31G* level in conductor-like polarizable continuum model (CPCM) methanol using ORCA5.0.1 [20]. All conformers used for property calculations in this work were characterized to be stable on a potential energy surface (PES) with no imaginary frequencies. The excitation energies, oscillator strengths, and rotational strengths (velocity) of the first 60 excited states were calculated using the TD-DFT methodology at the PBE0/def2-TZVP level in CPCM methanol using ORCA5.0.1 [20]. The ECD spectra were simulated by the overlapping Gaussian function (half the bandwidth at 1/e peak height, sigma = 0.30 for all) [21]. Gibbs free energies for conformers were determined by using thermal correction at B3LYP-D3(BJ)/6-31G* level and electronic energies evaluated at the wB97M-V/def2-TZVP level in CPCM methanol using ORCA5.0.1 [20]. To obtain the final spectra, the simulated spectra of the conformers were averaged according to the Boltzmann distribution theory and their relative Gibbs free energy (ΔG). By comparing the experiment spectra with the calculated model molecules, the absolute configuration of the chiral center was determined.

### 3.6. ^13^C NMR Calculations

The details of NMR calculations with DP4+ probability analyses for compound **10** are included in the Supporting Information.

### 3.7. Biological Activity

Animals: Male BALB/c mice (6–8 weeks old) were obtained from Hubei Biont Biotechnology (Wuhan, China) and were fed in the Tongji Experimental Animal Center. The mice were raised under conditions of a 12 h light/12 h dark cycle, a temperature of 22 ± 1 °C, and a relative humidity of 55 ± 5%. All the mice were acclimatized for 1 week before use. All experiments were performed in accordance with the guidelines of the Ethical Committee for the Experimental Use of Animals at Huazhong University of Science and Technology (Wuhan, China).

Lymphocyte preparation: Briefly, the mice were sacrificed by cervical dislocation, and the spleen was dissected out in a sterile environment. The spleen was ground in a 70 μm filter to obtain a single-cell suspension. After centrifugation at 300× *g* for 5 min, an appropriate amount of lysis buffer was added to lyse the red blood cells for 5 min. The cells were collected after centrifugation at 300× *g* for 5 min and washed twice with PBS. Lymphocytes were lysed in RPMI1640 (Gibco, Thermo Fisher Scientific, Waltham, MA, USA) supplemented with 10% FBS and penicillin–streptomycin solution culture medium. The cells were counted on an automatic counter, and the cell concentration was adjusted to 5 × 10^6^ mL^−1^. Cell viability was determined by trypan-blue dye exclusion, and cell viability reached up to 95%. The culture media were maintained in a humidified atmosphere of 5% CO_2_ at 37 °C.

Lymphocyte proliferation assay: The splenocytes (5 × 10^5^ cells per well) were cultured in 96-well plates in triplicate in the presence of ConA (5 μg mL^−1^), or LPS (10 μg mL^−1^) medium in a humidified atmosphere of 5% CO_2_ at 37 °C. Next, the corresponding final concentration of the compound was added to the lymphocytes. After 48 h, 10 μL of CCK-8 was added to each well and the plates were incubated for 1 h. The absorbance was measured on an ELISA microplate reader at a wavelength of 450 nm [22].

## 4. Conclusions

In summary, a total of ten undescribed sesquiterpenes with two types of skeletons, including eight undescribed eremophilane-type sesquiterpenes (**1**–**8**), two undescribed sub-type of sesquiterpenes (**9**–**10**), and an abietane-type diterpenoid (**11**), were isolated and identified from the deep-sea fungus *Eutypella* sp. MCCC 3A00281. Their structures were elucidated on the basis of various spectroscopic analyses, mainly including NMR and HRESIMS data, ^13^C NMR calculations with DP4+ probability analyses, ECD calculations, and single-crystal X-ray diffraction experiments. Immunosuppressive activity assays were performed on all isolated compounds. The results showed that compound 11 exhibited potent immunosuppressive activity with IC_50_ values of 8.99 ± 1.08 μM (LPS model) and 5.39 ± 0.20 μM (ConA model). Compounds 1 and 9 exhibited moderate inhibitory activity in the ConA model with IC_50_ values of 13.55 ± 1.40 μM and 29.25 ± 1.56 μM, respectively. Regrettably, there are usually low contents of novel compounds in fungi, and sufficient quantities have not been obtained for more comprehensive and in-depth research on interesting biological activities.

## Figures and Tables

**Table 1:** ^1^H NMR data (*δ* in ppm, *J* in Hz) for compounds **1**–**5**.

No.	1 ^a,d^	2 ^a,c^	3 ^a,c^	4 ^b,d^	5 ^a,c^
1	5.38,1H, d (4.9)	1.99, 1H, ddt (13.1, 11.2, 2.0), ax 2.19, 1H, ddd (13.1, 4.9, 2.2), eq	1.04, 1H, dd (12.8, 11.1), ax 1.40, 1H, m, eq	5.36, 1H, m	3.53, 1H, dt (9.4, 4.6), ax
2	3.84, 1H, d (5.2), eq	3.34, 1H, d (4.9), ax	3.39, 1H, dp (10.1, 5.1), ax	1.96, 2H, m, ax	1.33 *, 1H, eq 1.63 *, 1H, ax
3	1.35, 1H, d (13.6), eq 1.49, 1H, td (13.4, 4.4), ax	1.25, 1H, td (12.3, 10.6), ax 1.60, 1H, ddt (12.2, 5.0, 2.4), eq	1.52, 1H, ddd (9.5, 5.0, 1.9), eq 1.13, 1H, m, ax	1.40, 2H, dp (9.0, 4.2)	1.30 *, 1H, ax 1.43, 1H, m, eq
4	1.64, 1H, m, ax	1.30, 1H, m, ax	1.09 *, 1H, ax	1.33, 1H, m, ax	1.69 *, 1H
5					
6	1.06, 1H, t (13.1), ax 1.61, 1H, dd (13.1, 3.5), eq	1.45 *, 1H, ax 1.47 *, 1H, eq	1.44 *, 1H, eq 0.91, 1H, t (12.9), ax	1.83, 1H, dd (13.0, 3.2) 0.78, 1H, t (13.0)	2.37, 1H, d (14.8) 2.44, 1H, d (14.8)
7	2.18 *, 1H, ax	2.10, 1H, dt (9.2, 4.3), ax	2.05, 1H, ddd (13.5, 10.3, 3.7), ax	1.90, 1H, m, ax	
8	3.26, 1H, m	3.92, 1H, m	3.29, 1H, dt (10.5, 5.2)	3.73 *, 1H	
9	2.21 *, 2H	5.45, 1H, dd (5.5, 1.8)	1.45 *, 1H, eq 1.24, 1H, td (12.3, 10.6), ax	2.31, 2H, m	2.31, 1H, d (16.4) 2.66, 1H, d (16.4)
10			1.10 *, 1H, ax		
11					
12	4.72, 2H, brs	4.66, 1H, brs 4.76, 1H, brs	4.67, 2H, brs	3.75 *, 2H	1.81, 3H, brs
13	1.65, 3H, s	1.75, 3H, s	1.63, 3H, s	1.19, 3H, s	1.75, 3H, brs
14	0.84, 3H, s	0.85, 3H, s	0.68, 3H, s	0.95, 3H, s	0.83, 3H, s
15	0.80, 3H, d (6.6)	0.86, 3H, brs	0.72, 3H, d (5.9)	0.88, 3H, d (6.4)	0.77, 3H, d (6.6)
1-OH					4.35, 3H, d (4.3)
2-OH	4.43, 1H, d (5.3)	4.65, 1H, brs	4.43, 1H, d (4.7)		
8-OH	4.45, 1H, d (5.6)	4.06, 1H, d (6.6)	4.19, 1H, d (5.6)		
10-OH					4.45, 3H, s

* overlapped; ^a^ DMSO-*d*_6_; ^b^ CDCl_3_; ^c^ 600 MHz; ^d^ 400 MHz.

**Table 2:** ^13^C NMR data (*δ* in ppm) for compounds **1**–**5**.

No.	1 ^a,c^	2 ^a,c^	3 ^a,c^	4 ^b,d^	5 ^a,c^
1	124.2, CH	41.7, CH_2_	40.0, CH_2_	122.6, CH	71.3, CH
2	62.3, CH	68.9, CH	68.7, CH	30.0, CH_2_	30.9, CH_2_
3	36.2, CH_2_	39.9, CH_2_	40.1, CH_2_	27.2, CH_2_	28.4, CH_2_
4	33.9, CH	40.4, CH	41.8, CH	40.7, CH	32.4, CH
5	37.2, C	37.5, C	35.4, C	37.5, C	42.2, C
6	42.7, CH_2_	34.7, CH_2_	42.9, CH_2_	39.2, CH_2_	35.9, CH_2_
7	48.5, CH	42.1, CH	48.6, CH	47.7, CH	131.1, C
8	71.2, CH	63.9, CH	70.9, CH	73.9, CH	203.4, C
9	41.6, CH_2_	124.4, CH	37.8, CH_2_	42.6, CH_2_	45.0, CH_2_
10	143.9, C	144.2, C	40.6, CH	140.2, C	78.3, C
11	147.4, C	147.1, C	148.0, C	76.4, C	137.7, C
12	111.2, CH_2_	110.0, CH_2_	110.9, CH_2_	70.0, CH_2_	22.2, CH_3_
13	19.5, CH_3_	22.2, CH_3_	19.6, CH_3_	24.4, CH_3_	21.2, CH_3_
14	16.5, CH_3_	16.4, CH_3_	11.2, CH_3_	18.3, CH_3_	14.2, CH_3_
15	15.5, CH_3_	15.4, CH_3_	15.3, CH_3_	16.1, CH_3_	16.0, CH_3_

^a^ DMSO-*d*_6_; ^b^ CDCl_3_; ^c^ 150 MHz; ^d^ 100 MHz.

**Table 3:** ^1^H NMR data (*δ* in ppm, *J* in Hz) for compounds **6**–**10**.

No.	6 ^b,c^	7 ^b,d^	8 ^b,d^	9 ^b,c^	10 ^a,c^
1	3.49 *, 1H, ax			3.55, 1H, m 3.68, 1H, td (8.4, 7.3, 4.3)	3.25, 1H, m 3.46, 1H, ddd (11.3, 5.2, 2.7)
2	1.39, 1H, q (11.5), eq 2.39, 3H, dt (11.8, 4.6), ax	2.38 *, 1H 2.42 *, 1H	2.37 *, 1H 2.41 *, 1H	3.81, 1H, ddd (10.0, 6.8, 2.9), ax	3.41, 1H, m, ax
3	3.49 *, 3H, ax	1.66, 1H, td (13.2, 6.0), ax 1.92 *, 1H, eq	1.63 *, 1H, ax 1.92, 1H, m, eq		
4	1.25, 1H, dd (10.3, 6.7), ax	2.04, 1H, ddd (12.6, 6.6, 3.9), ax	2.02, 1H, ddd (12.6, 6.8, 4.0), eq	5.28, 1H, s	5.25 *, 1H
5				1.92, 1H, t (12.9) 2.04, 1H, m	1.86 *, 1H, ax 2.12, 1H, m, eq
6	1.96, 1H, m, ax 2.74, 1H, d (15.0), eq	1.94 *, 1H, ax 2.62, 1H, d (13.0), eq	2.17, 1H, d (13.1), ax 2.54, 1H, d (13.1), eq	1.71, 1H, m, ax	2.27, 1H, td (10.0, 8.3), ax
7					
8				1.32, 1H, t (11.9) 1.54 *	3.31, 1H, m
9	2.19, 1H, dd (17.3, 12.7) 2.80, 1H, dd (17.3, 5.5	1.48, 1H, dd (14.0, 12.5), ax 2.27, 1H, dd (14.0, 3.4, eq	1.35, 1H, m, ax 2.26, 1H, dd (14.0, 3.4), eq	4.54, 1H, ddd (11.3, 8.1, 2.9), ax	1.84 *, 1H 1.92, 1H, m
10	1.65, 1H, ddd (12.6, 10.2, 5.5), ax	2.75, 1H, dd (12.5, 3.4), ax	2.73, 1H, dd (12.5, 3.3), ax	5.19, 1H, d (8.0)	5.25 *, 1H
11					
12	2.03, 3H, d (2.2)			1.73, 3H, s	1.65, 3H, s
13	1.79, 3H, d (1.4)	1.84, 3H, d (1.6)	1.77, 3H, d (1.5)	1.69, 3H, s	1.53, 3H, s
14	0.75, 3H, s	0.51, 3H, s	0.47, 3H, s	0.95, 3H, s	0.96, 3H, s
15	1.08, 3H, d (6.7)	1.02, 3H, d (6.7)	1.00, 3H, d (6.7)	1.65, 3H, s	1.51, 3H, s
16		2.89, 3H, s	3.72, 1H, dddd (14.2, 8.9, 5.2, 1.3) 3.45, 1H, ddd (13.9, 8.8, 7.2)		
17		3.40, 1H, ddd (14.8, 6.0, 4.0) 3.51, 1H, m	2.91, 1H, ddd (13.7, 8.8, 5.3) 3.11, 1H, ddd (13.2, 8.9, 7.1)		
18		3.79, 2H, brs			
19, 23			7.29 *, 2H		
20, 22			7.22 *, 2H		
21			7.21 *, 1H		

* overlapped; ^a^ DMSO-*d*_6_; ^b^ CDCl_3_; ^c^ 600 MHz; ^d^ 400 MHz.

**Table 4:** ^13^C NMR data (*δ* in ppm) for compounds **6**–**10**.

No.	6 ^b,c^	7 ^b,d^	8 ^b,c^	9 ^b,c^	10 ^a,c^
1	69.5, CH	210.6, C	210.8, C	65.4, CH_2_	65.0, CH_2_
2	45.2, CH_2_	41.3, CH_2_	41.4, CH_2_	75.8, CH	71.1, CH
3	70.5, CH	31.3, CH_2_	31.3, CH_2_	150.9, C	145.7, C
4	49.6, CH	42.3, CH	42.2, CH	122.5, CH	123.1, CH
5	37.1, C	44.9, C	44.9, C	29.4, CH_2_	32.2, CH_2_
6	43.5, CH_2_	36.9, CH_2_	36.1, CH_2_	50.7, CH	44.6, CH
7	129.8, C	148.2, C	150.3, C	45.6, C	55.4, C
8	202.9, C	91.6, C	87.6, C	41.7, CH_2_	74.7, CH
9	40.9, CH_2_	32.8, CH_2_	32.5, CH_2_	70.3, CH	31.8, CH_2_
10	50.0, CH	53.6, CH	53.7, CH	126.6, CH	123.4, CH
11	145.6, C	130.6, C	128.1, C	135.3, C	130.8, C
12	23.5, CH_3_	172.6, C	170.6, C	25.9, CH_3_	25.7, CH_3_
13	22.6, CH_3_	8.3, CH_3_	8.2, CH_3_	18.5, CH_3_	17.8, CH_3_
14	12.8, CH_3_	11.7, CH_3_	11.4, CH_3_	15.5, CH_3_	16.9, CH_3_
15	10.9, CH_3_	14.8, CH_3_	14.8, CH_3_	12.3, CH_3_	13.0, CH_3_
16		49.8, OCH_3_	40.8, OCH_2_		
17		42.7, CH_2_	35.2, CH_2_		
18		63.0, CH_2_	139.7, C		
19, 23			128.7, CH		
20, 22			129.1, CH		
21			126.6, CH		

^a^ DMSO-*d*_6_; ^b^ CDCl_3_; ^c^ 150 MHz; ^d^ 100 MHz.

**Table 5:** ^1^H and ^13^C NMR data (*δ* in ppm, *J* in Hz) for compound **11** (CD_3_OD).

No.	11		Hydroxyldecandrin G	
	*δ*_H_^a^	*δ*_C_^b^	*δ*_H_	*δ*_C_
1	1.48, 1H, t (12.1)	45.6, CH_2_	1.48 t (12.1)	45.6
	2.57, 1H, dd (12.1, 4.6)		2.57 dd (12.1, 4.6)	
2	3.75, 1H, ddd (12.1, 9.7, 4.6), ax	69.2, CH	3.75 ddd (12.1, 9.7, 4.6)	69.2
3	2.94 1H, d (9.7), ax	83.4, CH	2.95 d (9.7)	83.4
4		40.4, C		40.4
5	1.83 1H, dd (13.7, 4.1), ax	50.0, CH	1.83 dd (13.9, 4.0)	50.1
6	2.59 1H, dd (18.2, 4.1), eq	36.8, CH_2_	2.59 dd (18.1, 4.0);	36.9
	2.68 1H, dd (18.2, 13.7), ax		2.68 dd (18.1,13.9)	
7		201.3, C		201.2
8		131.3, C		131.1
9		154.4, C		154.9
10		39.8, C		39.9
11	7.29, 1H, d (8.2)	125.6, CH	7.33 d (8.3)	125.0
12	7.40, 1H, dd (8.2, 2.2)	134.1, CH	7.64 dd (8.3, 2.2)	132.4
13		148.4, C		149.4
14	7.72, 1H, d (2.2)	125.2, CH	7.98 d (2.2)	124.1
15	2.84, 1H, m	34.8, CH		72.6
16	1.15, 3H, d (6.9)	24.2, CH_3_	1.42 s	31.7
17	1.15, 3H, d (6.9)	24.2, CH_3_	1.42 s	31.7
18	0.98, 3H, s	28.5, CH_3_	0.98 s	28.5
19	0.90, 3H, s	16.7, CH_3_	0.88 s	16.7
20	1.20, 3H, s	24.6, CH_3_	1.20 s	24.6

^a^ 600 MHz; ^b^ 150MHz.

## Data Availability

The original contributions presented in this study are included in the article’s Supplementary Materials. This article contains 94 Supplementary Figures and 4 Supplementary Tables. Further inquiries can be directed to the corresponding author.

## Supplementary Materials

The following supporting information can be downloaded at https://www.mdpi.com/article/10.3390/ph18050737/s1. Supplementary data associated with this article include HRESIMS, 1D and 2D NMR, IR, UV, and ECD spectra for compounds **1**–**11**. References [20,23,24,25,26] are cited in the Supplementary Materials.
