# Supplementary material for: Isolation of Ten New Sesquiterpenes and New Abietane-Type Diterpenoid with Immunosuppressive Activity from Marine Fungus Eutypella sp
Source: Pharmaceuticals (Basel). 2025 May 16;18(5):737. doi: 10.3390/ph18050737 (PMC12114701; doi:10.3390/ph18050737)
Supplement: Supplementary file 1 [file pharmaceuticals-18-00737-s001.zip › Revised-Supporting Information.pdf]

Supporting Information

Isolation of ten new sesquiterpenes and a new abietane-type diterpenoid with immunosuppressive activity from a marine fungus *Eutypella* sp.

List of Supporting Information

Figure S1. UV spectrum of compound **1** in MeCN. 6

Figure S2. ^{1}H NMR spectrum of compound **1** (400 MHz, DMSO-d_6). 6

Figure S3. ^{13}C and DEPT-135 NMR spectra of compound **1** (150 MHz, DMSO-d_6). 7

Figure S4. HSQC spectrum of compound **1** (DMSO-d_6). 7

Figure S6. HMBC spectrum of compound **1** (DMSO-d_6). 8

Figure S7. NOESY spectrum of compound **1** (DMSO-d_6). 9

Figure S8. HRESIMS spectrum of compound **1**. 9

Figure S9. UV spectrum of compound **2** in MeCN. 10

Figure S10. IR spectrum of compound **2**. 10

Figure S11. ^{1}H NMR spectrum of compound **2** (600 MHz, DMSO-d_6). 11

Figure S12. ^{13}C and DEPT-135 NMR spectra of compound **2** (150 MHz, DMSO-d_6). 11

Figure S13. HSQC spectrum of compound **2** (DMSO-d_6). 12

Figure S14. ^{1}H–^{1}H COSY spectrum of compound **2** (DMSO-d_6). 12

Figure S15. HMBC spectrum of compound **2** (DMSO-d_6). 13

Figure S16. NOESY spectrum of compound **2** (DMSO-d_6). 13

Figure S17. HRESIMS spectrum of compound **2**. 14

Figure S18. UV spectrum of compound **3** in MeCN. 14

Figure S19. IR spectrum of compound **3**. 15

Figure S20. ^{1}H NMR spectrum of compound **3** (600 MHz, DMSO-d_6). 15

Figure S21. ^{13}C and DEPT-135 NMR spectra of compound **3** (150 MHz, DMSO-d_6). 16

Figure S22. HSQC spectrum of compound **3** (DMSO-d_6). 16

Figure S23. ^{1}H–^{1}H COSY spectrum of compound **3** (DMSO-d_6). 17

Figure S24. HMBC spectrum of compound **3** (DMSO-d_6). 17

Figure S25. NOESY spectrum of compound **3** (DMSO-d_6). 18

Figure S26. HRESIMS spectrum of compound **3**. 18

Figure S27. UV spectrum of compound **4** in MeCN. 19

Figure S28. IR spectrum of compound **4**. 19

Figure S29. ^{1}H NMR spectrum of compound **4** (400 MHz, $CDCl_3$)....................................20
Figure S30. ^{13}C and DEPT-135 NMR spectra of compound **4** (100 MHz, $CDCl_3$)...........20
Figure S31. HSQC spectrum of compound **4** ($CDCl_3$)..21
Figure S32. ^{1}H–^{1}H COSY spectrum of compound **4** ($CDCl_3$)..21
Figure S33. HMBC spectrum of compound **4** ($CDCl_3$)..22
Figure S34. NOESY spectrum of compound **4** ($CDCl_3$). 22
Figure S35. HRESIMS spectrum of compound **4**. 23
Figure S36. UV spectrum of compound **5** in MeCN...23
Figure S37. ^{1}H NMR spectrum of compound **5** (600 MHz, DMSO-d_6)..............................24
Figure S38. ^{13}C and DEPT-135 NMR spectra of compound **5** (150 MHz, DMSO-d_6). ... 24
Figure S39. HSQC spectrum of compound **5** (DMSO-d_6)...25
Figure S40. ^{1}H–^{1}H COSY spectrum of compound **5** (DMSO-d_6)..25
Figure S41. HMBC spectrum of compound **5** (DMSO-d_6)..26
Figure S42. NOESY spectrum of compound **5** (DMSO-d_6). 26
Figure S43. HRESIMS spectrum of compound **5**. 27
Figure S44. UV spectrum of compound **6** in MeCN...27
Figure S45. IR spectrum of compound **6**...28
Figure S46. ^{1}H NMR spectrum of compound **6** (600 MHz, $CDCl_3$)....................................28
Figure S47. ^{13}C and DEPT-135 NMR spectra of compound **6** (150 MHz, $CDCl_3$)...........29
Figure S48. HSQC spectrum of compound **6** ($CDCl_3$)..29
Figure S49. ^{1}H–^{1}H COSY spectrum of compound **6** ($CDCl_3$)..30
Figure S50. HMBC spectrum of compound **6** ($CDCl_3$)..30
Figure S51. NOESY spectrum of compound **6** ($CDCl_3$). 31
Figure S52. HRESIMS spectrum of compound **6**. 31
Figure S53. UV spectrum of compound **7** in MeCN...32
Figure S54. IR spectrum of compound **7**...32
Figure S55. ^{1}H NMR spectrum of compound **7** (400 MHz, $CDCl_3$)....................................33
Figure S56. ^{13}C and DEPT-135 NMR spectra of compound **7** (100 MHz, $CDCl_3$)...........33
Figure S57. HSQC spectrum of compound **7** ($CDCl_3$)..34

Figure S58. ^1H—^1H COSY spectrum of compound **7** ($CDCl_3$).........34
Figure S59. HMBC spectrum of compound **7** ($CDCl_3$).........35
Figure S60. NOESY spectrum of compound **7** ($CDCl_3$).........35
Figure S61. HRESIMS spectrum of compound **7**.........36
Figure S62. UV spectrum of compound **8** in MeCN.........36
Figure S63. IR spectrum of compound **8**.........37
Figure S64. ^1H NMR spectrum of compound **8** (400 MHz, $CDCl_3$).........37
Figure S65. ^{13}C and DEPT-135 NMR spectra of compound **8** (150 MHz, $CDCl_3$).........38
Figure S66. HSQC spectrum of compound **8** ($CDCl_3$).........38
Figure S67. ^1H—^1H COSY spectrum of compound **8** ($CDCl_3$).........39
Figure S68. HMBC spectrum of compound **8** ($CDCl_3$).........39
Figure S69. NOESY spectrum of compound **8** ($CDCl_3$).........40
Figure S70. HRESIMS spectrum of compound **8**.........40
Figure S71. UV spectrum of compound **9** in MeCN.........41
Figure S72. ^1H NMR spectrum of compound **9** (600 MHz, $CDCl_3$).........41
Figure S73. ^{13}C and DEPT-135 NMR spectra of compound **9** (150 MHz, $CDCl_3$).........42
Figure S74. HSQC spectrum of compound **9** ($CDCl_3$).........42
Figure S75. ^1H—^1H COSY spectrum of compound **9** ($CDCl_3$).........43
Figure S76. HMBC spectrum of compound **9** ($CDCl_3$).........43
Figure S77. NOESY spectrum of compound **9** ($CDCl_3$).........44
Figure S78. HRESIMS spectrum of compound **9**.........44
Figure S79. UV spectrum of compound **10** in MeCN.........45
Figure S80. ^1H NMR spectrum of compound **10** (600 MHz, $CDCl_3$).........45
Figure S81. ^{13}C and DEPT-135 NMR spectra of compound **10** (150 MHz, $CDCl_3$).........46
Figure S82. HSQC spectrum of compound **10** ($CDCl_3$).........46
Figure S83. ^1H—^1H COSY spectrum of compound **10** ($CDCl_3$).........47
Figure S84. HMBC spectrum of compound **10** ($CDCl_3$).........47
Figure S85. NOESY spectrum of compound **10** ($CDCl_3$).........48
Figure S86. HRESIMS spectrum of compound **10**.........48

Figure S87. UV spectrum of compound **11** in MeCN. 49

Figure S88. ^{1}H NMR spectrum of compound **11** (600 MHz, CD_3OD)........ 49

Figure S89. ^{13}C and DEPT-135 NMR spectra of compound **11** (150 MHz, CD_3OD)....... 50

Figure S90. HSQC spectrum of compound **11** (CD_3OD). 50

Figure S91. ^{1}H—^{1}H COSY spectrum of compound **11** (CD_3OD). 51

Figure S92. HMBC spectrum of compound **11** (CD_3OD)........ 51

Figure S93. NOESY spectrum of compound **11** (CD_3OD). 52

Figure S94. HRESIMS spectrum of compound **11**........ 52

^{13}C NMR Calculation Data of 10........ 53

Table S1. Gibbs free energies[a] and equilibrium populations[b] of low-energy conformers of **10A**. 54

Table S2. Cartesian coordinates for the low-energy reoptimized random reseach conformers of **10A** at B3LYP-D3(BJ)/6-31G* level of theory in chloroform. 55

Table S3. Gibbs free energies[a] and equilibrium populations[b] of low-energy conformers of **10B**. 77

Table S4. Cartesian coordinates for the low-energy reoptimized random reseach conformers of **10B** at B3LYP-D3(BJ)/6-31G* level of theory in chloroform........ 78

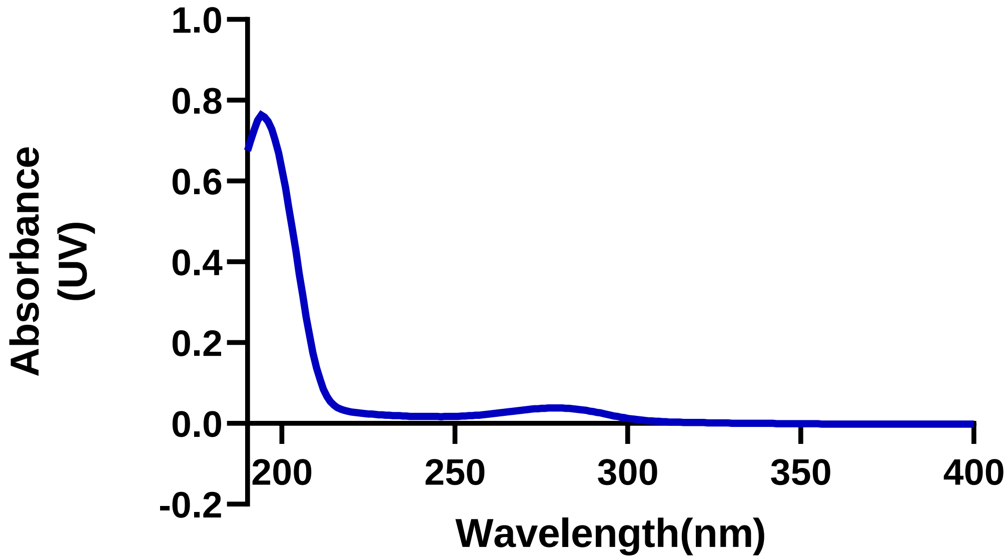

Figure S1. UV spectrum of compound **1** in MeCN.

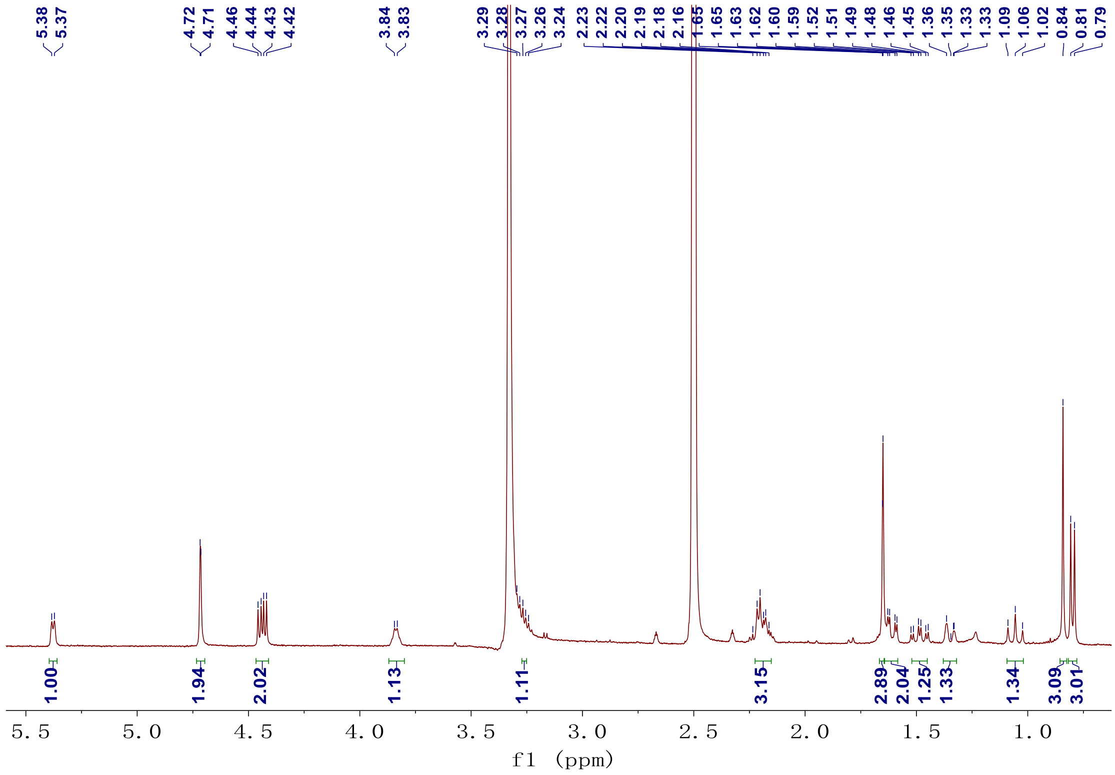

Figure S2. ^{1}H NMR spectrum of compound **1** (400 MHz, DMSO-d_6).

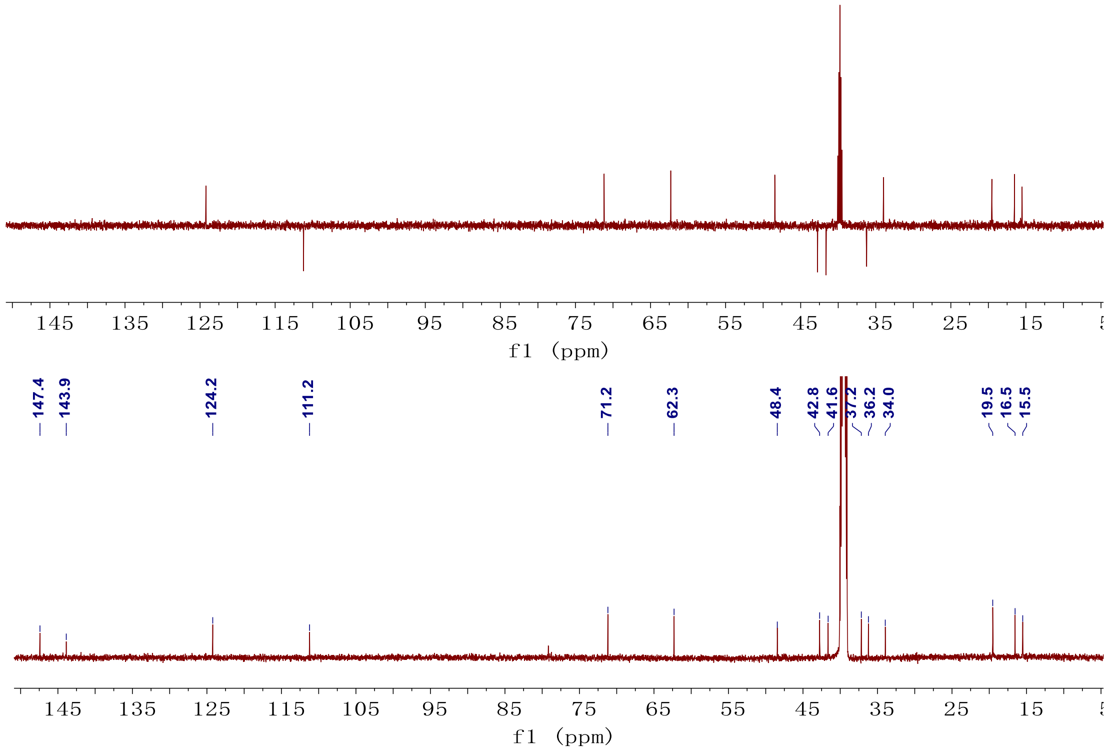

Figure S3. ^{13}C and DEPT-135 NMR spectra of compound **1** (150 MHz, DMSO-d_6).

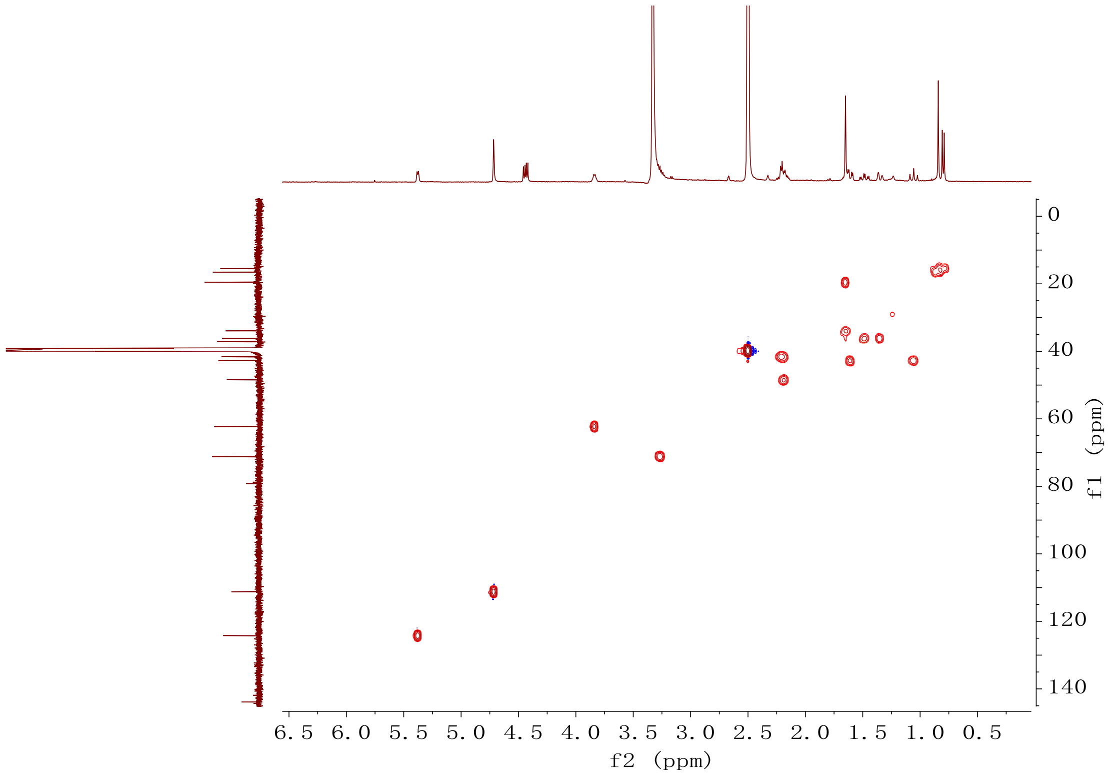

Figure S4. HSQC spectrum of compound **1** (DMSO-d_6).

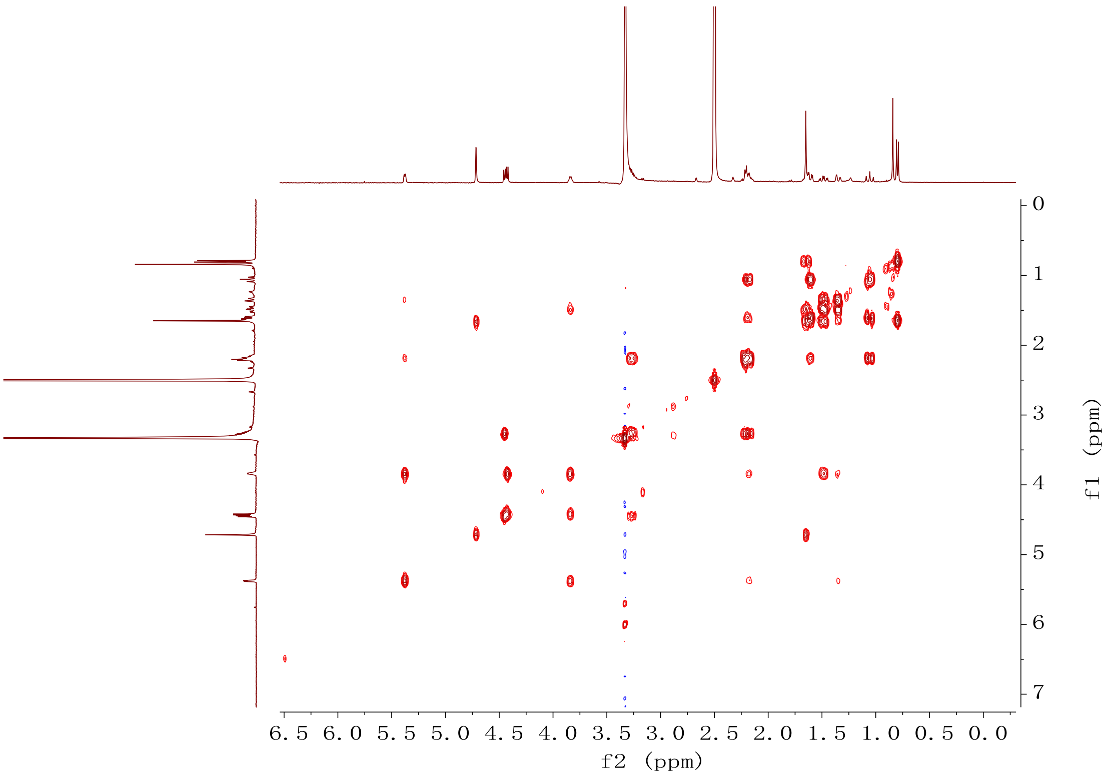

Figure S5. ^{1}H–^{1}H COSY spectrum of compound **1** (DMSO-d_6).

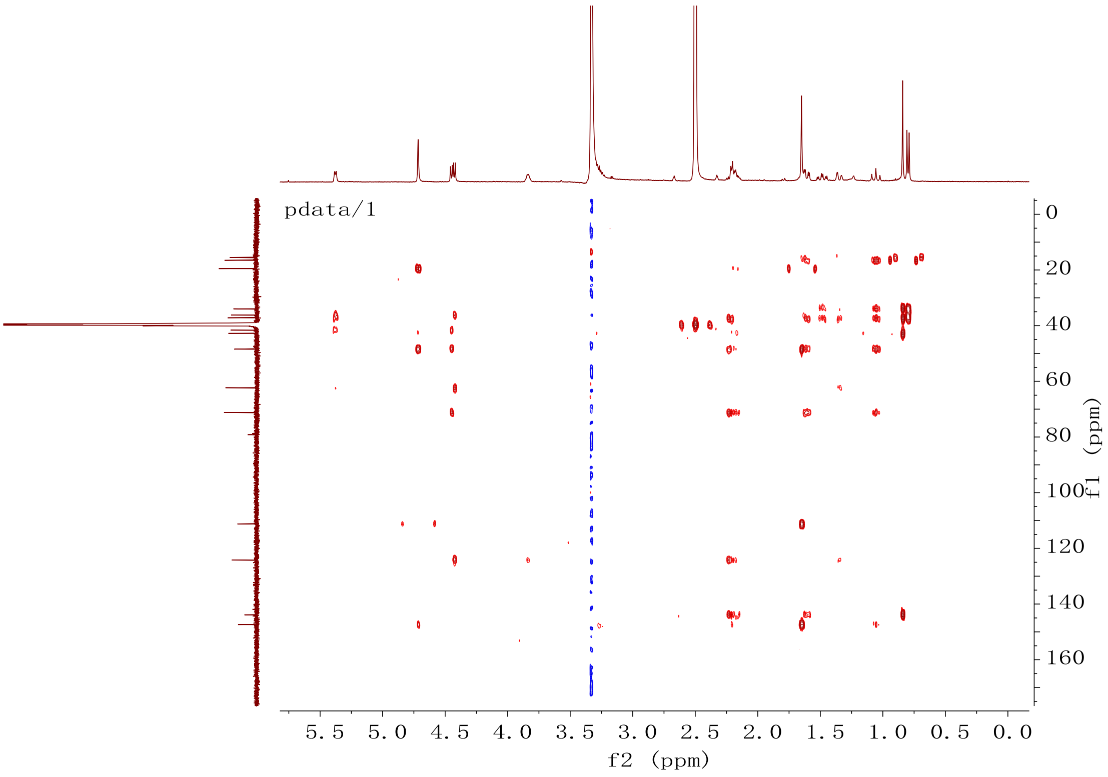

Figure S6. HMBC spectrum of compound **1** (DMSO-d_6).

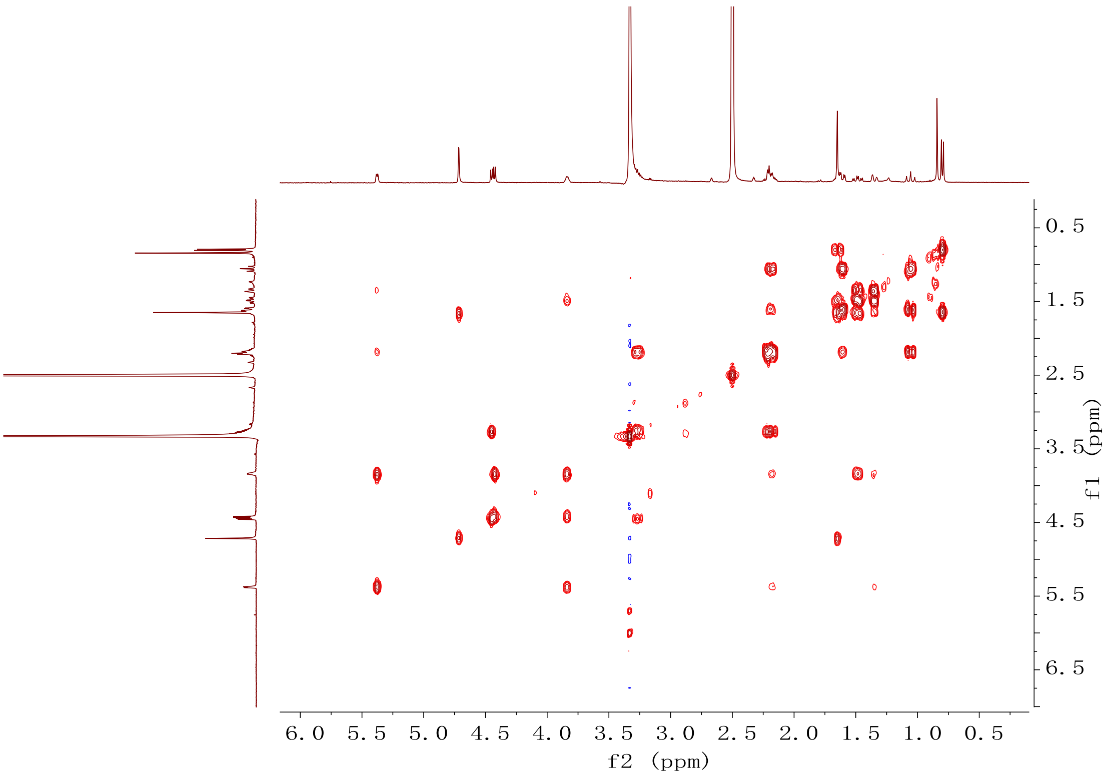

Figure S7. NOESY spectrum of compound **1** (DMSO-d_6).

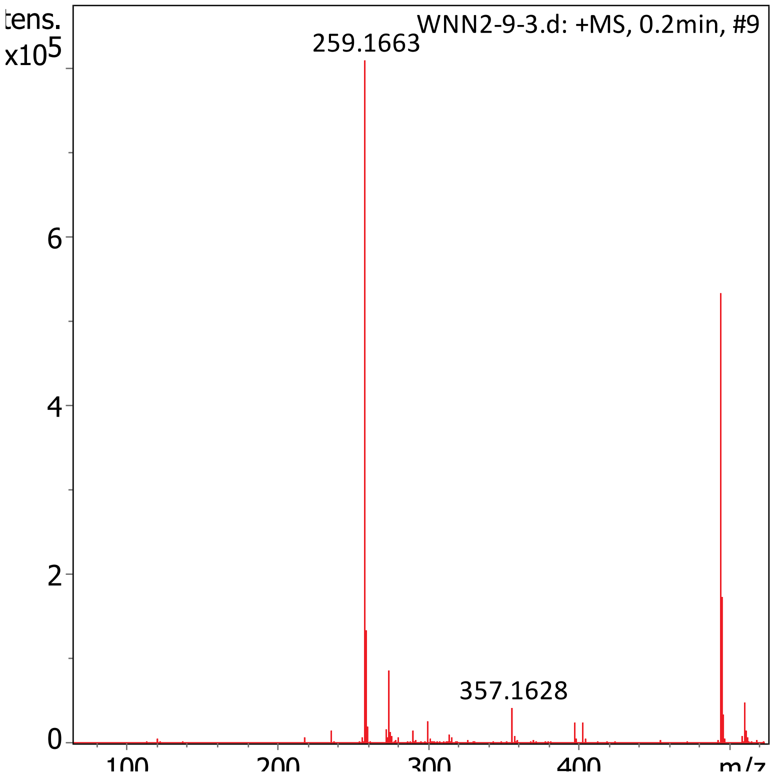

Figure S8. HRESIMS spectrum of compound **1**.

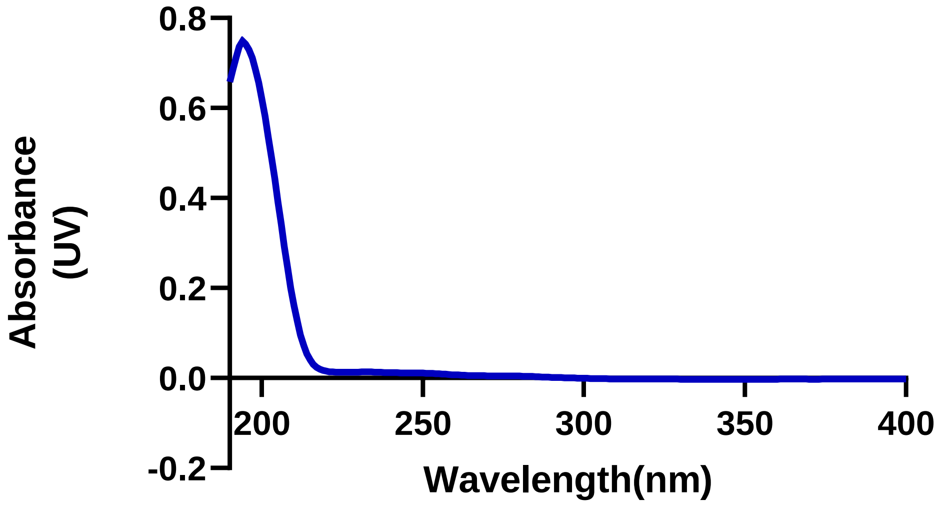

Figure S9. UV spectrum of compound **2** in MeCN.

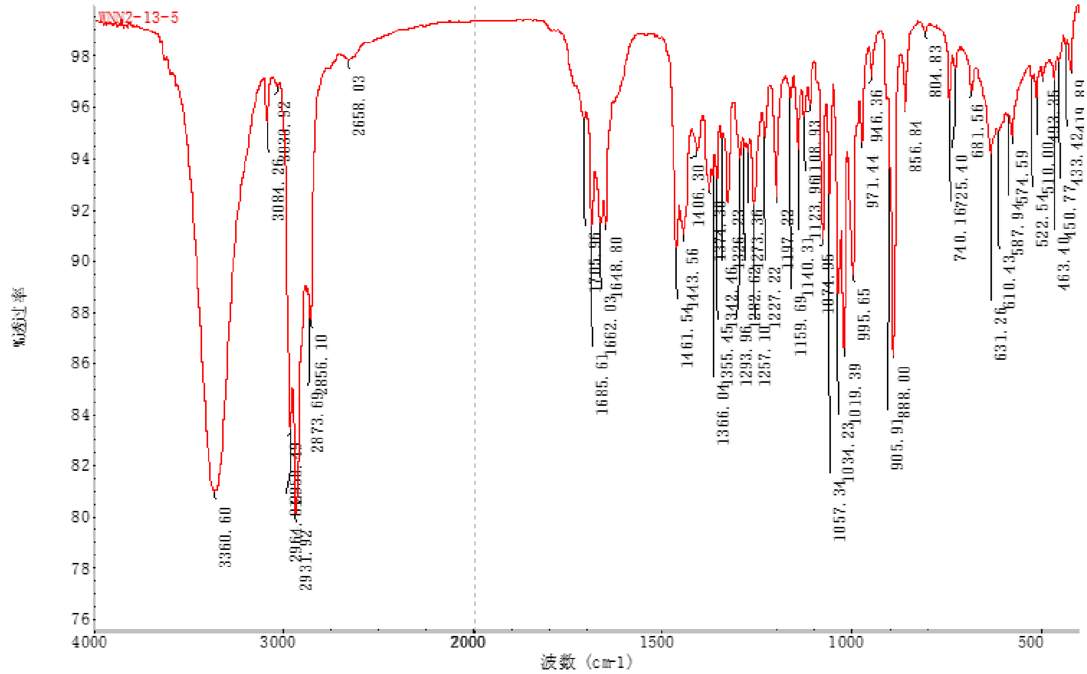

Figure S10. IR spectrum of compound **2**.

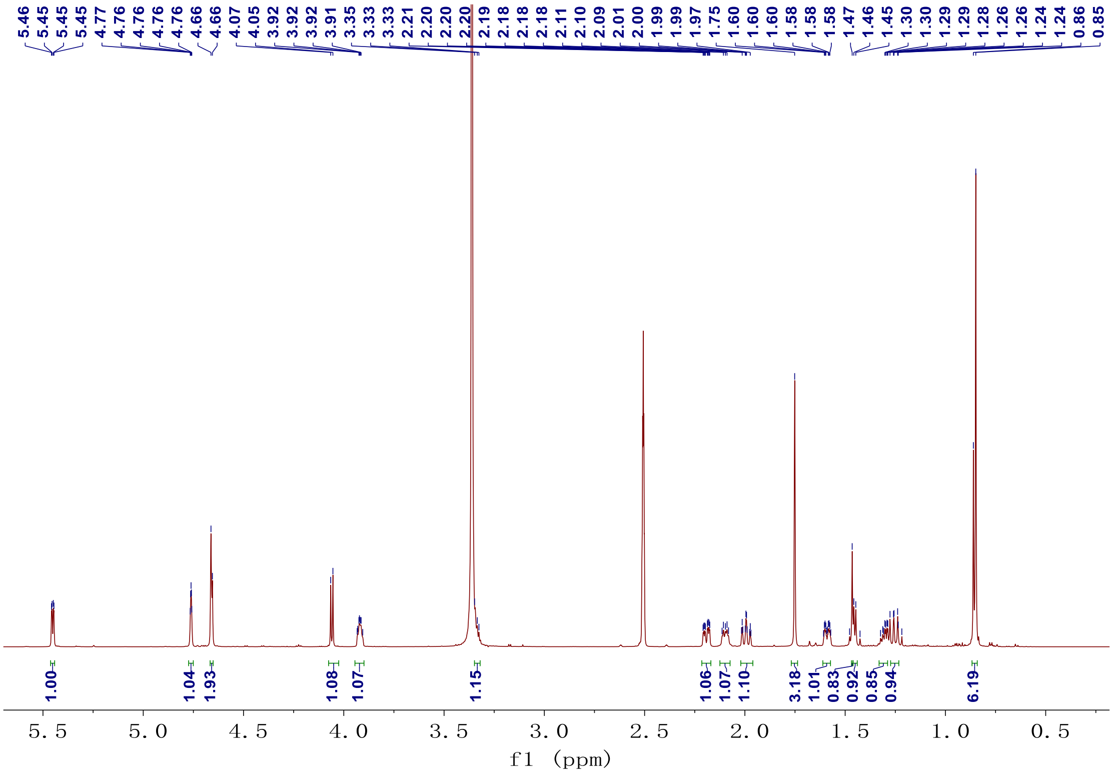

Figure S11. ^{1}H NMR spectrum of compound **2** (600 MHz, DMSO-d_6).

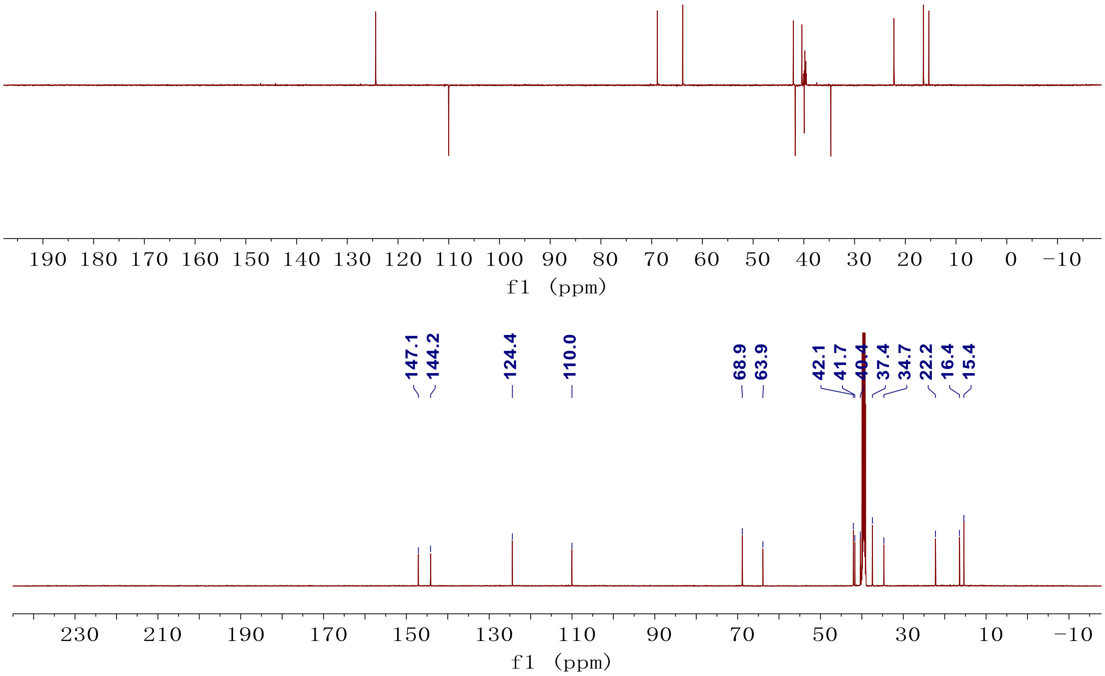

Figure S12. ^{13}C and DEPT-135 NMR spectra of compound **2** (150 MHz, DMSO-

*d*6).

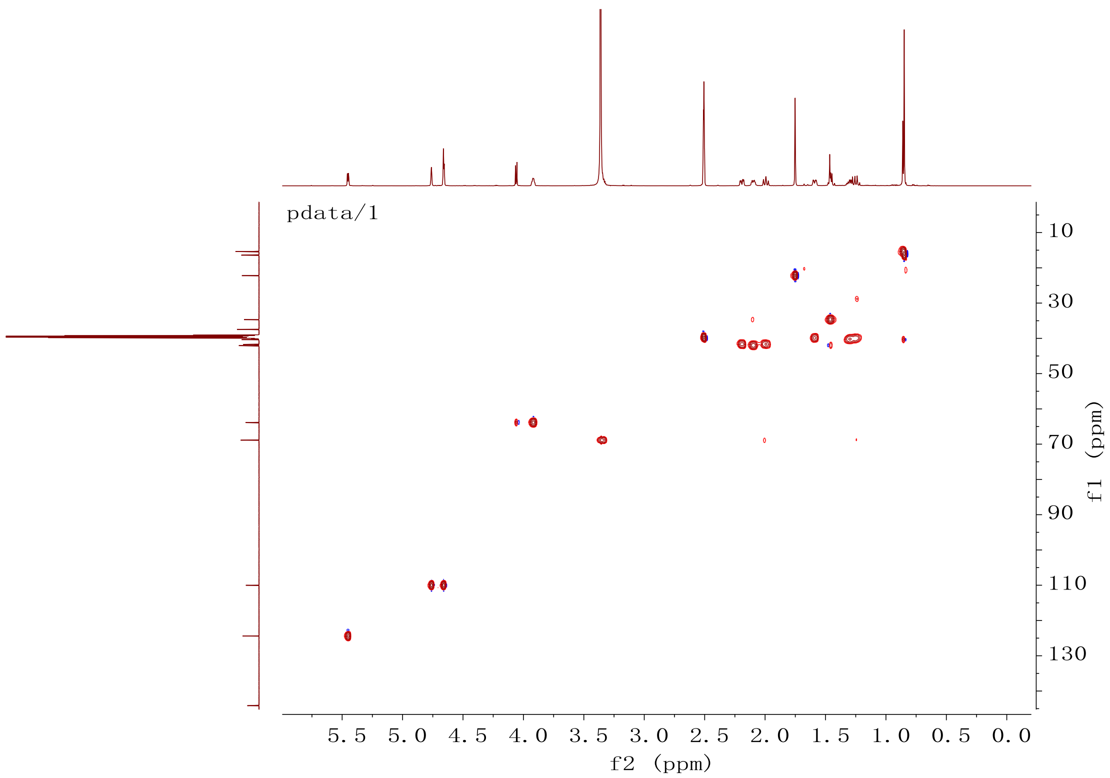

Figure S13. HSQC spectrum of compound **2** (DMSO-*d*6).

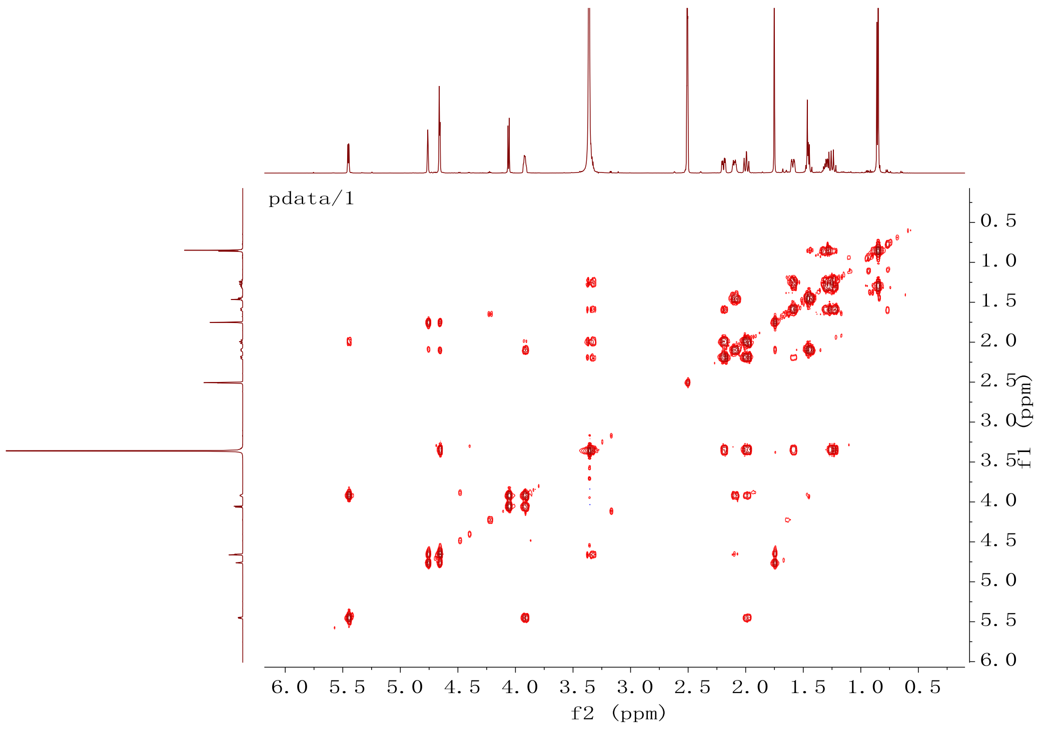

Figure S14. $^{1}H-^{1}H$ COSY spectrum of compound **2** (DMSO-*d*6).

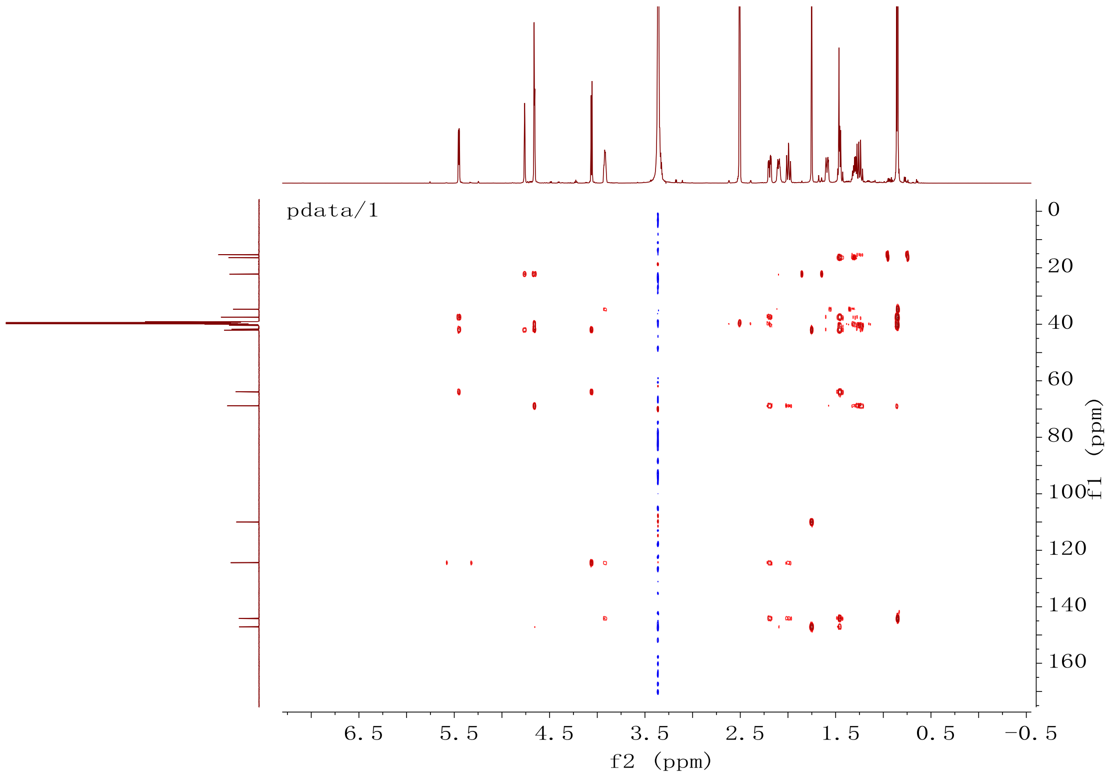

Figure S15. HMBC spectrum of compound **2** (DMSO-d_6).

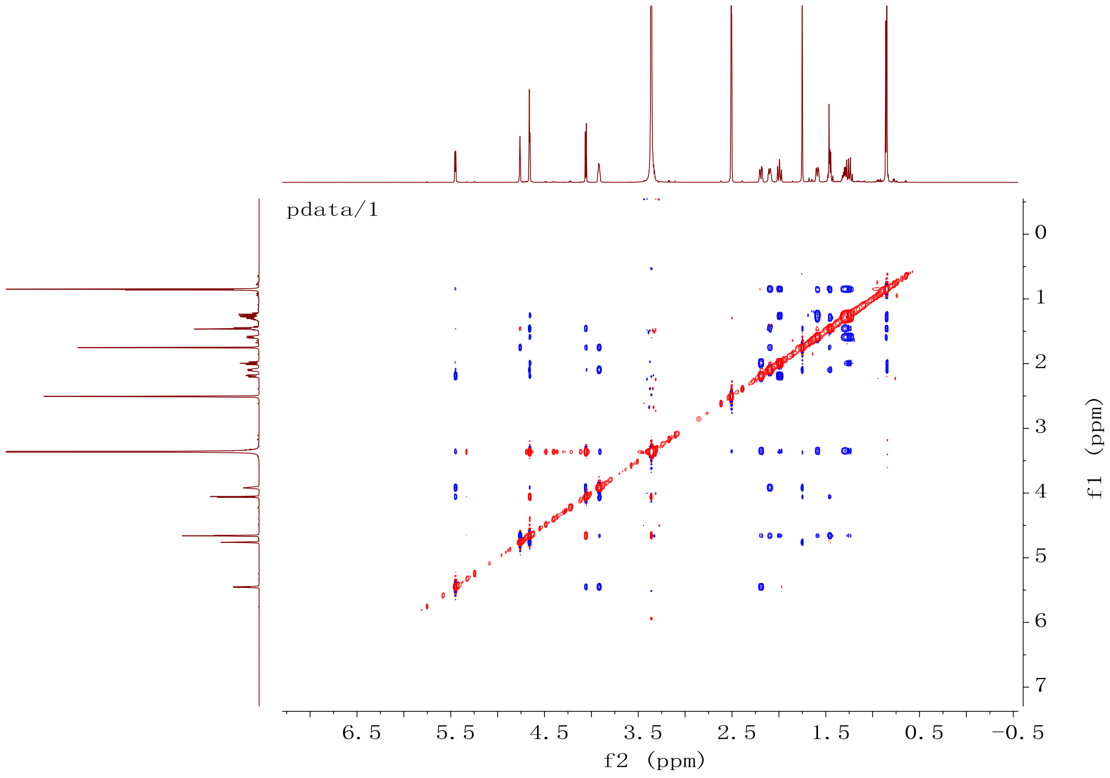

Figure S16. NOESY spectrum of compound **2** (DMSO-d_6).

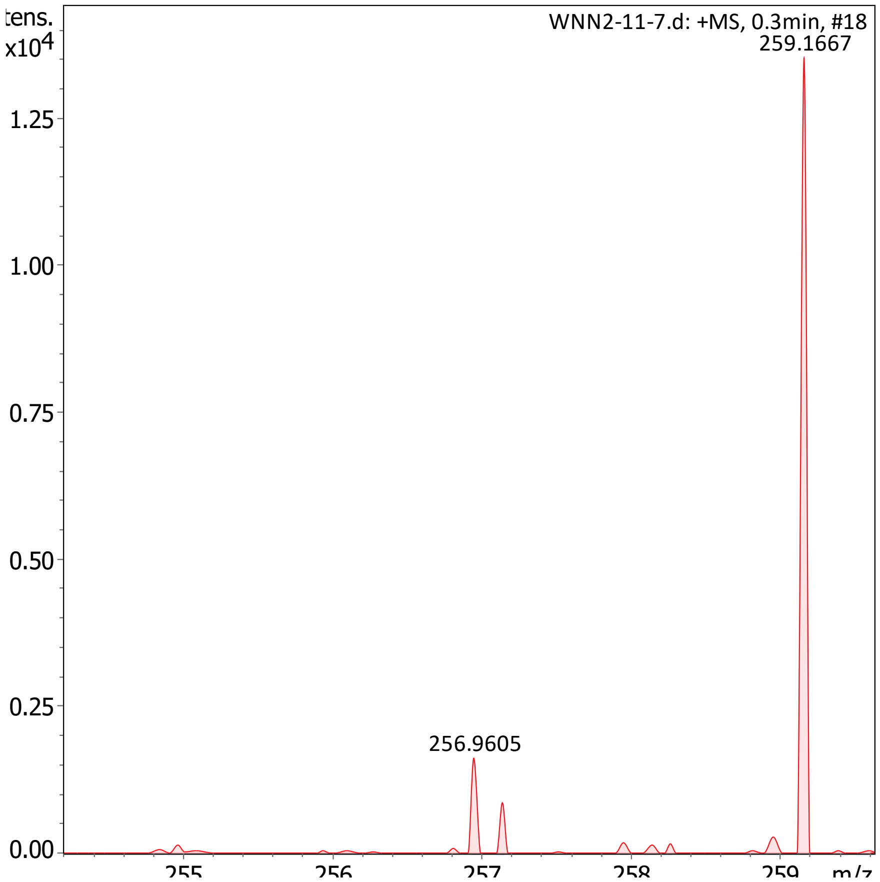

Figure S17. HRESIMS spectrum of compound **2**.

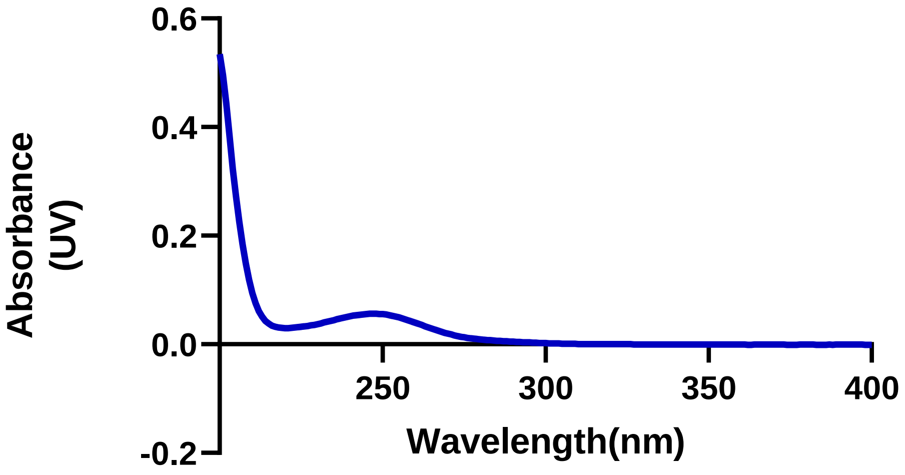

Figure S18. UV spectrum of compound **3** in MeCN.

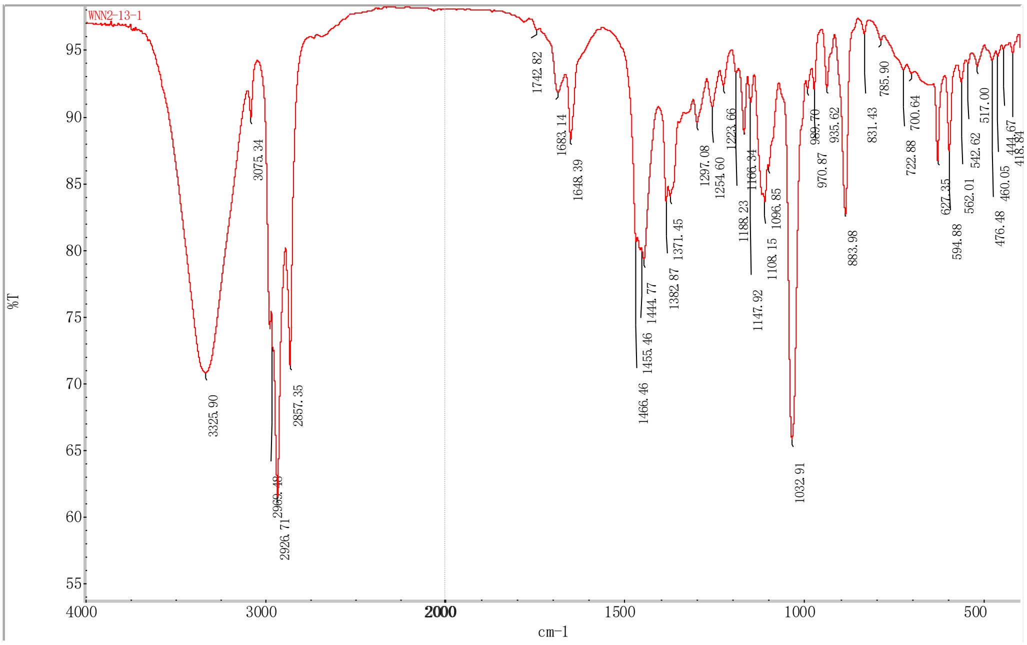

Figure S19. IR spectrum of compound **3**.

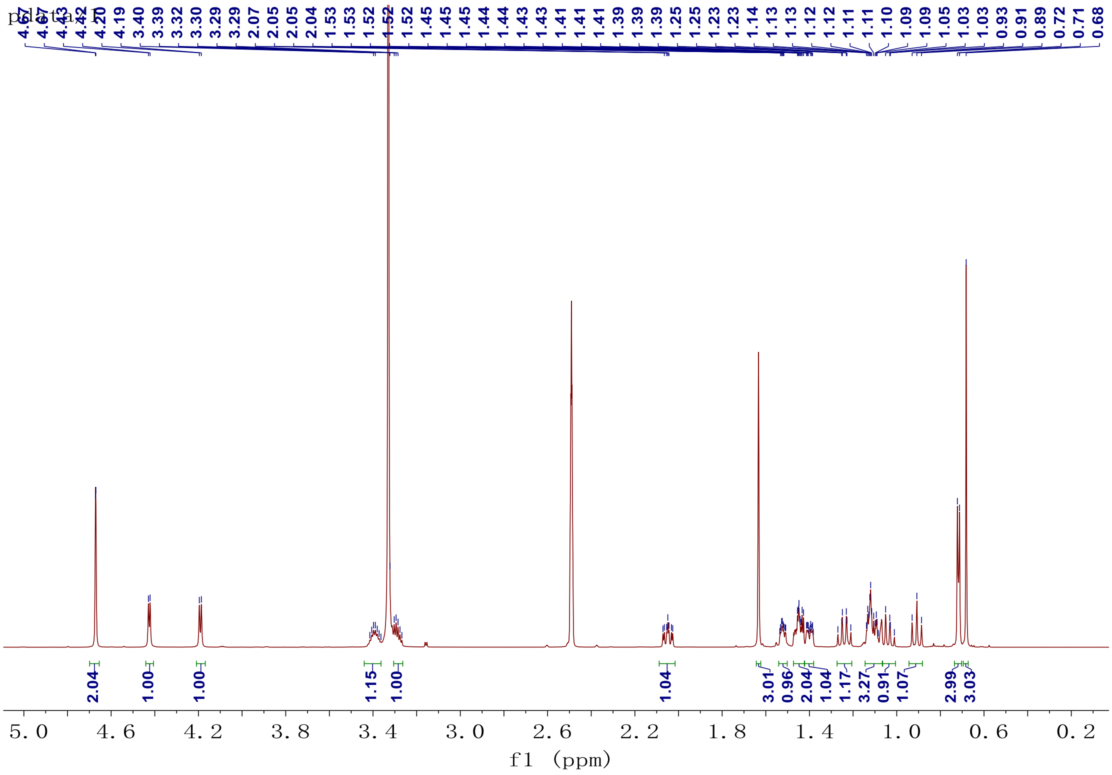

Figure S20. ^{1}H NMR spectrum of compound **3** (600 MHz, DMSO-d_6).

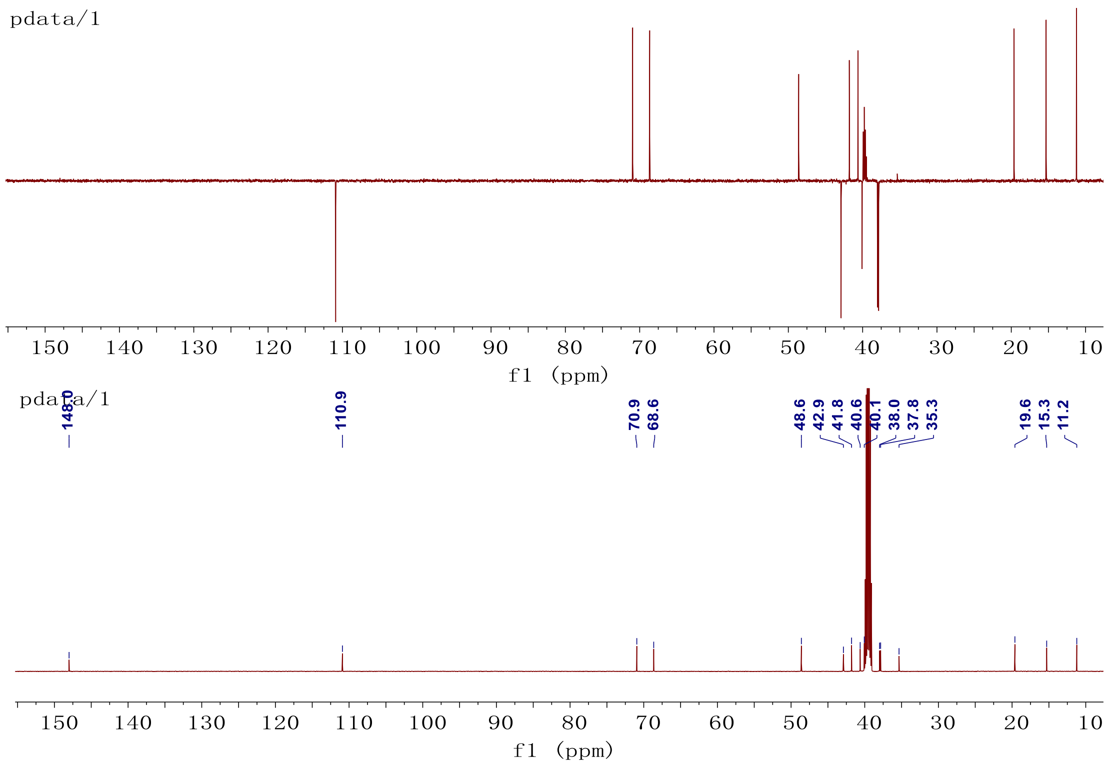

Figure S21. ^{13}C and DEPT-135 NMR spectra of compound **3** (150 MHz, DMSO-*d*$_6$).

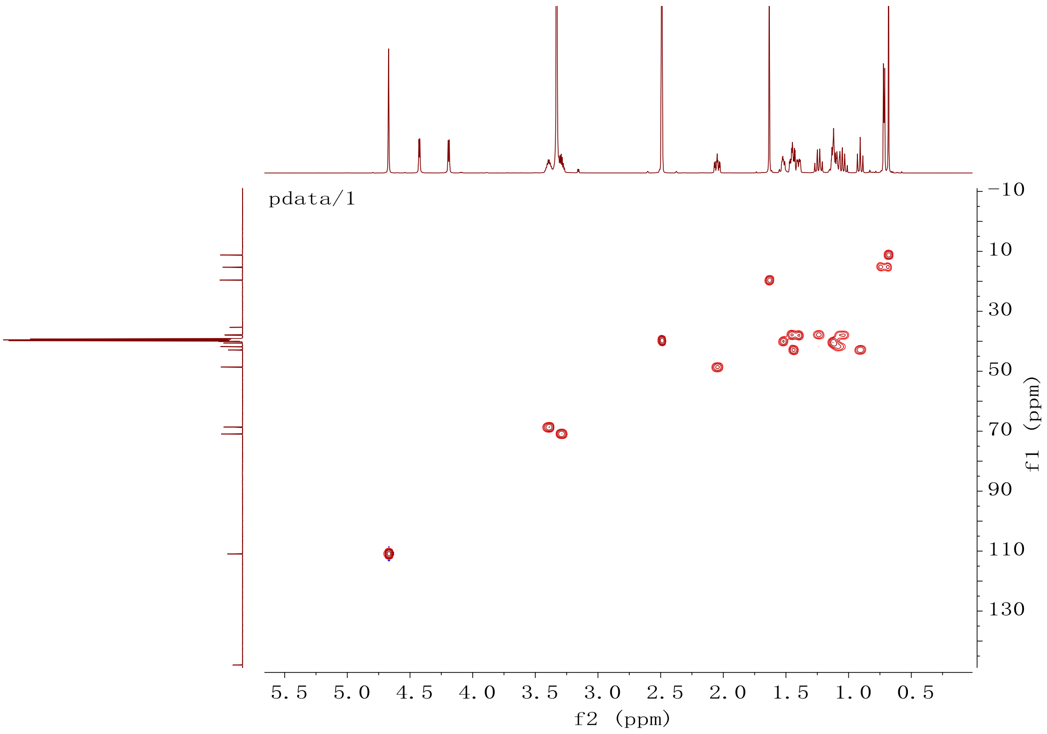

Figure S22. HSQC spectrum of compound **3** (DMSO-*d*$_6$).

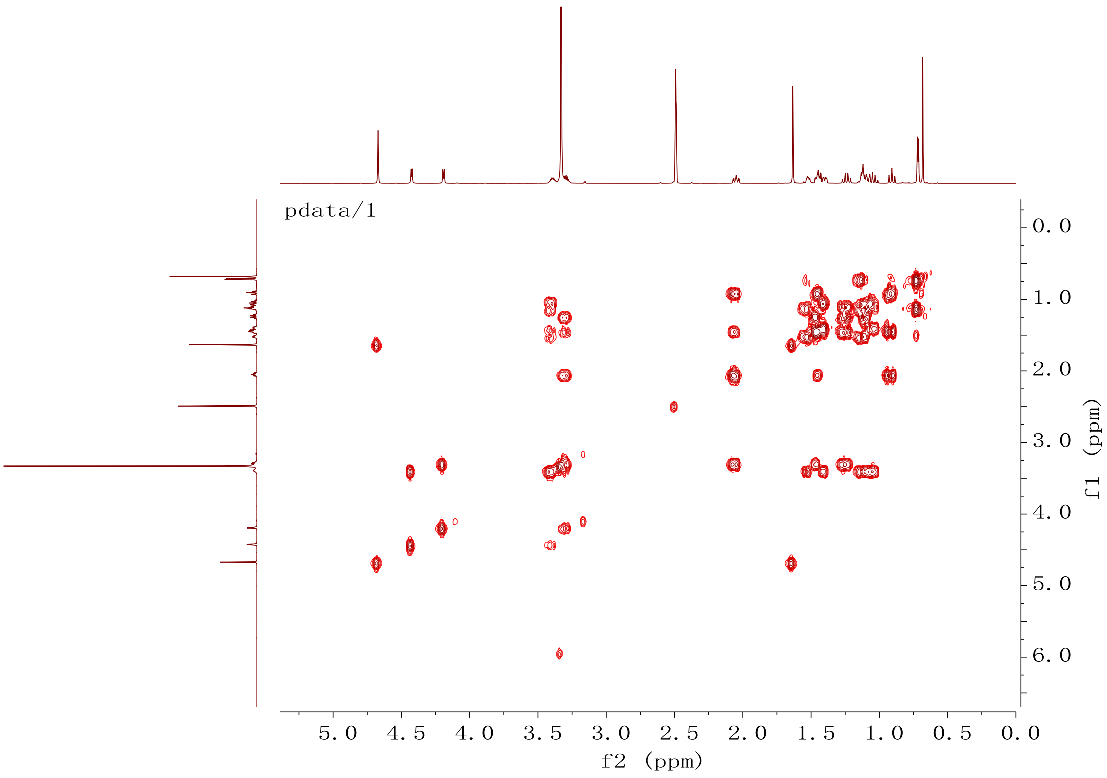

Figure S23. $^1H-^1H$ COSY spectrum of compound **3** (DMSO-d_6).

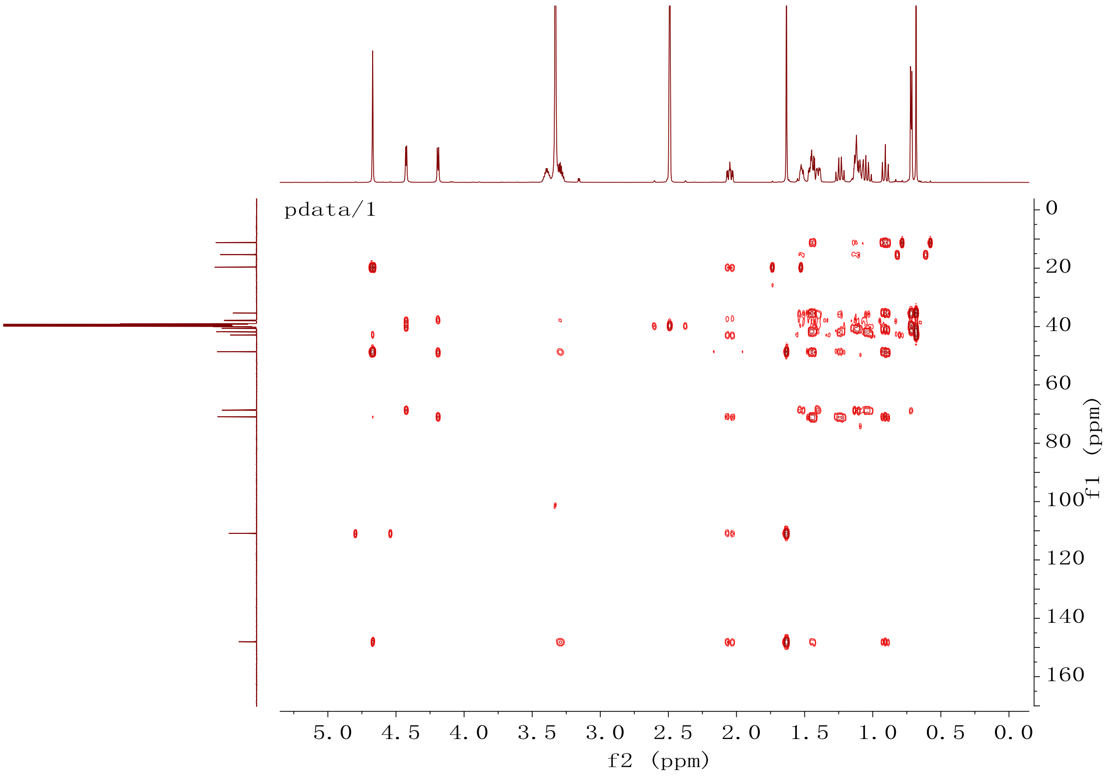

Figure S24. HMBC spectrum of compound **3** (DMSO-d_6).

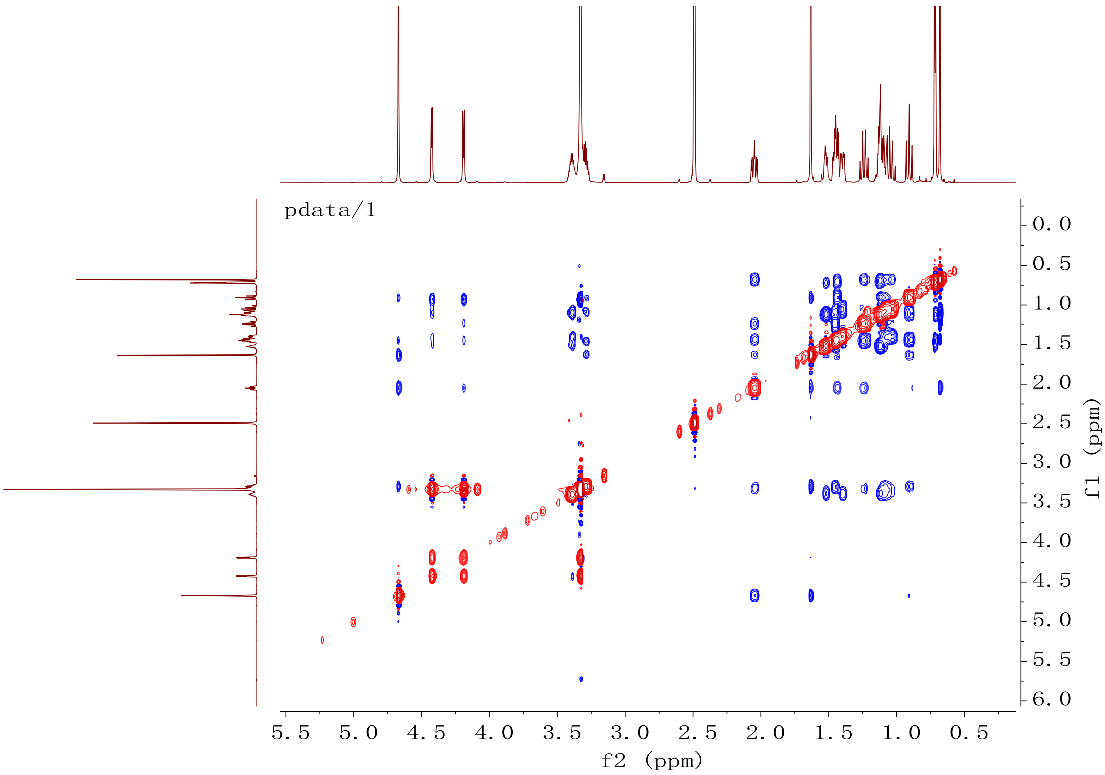

Figure S25. NOESY spectrum of compound **3** (DMSO-d_6).

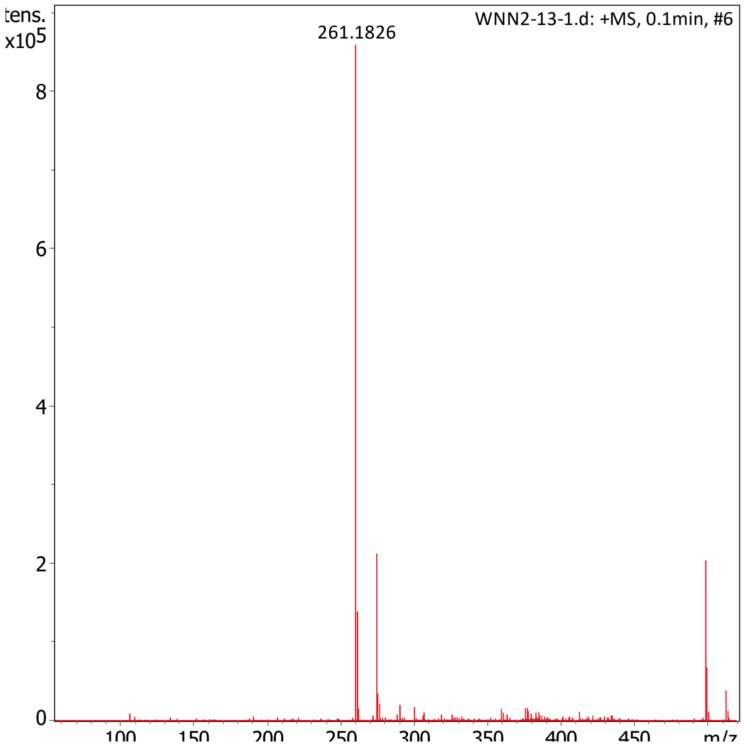

Figure S26. HRESIMS spectrum of compound **3**.

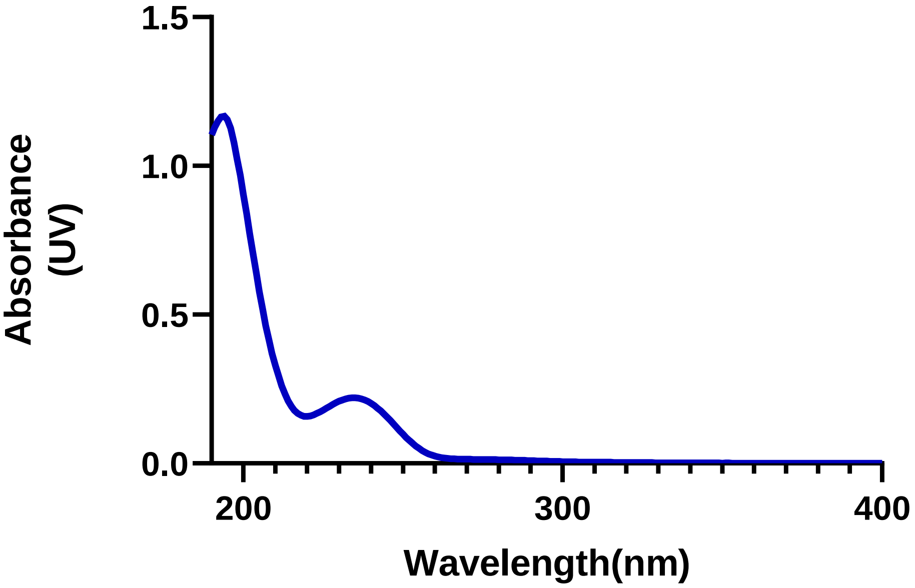

Figure S27. UV spectrum of compound **4** in MeCN.

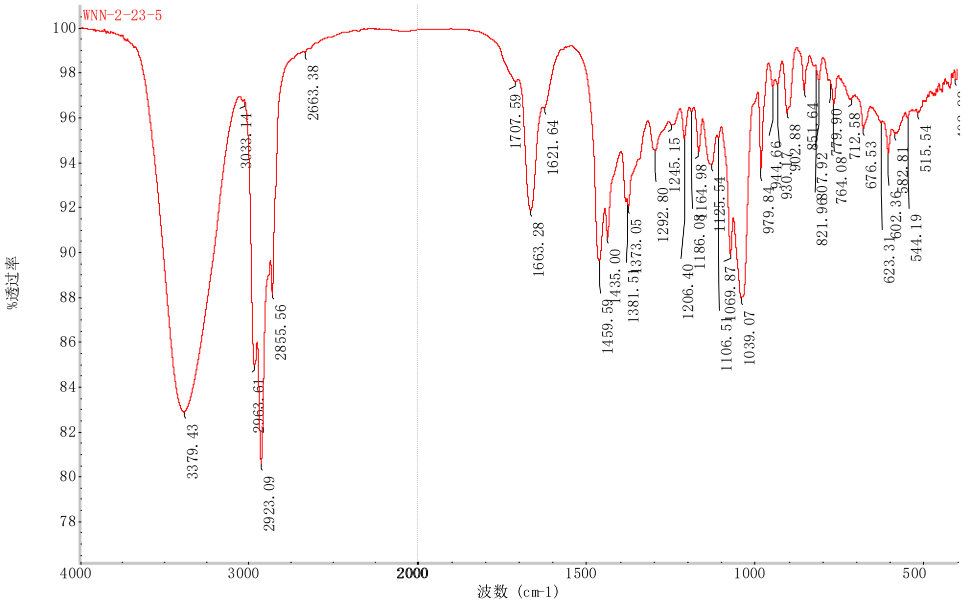

Figure S28. IR spectrum of compound **4**.

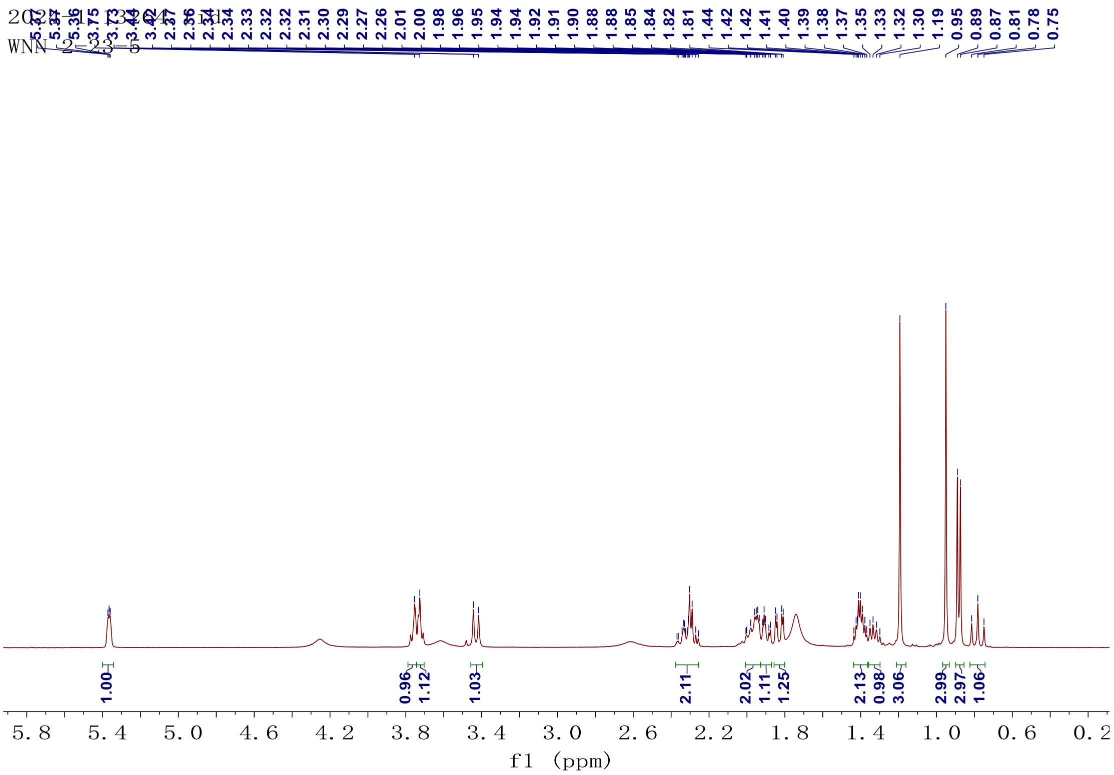

Figure S29. ^{1}H NMR spectrum of compound **4** (400 MHz, $CDCl_3$).

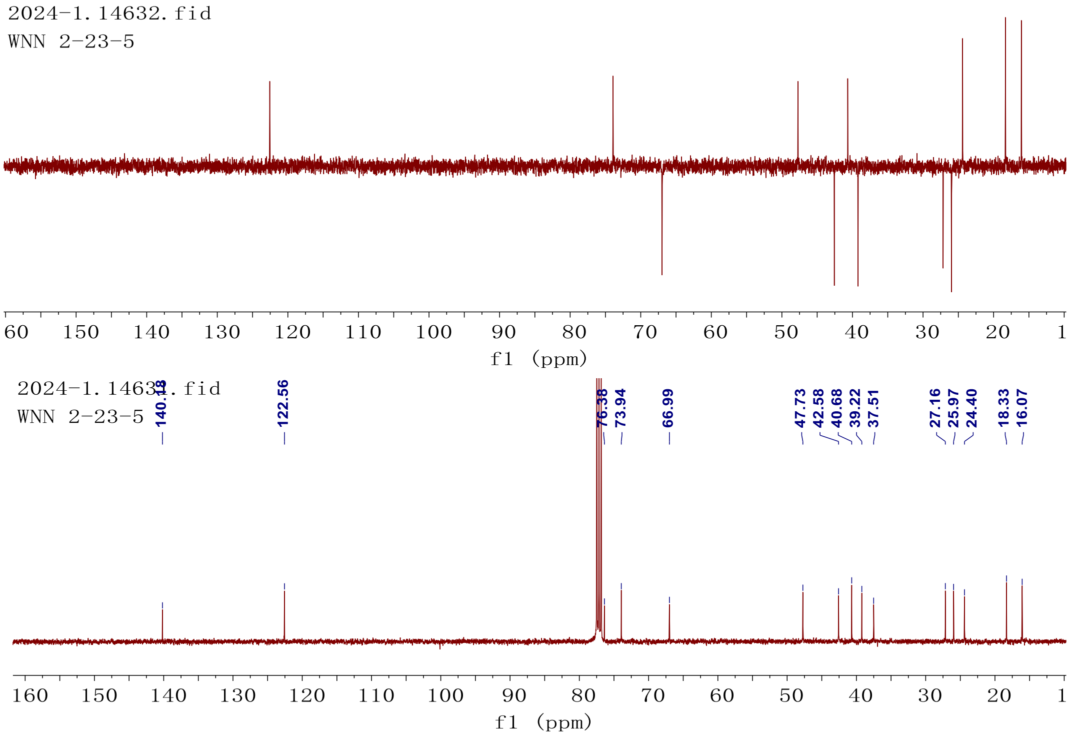

Figure S30. ^{13}C and DEPT-135 NMR spectra of compound **4** (100 MHz, $CDCl_3$).

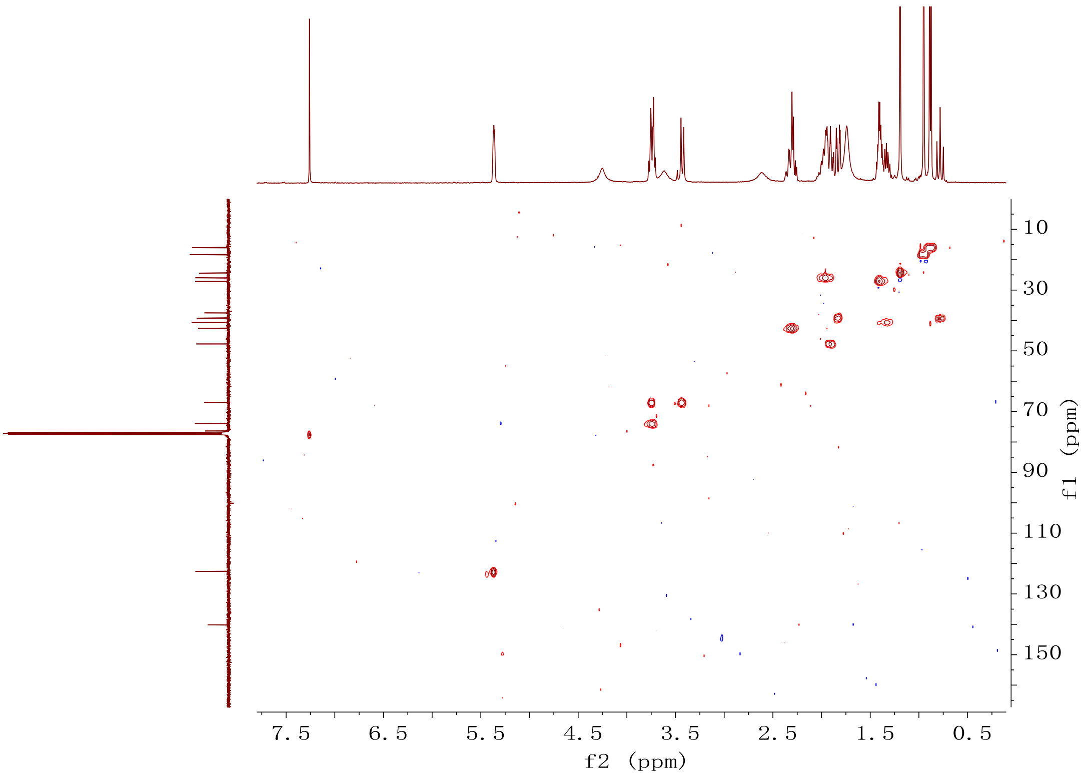

Figure S31. HSQC spectrum of compound **4** ($CDCl_3$).

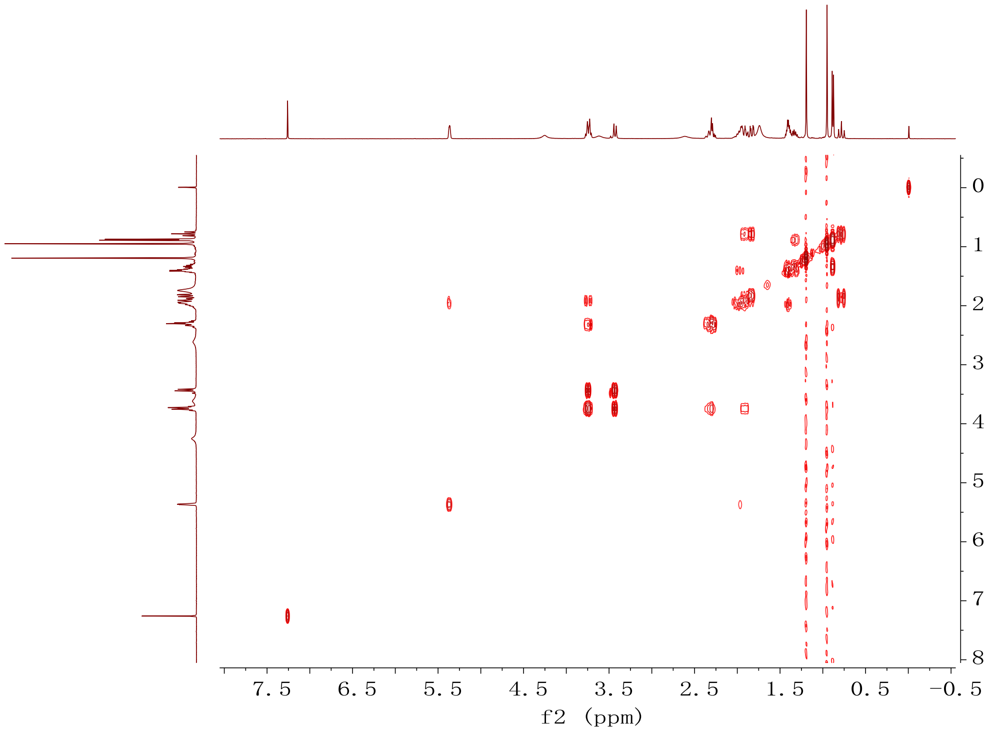

Figure S32. ^1H–^1H COSY spectrum of compound **4** ($CDCl_3$).

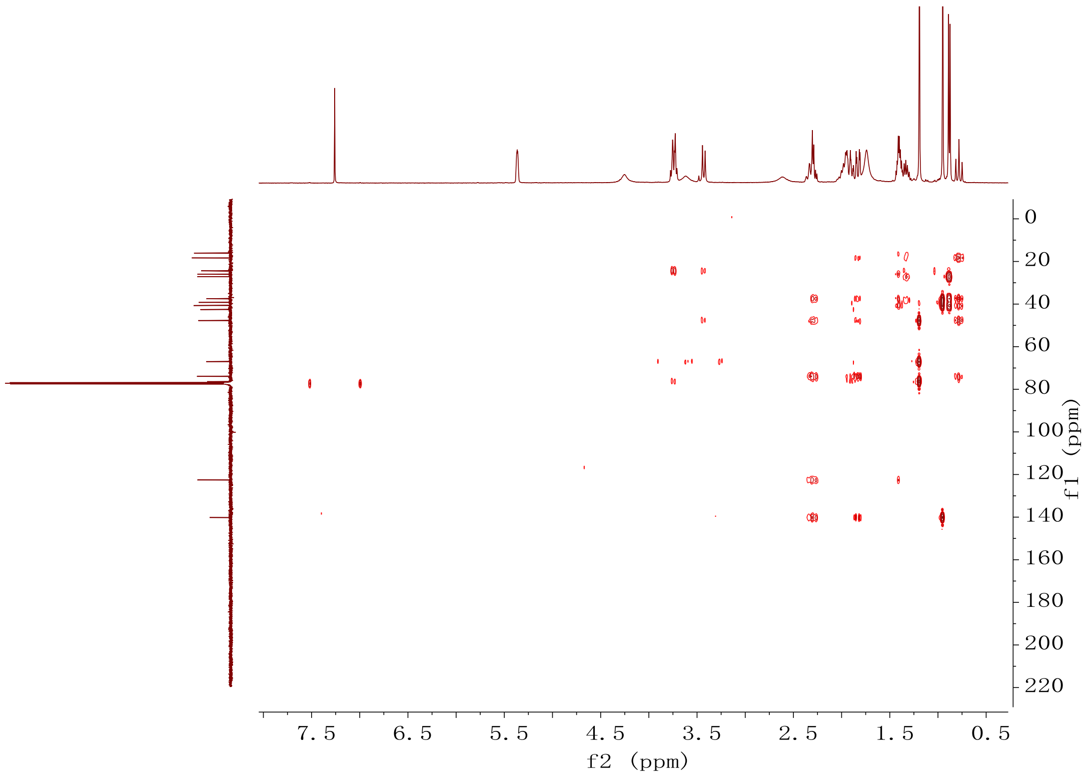

Figure S33. HMBC spectrum of compound **4** ($CDCl_3$).

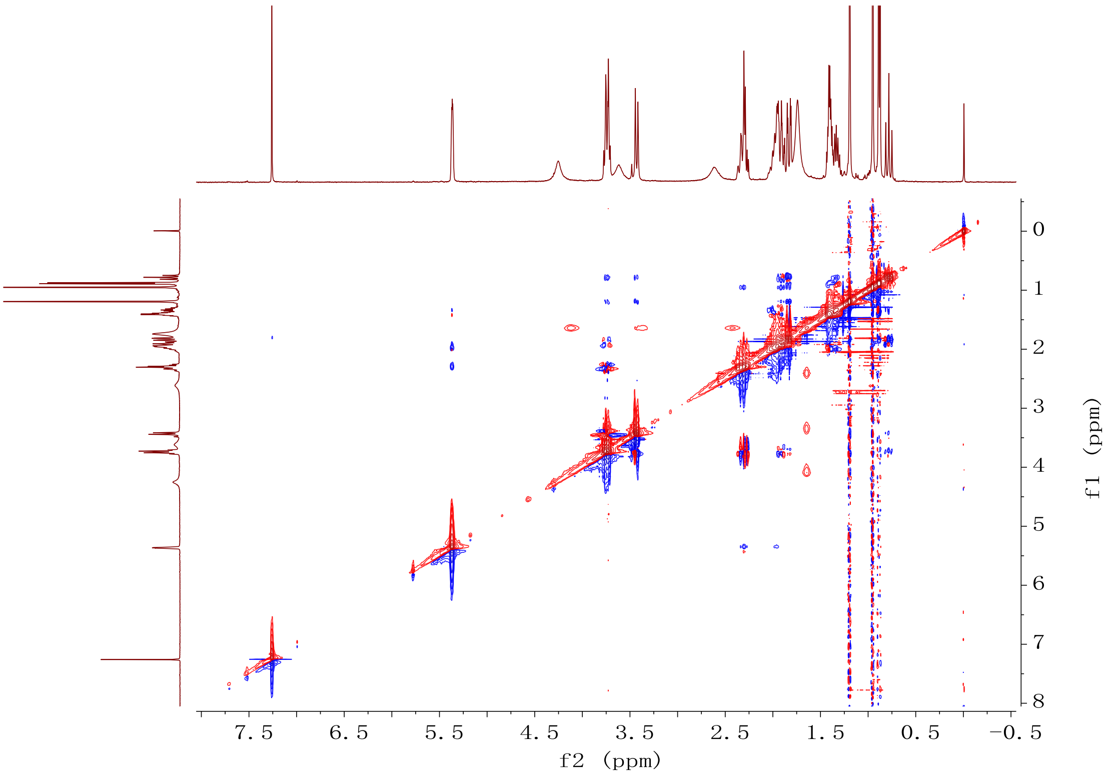

Figure S34. NOESY spectrum of compound **4** ($CDCl_3$).

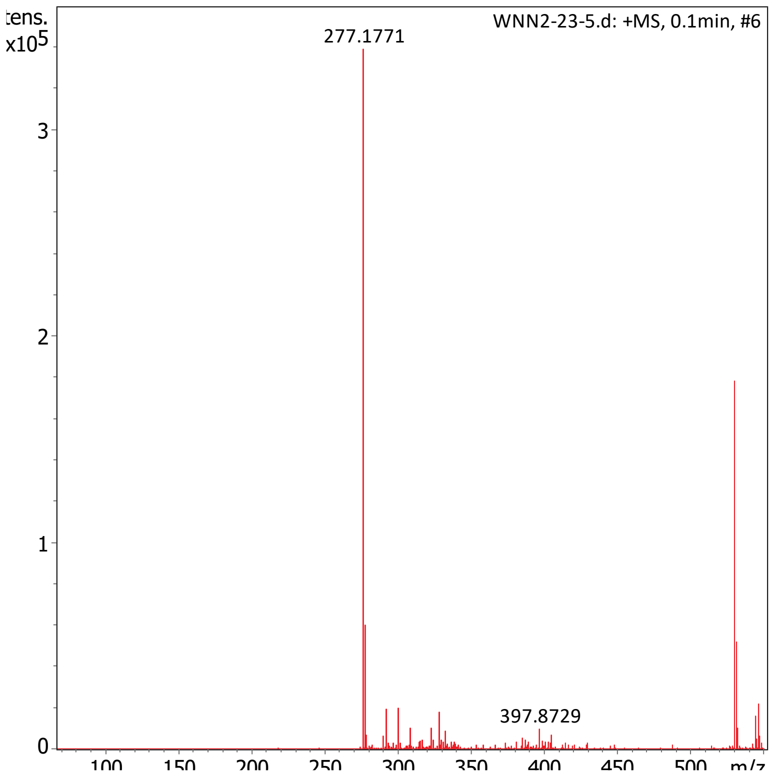

Figure S35. HRESIMS spectrum of compound **4**.

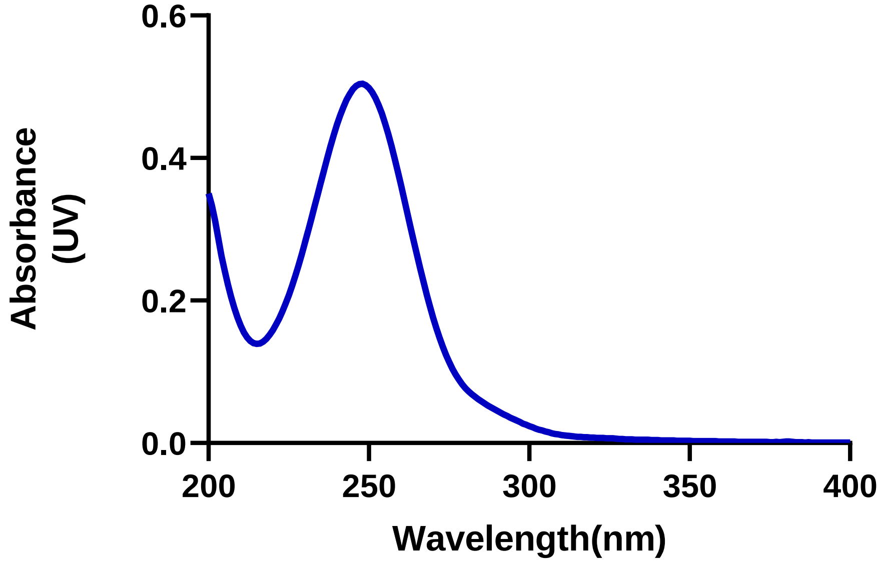

Figure S36. UV spectrum of compound **5** in MeCN.

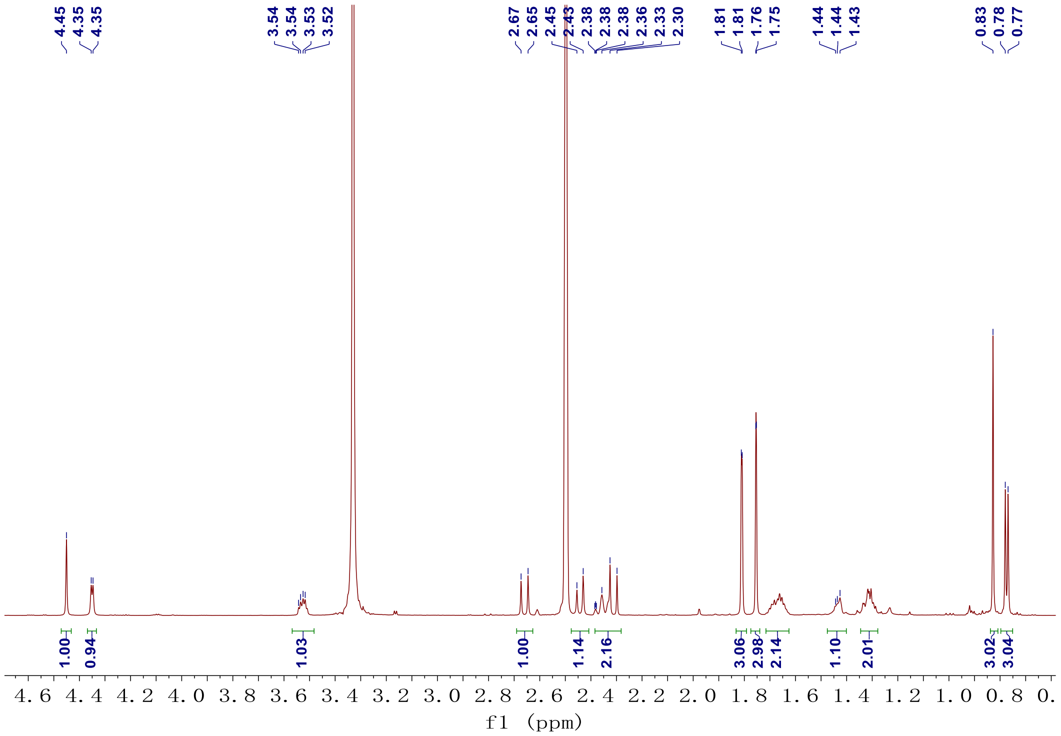

Figure S37. ^{1}H NMR spectrum of compound **5** (600 MHz, DMSO-d_6).

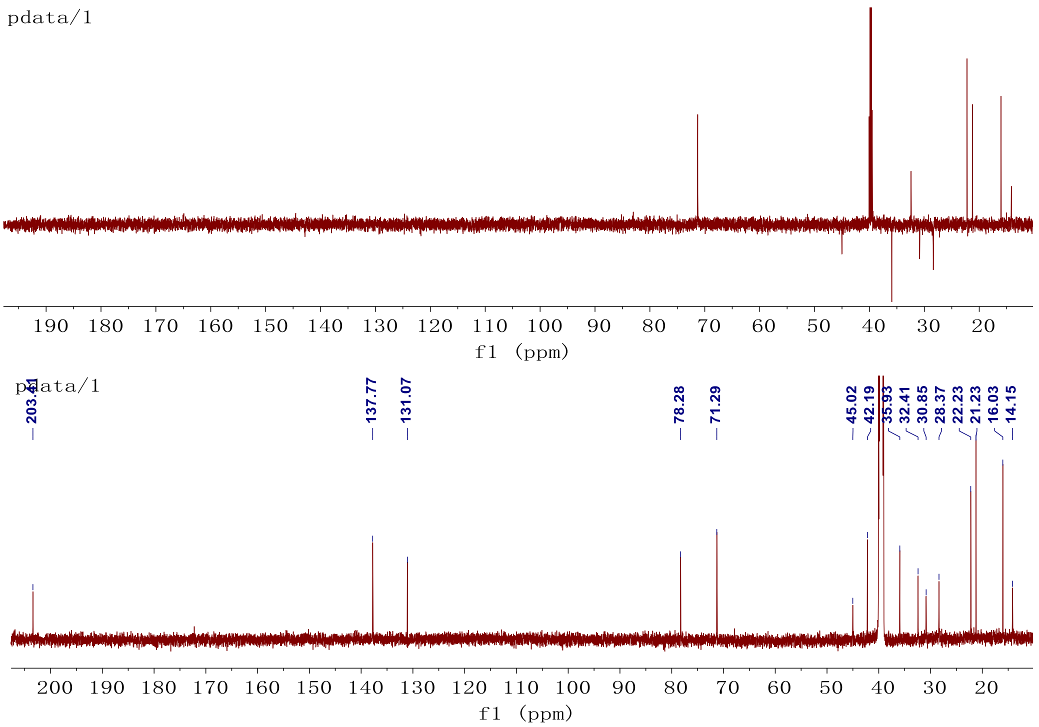

Figure S38. ^{13}C and DEPT-135 NMR spectra of compound **5** (150 MHz, DMSO-d_6).

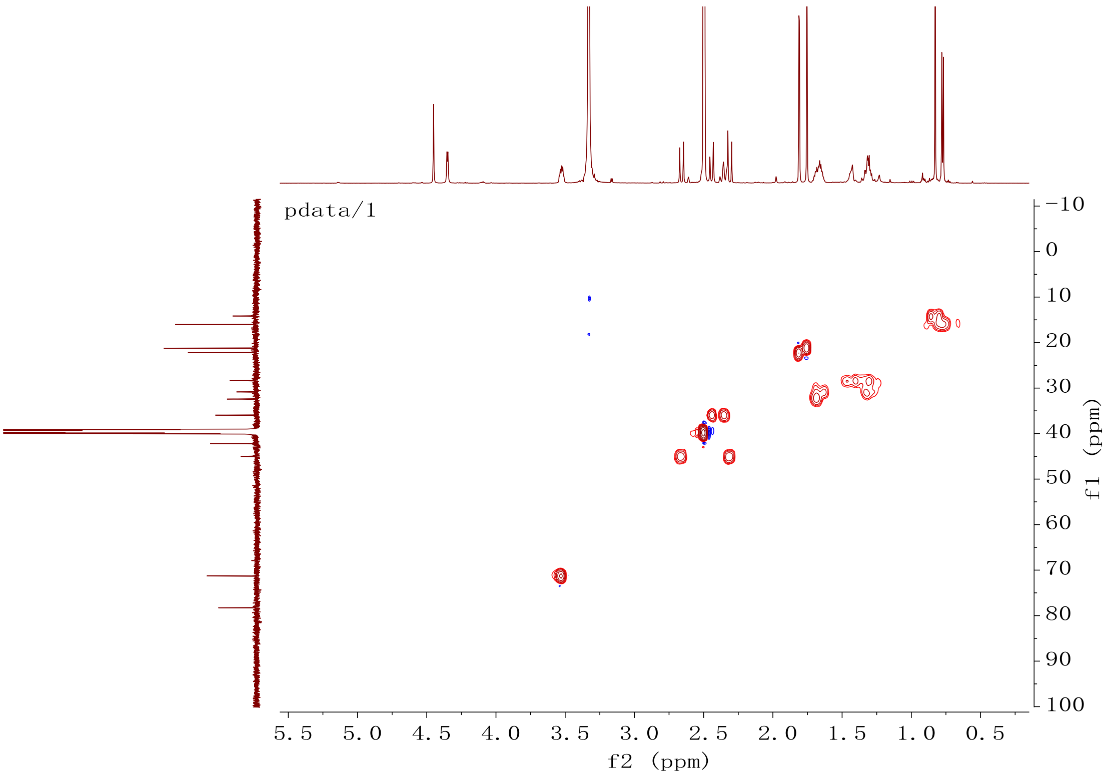

Figure S39. HSQC spectrum of compound **5** (DMSO-d_6).

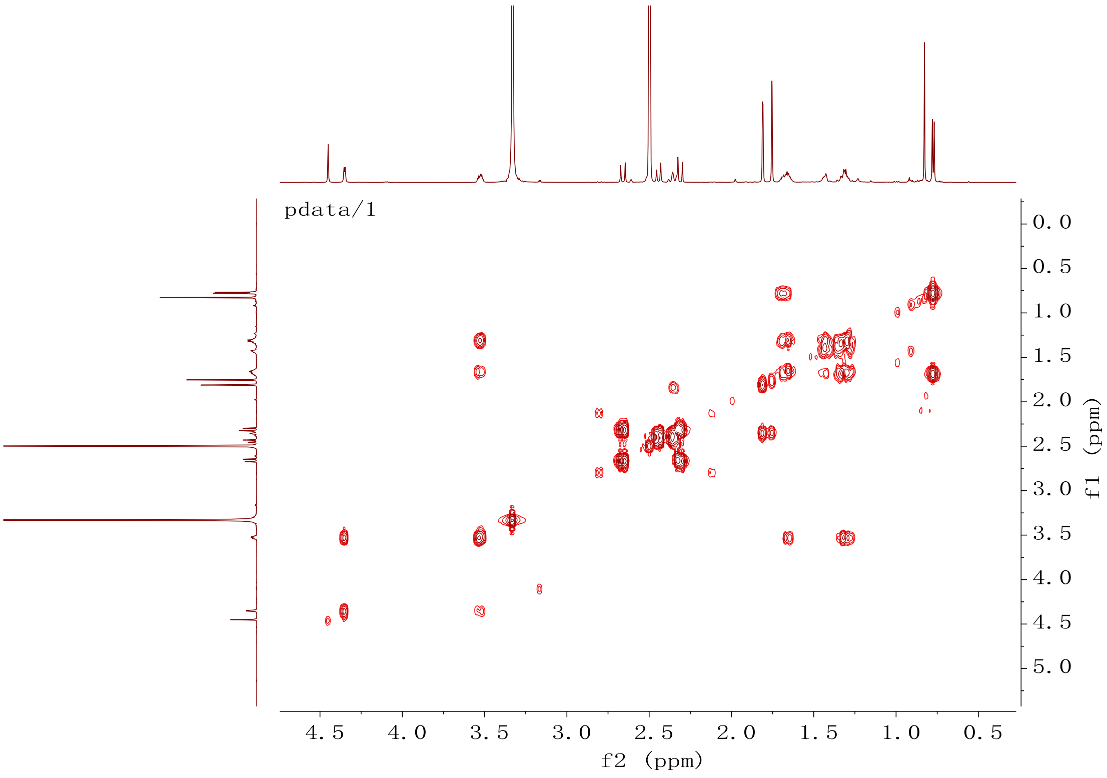

Figure S40. $^1H–^1H$ COSY spectrum of compound **5** (DMSO-d_6).

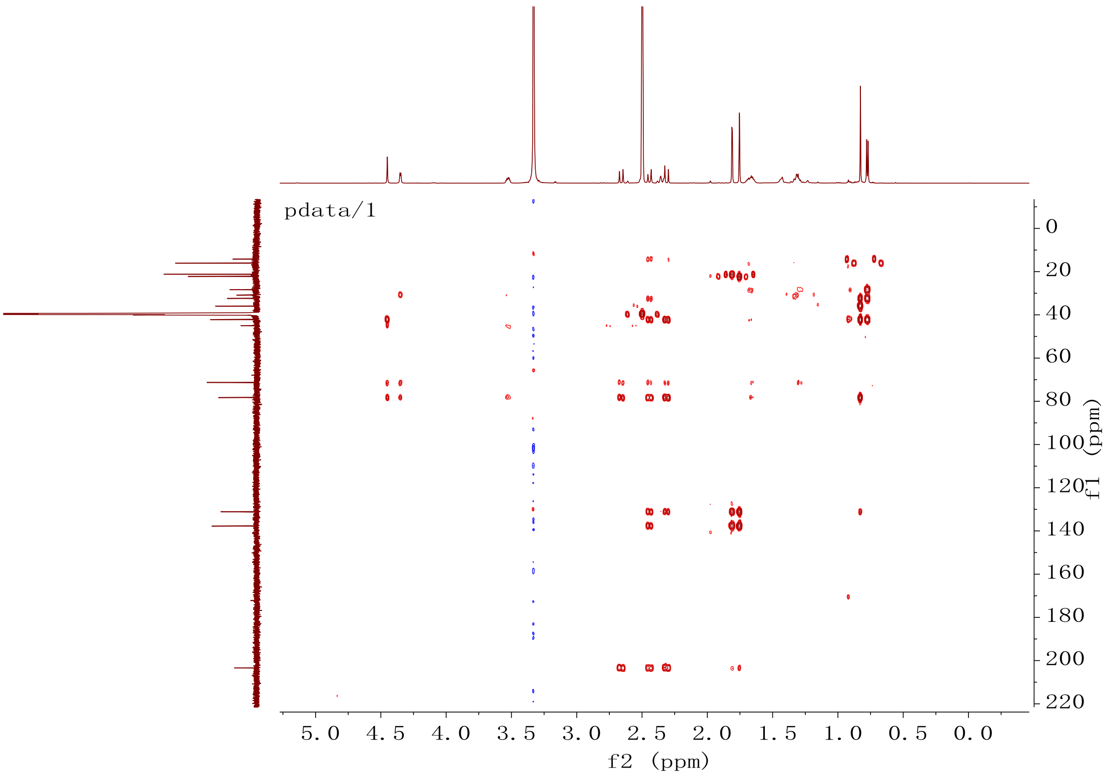

Figure S41. HMBC spectrum of compound **5** (DMSO-d_6).

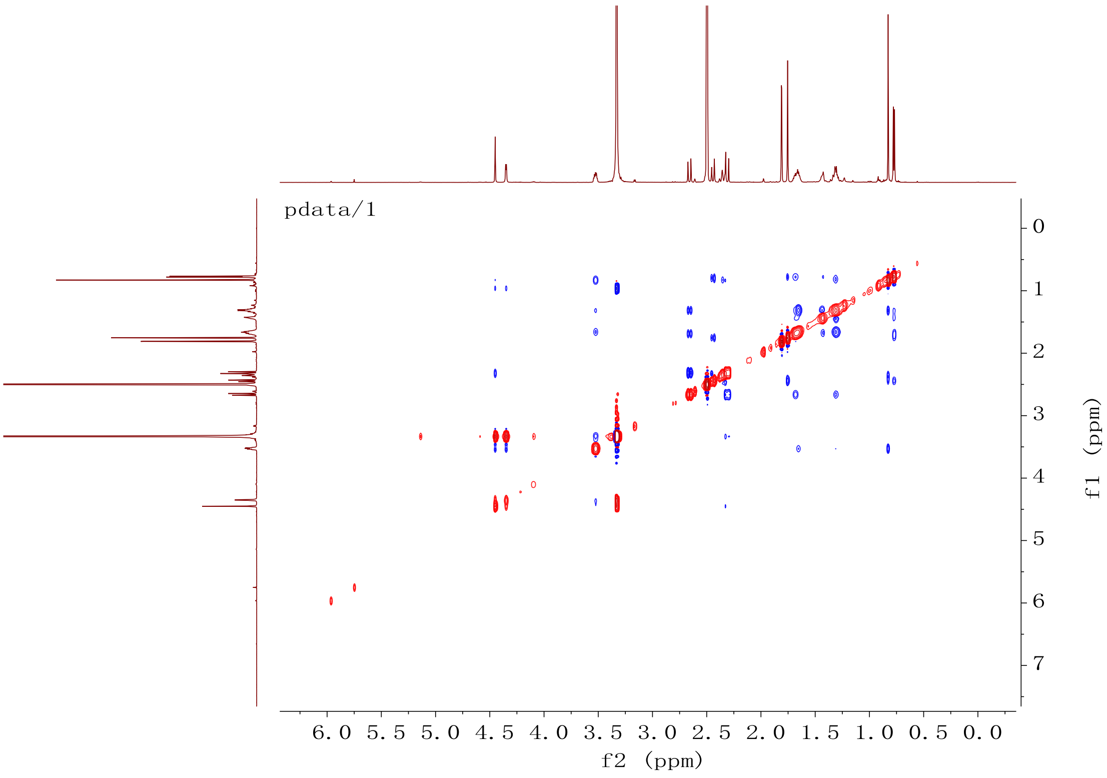

Figure S42. NOESY spectrum of compound **5** (DMSO-d_6).

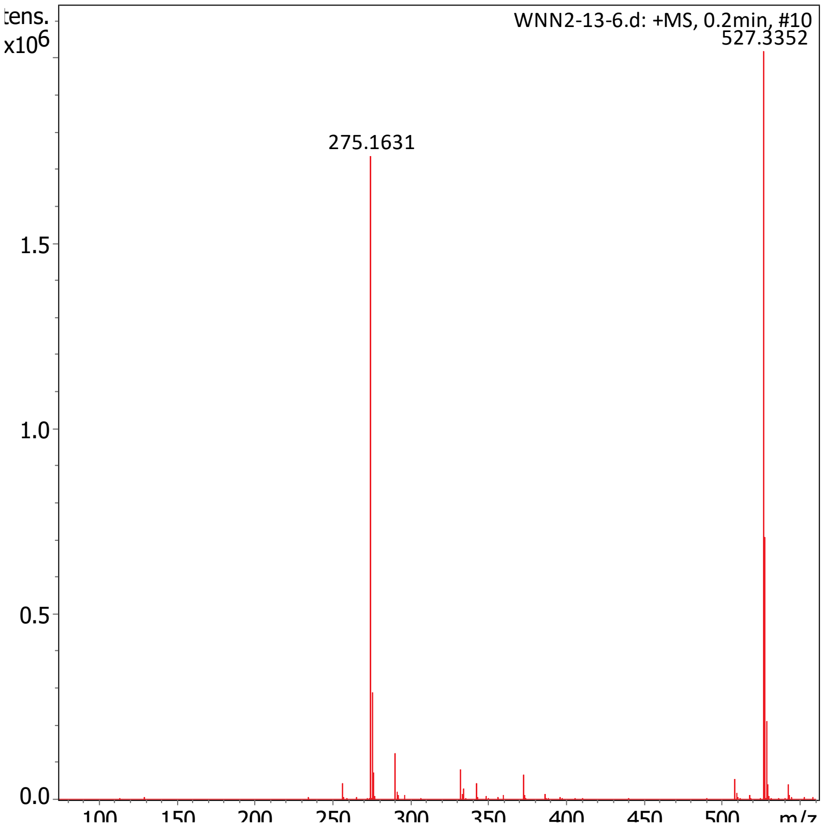

Figure S43. HRESIMS spectrum of compound **5**.

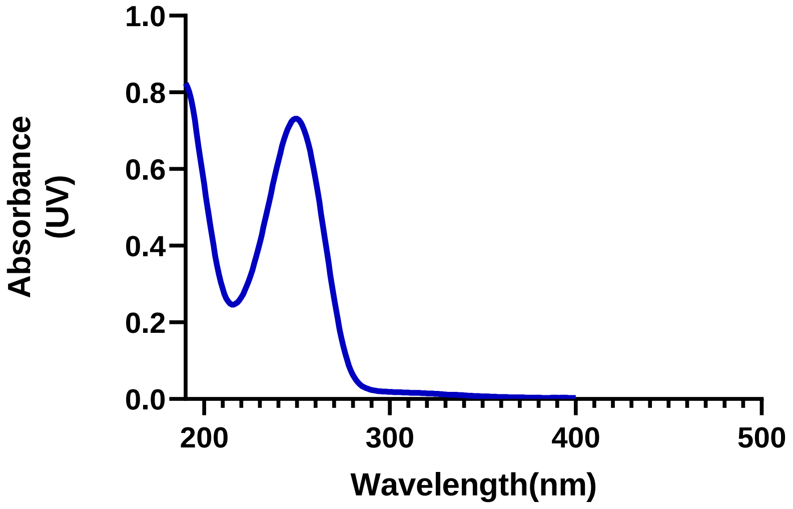

Figure S44. UV spectrum of compound **6** in MeCN.

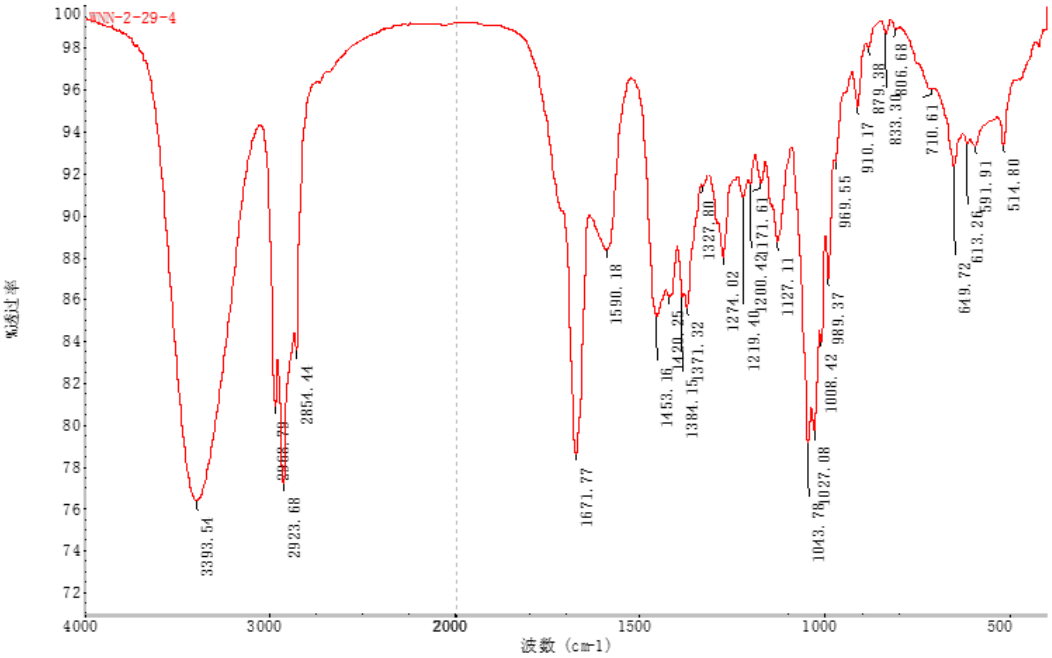

Figure S45. IR spectrum of compound **6**.

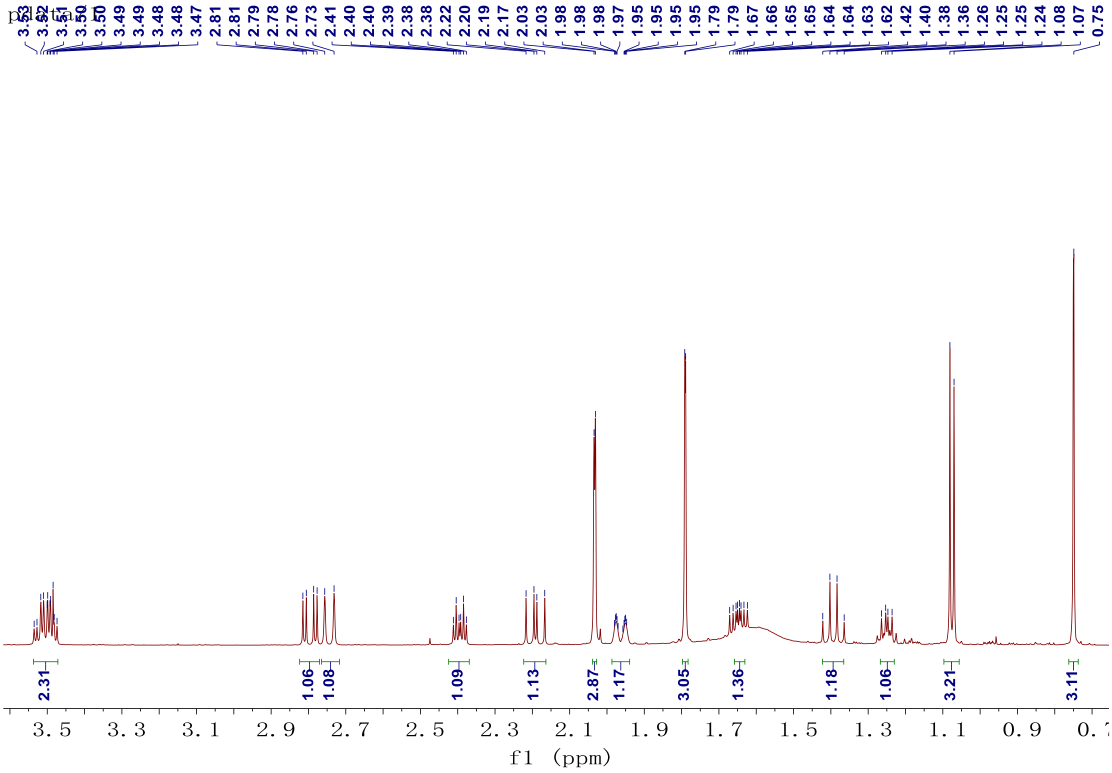

Figure S46. ^{1}H NMR spectrum of compound **6** (600 MHz, $CDCl_3$).

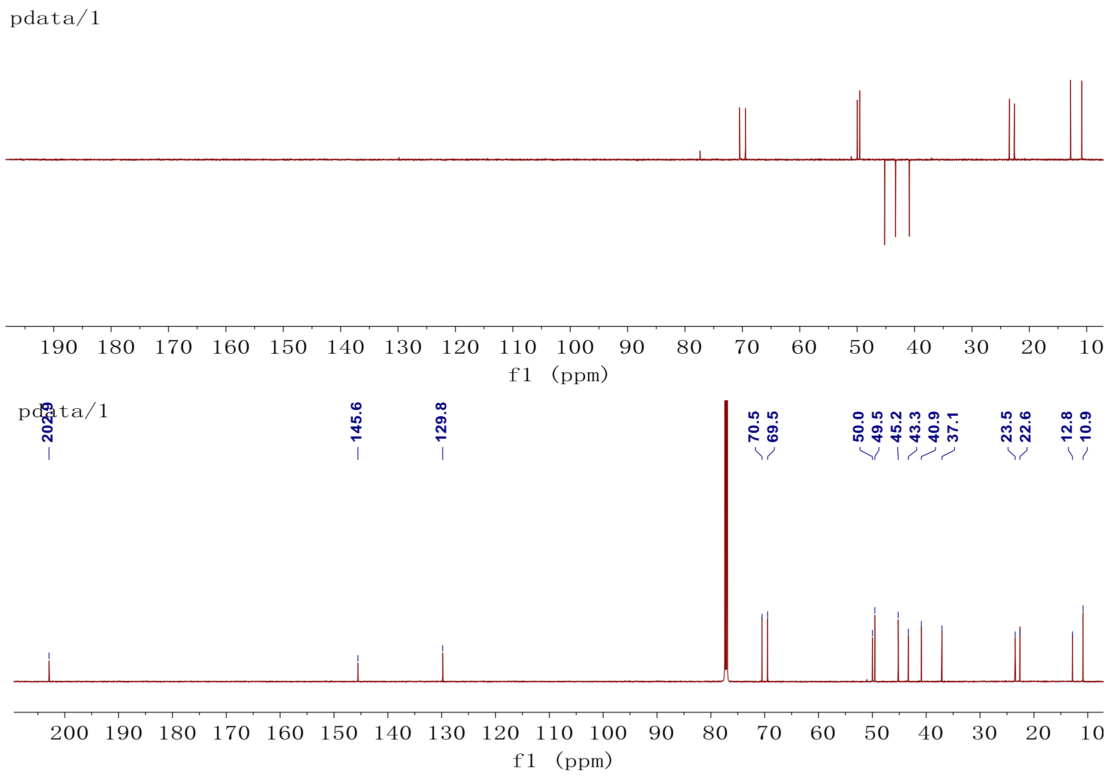

Figure S47. ^{13}C and DEPT-135 NMR spectra of compound **6** (150 MHz, $CDCl_3$).

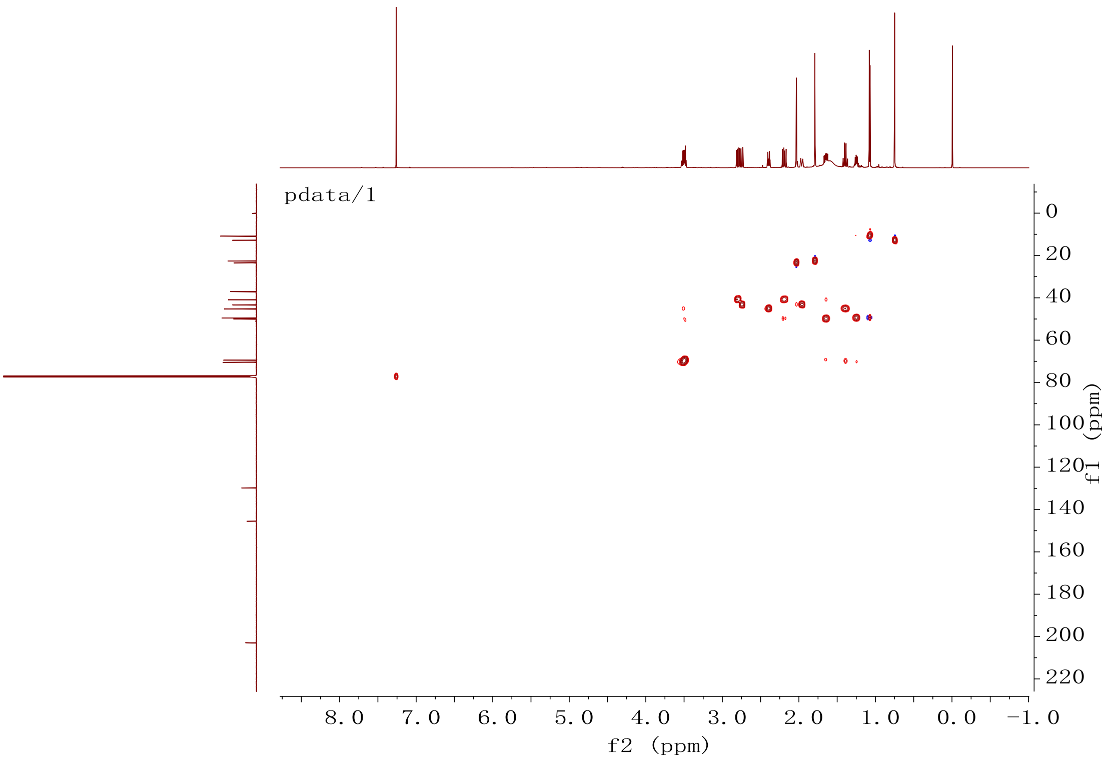

Figure S48. HSQC spectrum of compound **6** ($CDCl_3$).

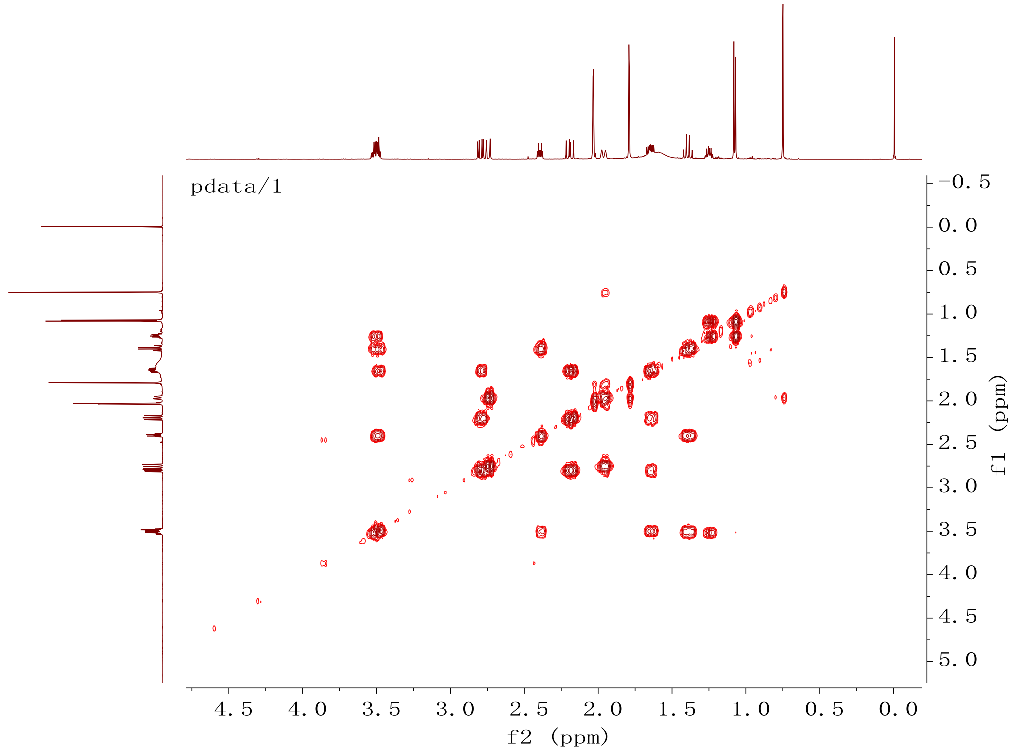

Figure S49. ^{1}H–^{1}H COSY spectrum of compound **6** ($CDCl_3$).

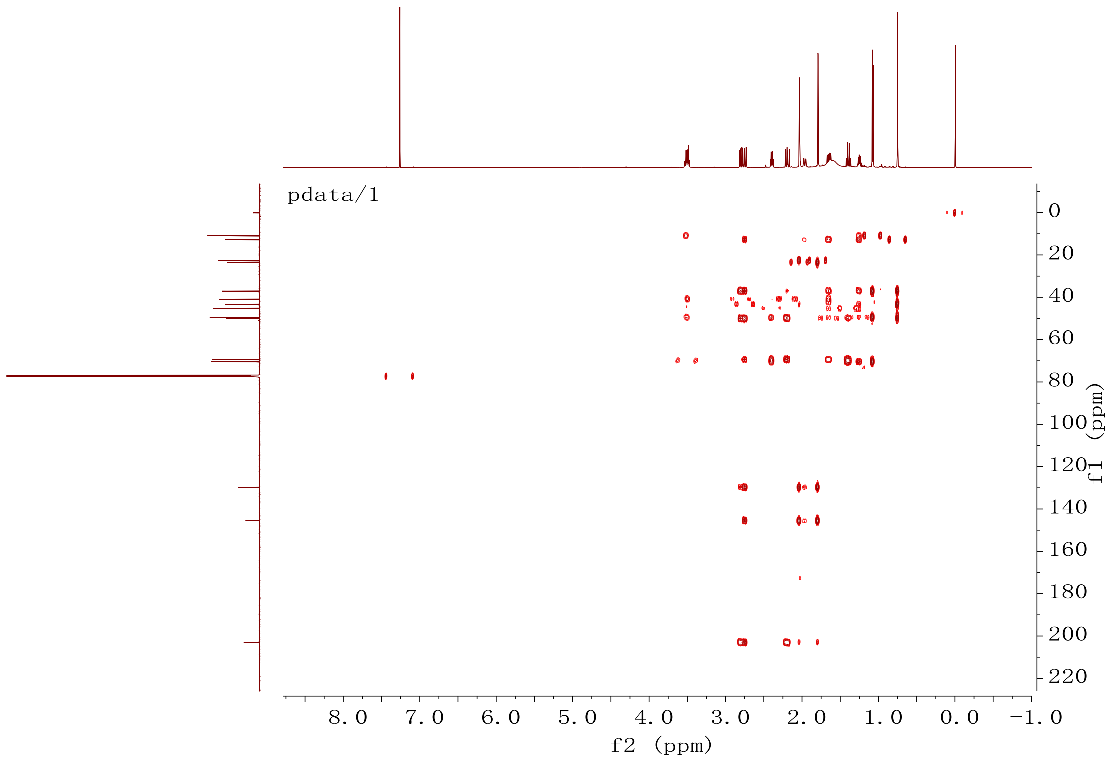

Figure S50. HMBC spectrum of compound **6** ($CDCl_3$).

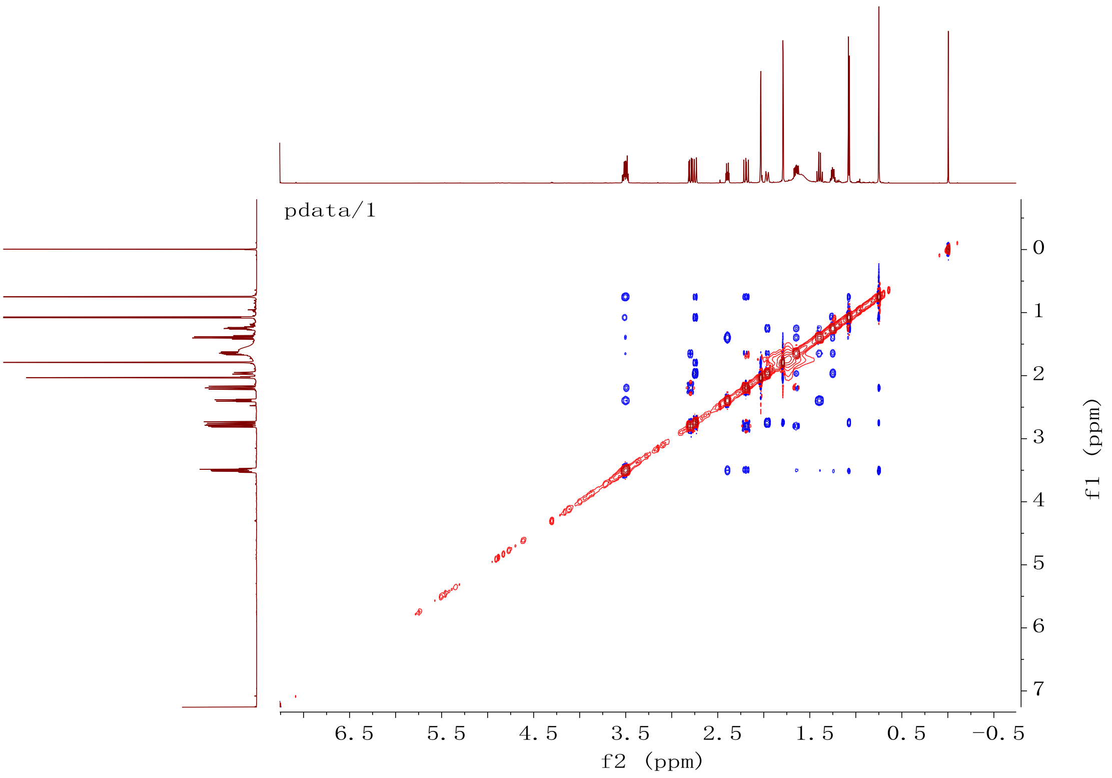

Figure S51. NOESY spectrum of compound **6** ($CDCl_3$).

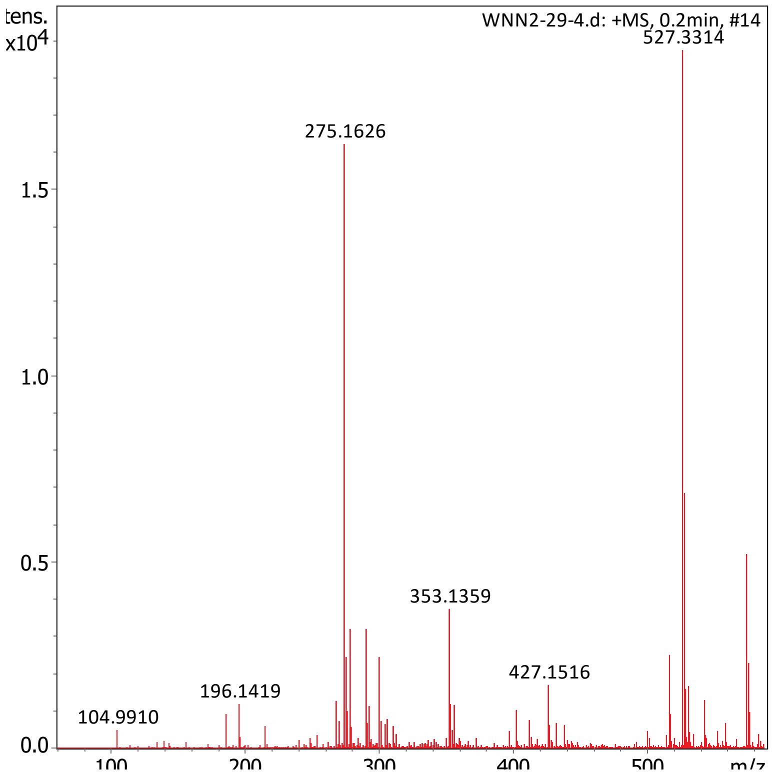

Figure S52. HRESIMS spectrum of compound **6**.

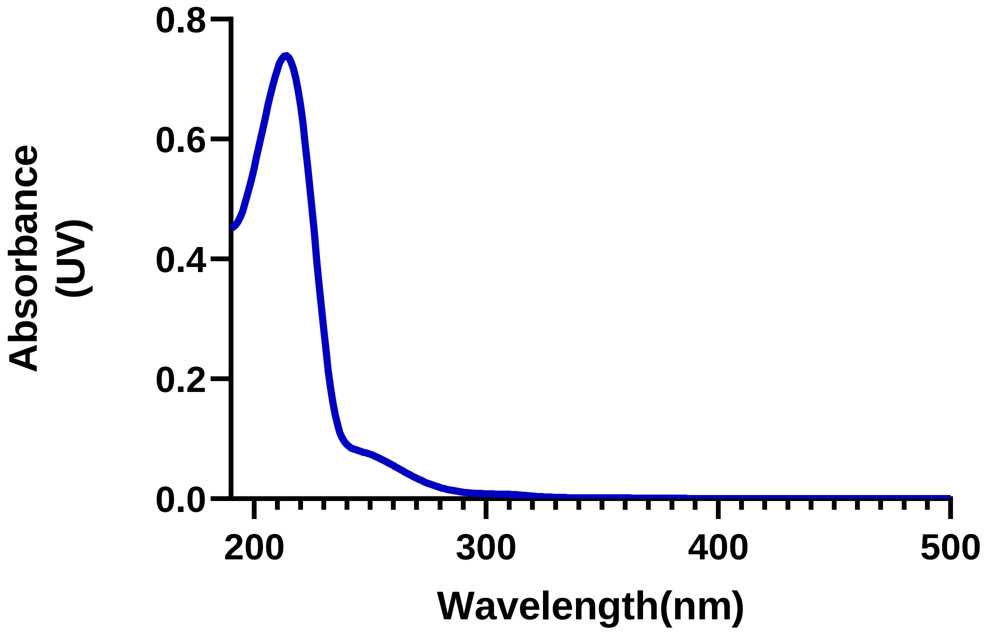

Figure S53. UV spectrum of compound **7** in MeCN.

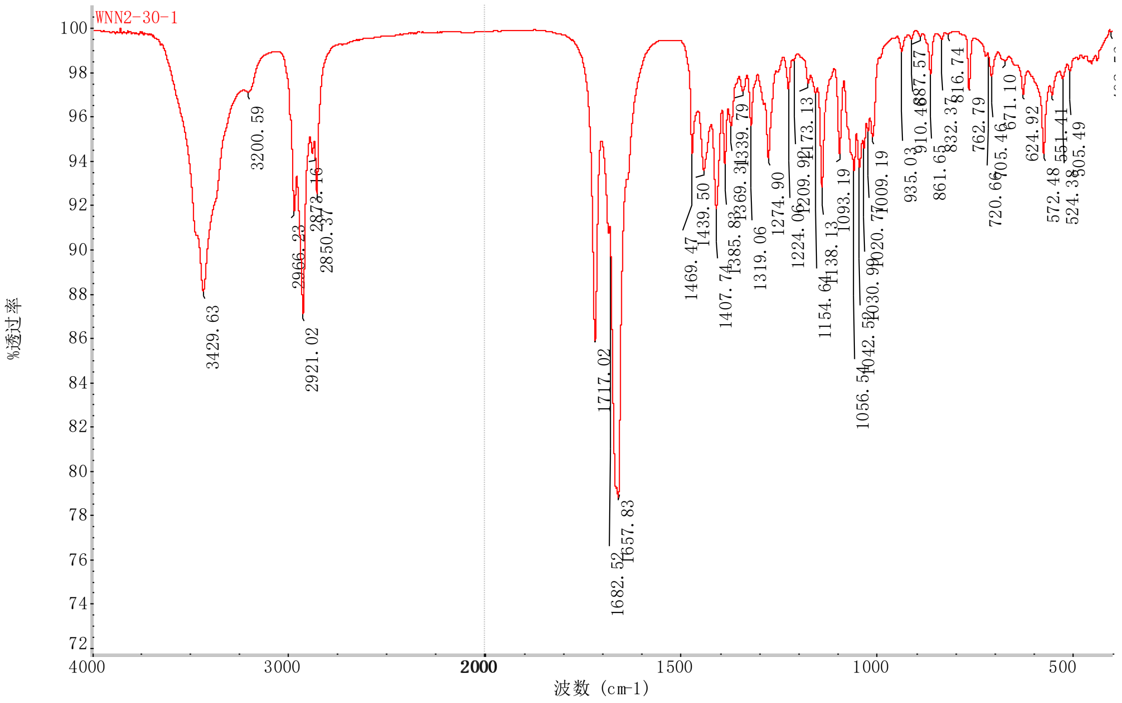

Figure S54. IR spectrum of compound **7**.

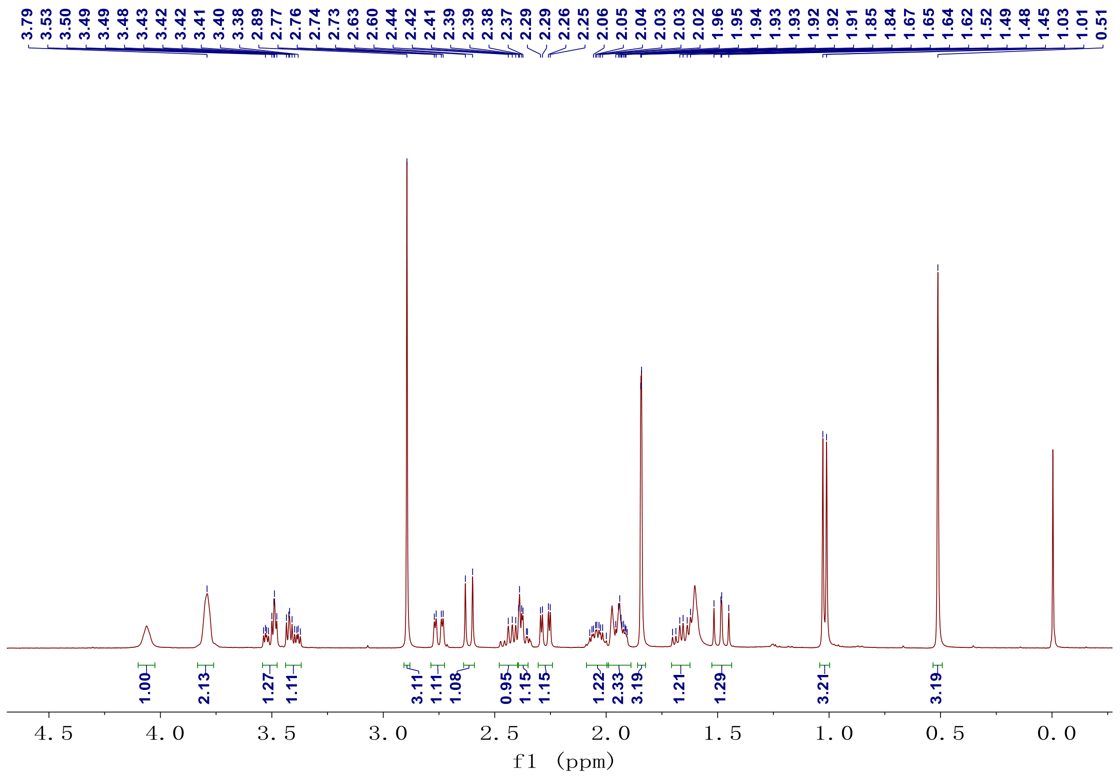

Figure S55. ^{1}H NMR spectrum of compound **7** (400 MHz, $CDCl_3$).

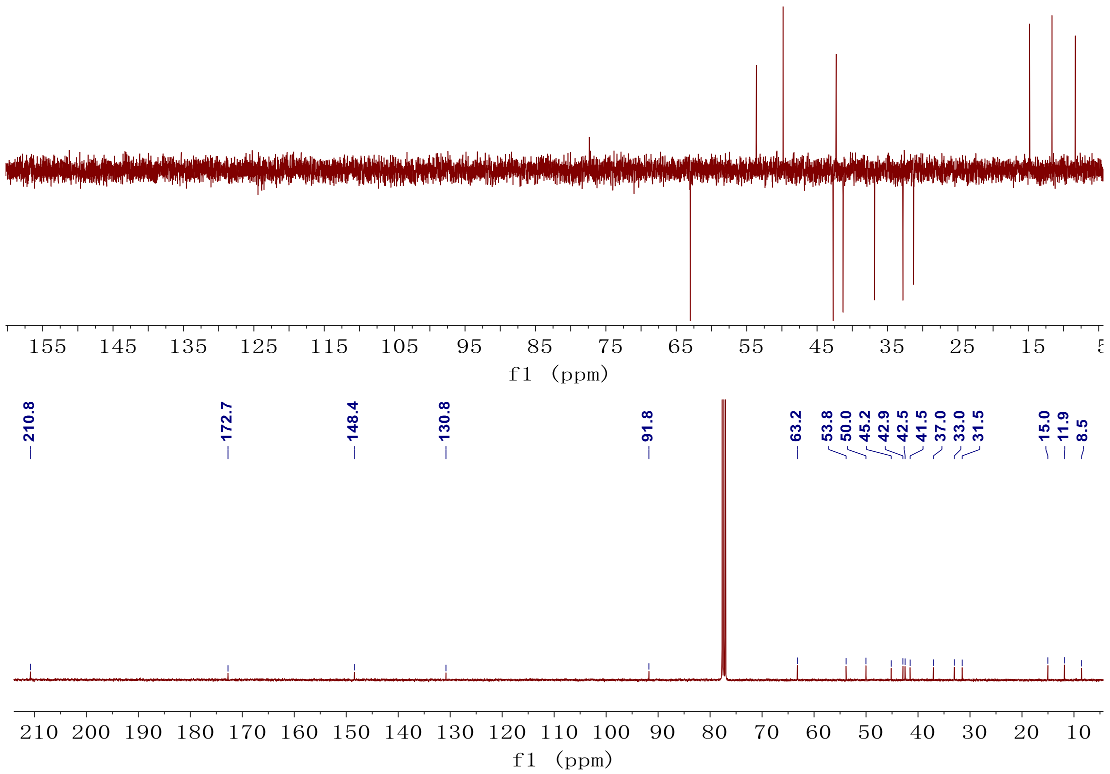

Figure S56. ^{13}C and DEPT-135 NMR spectra of compound **7** (100 MHz, $CDCl_3$).

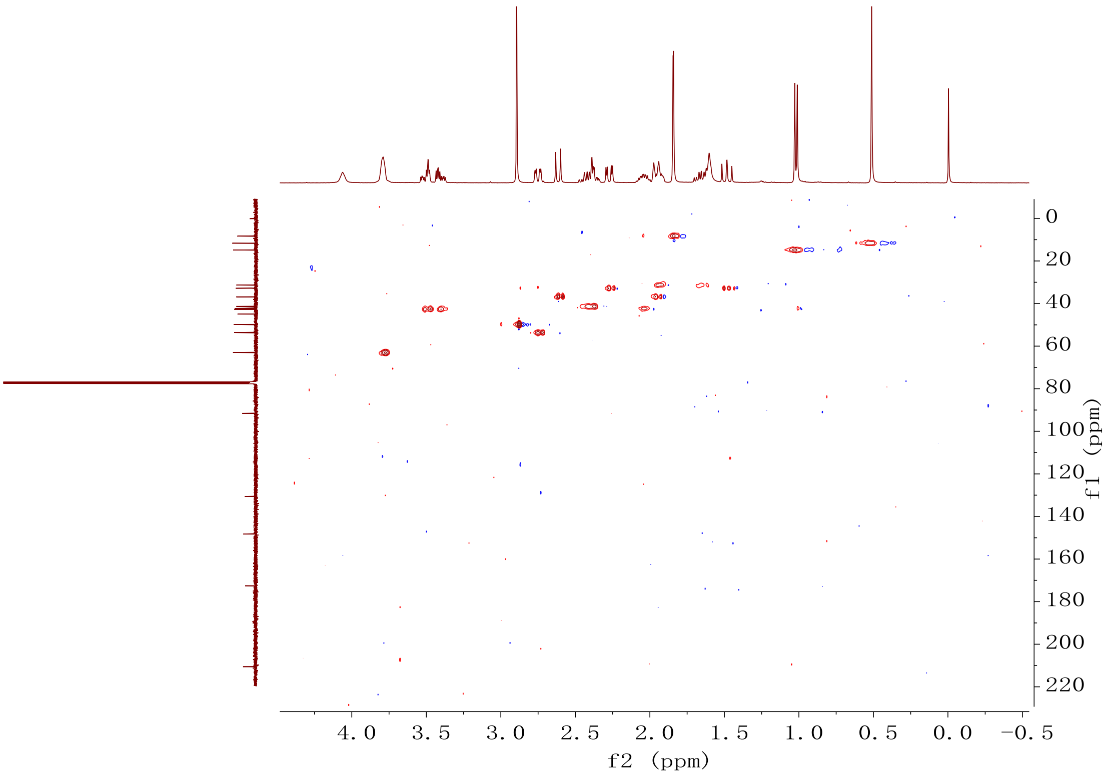

Figure S57. HSQC spectrum of compound **7** ($CDCl_3$).

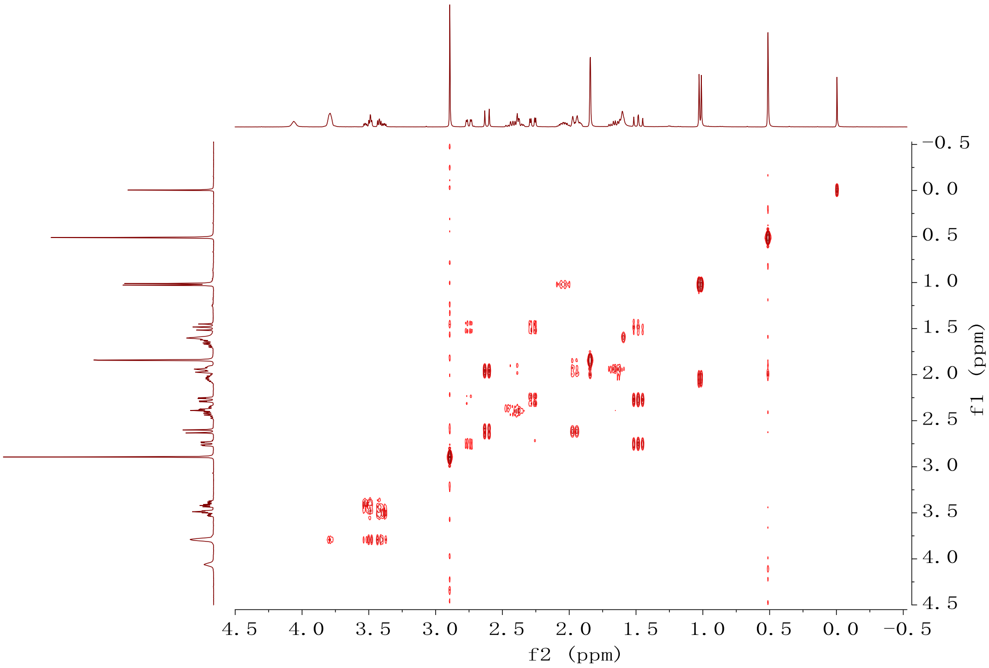

Figure S58. $^{1}H-^{1}H$ COSY spectrum of compound **7** ($CDCl_3$).

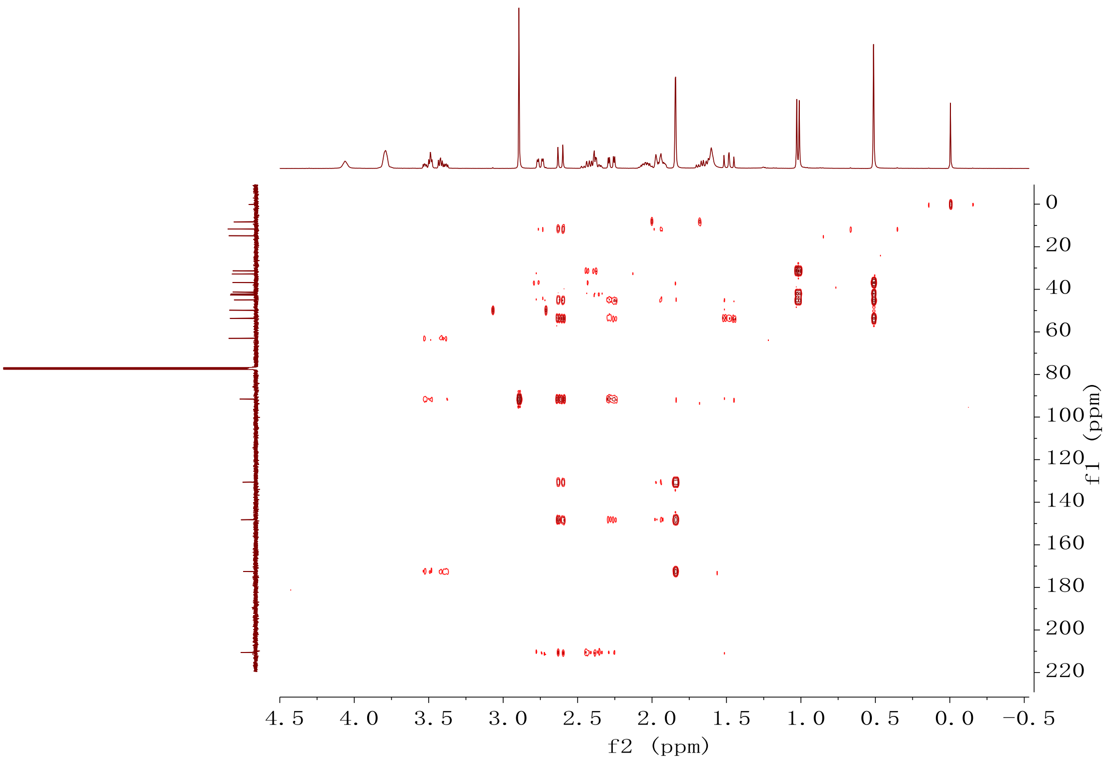

Figure S59. HMBC spectrum of compound **7** ($CDCl_3$).

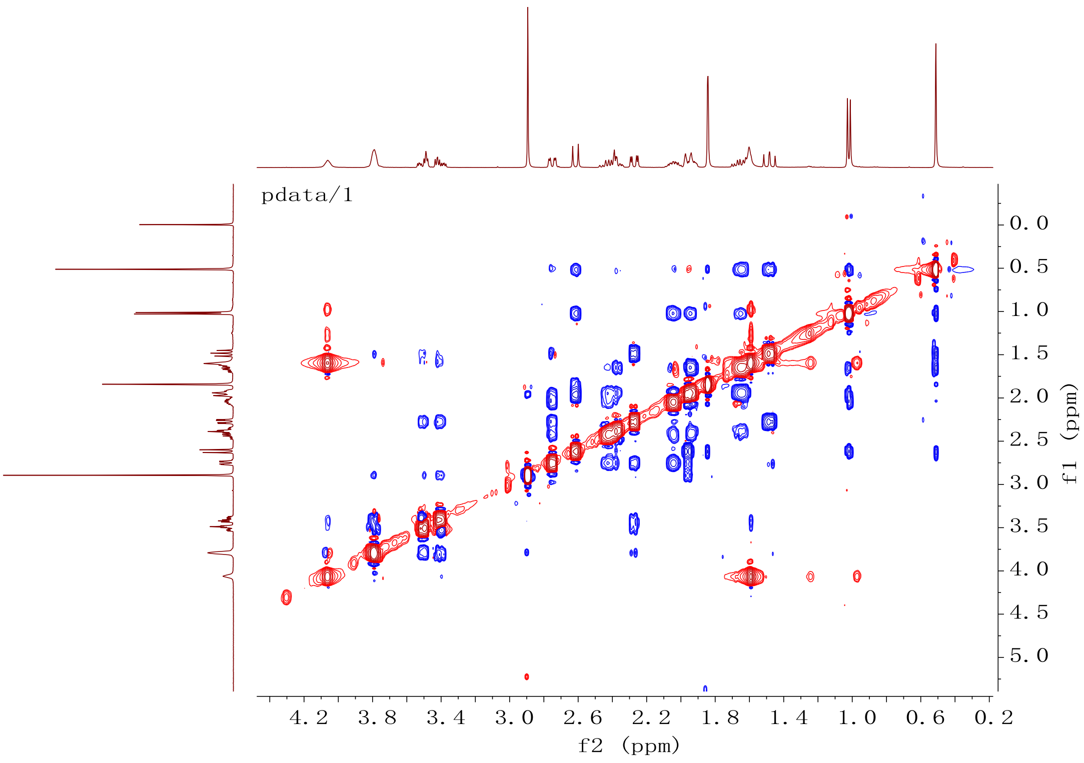

Figure S60. NOESY spectrum of compound **7** ($CDCl_3$).

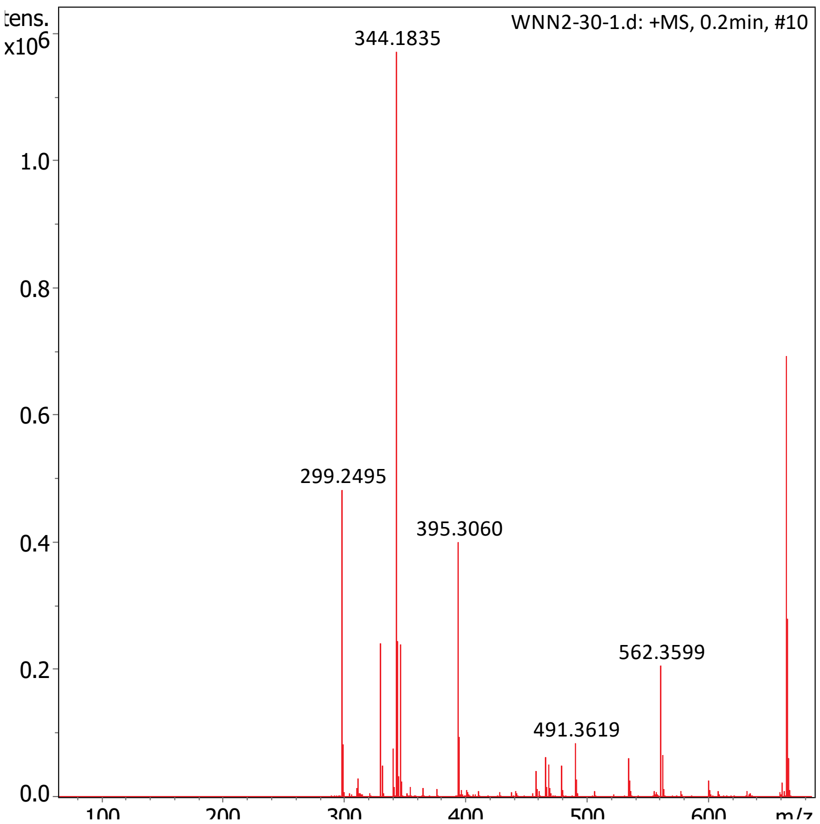

Figure S61. HRESIMS spectrum of compound **7**.

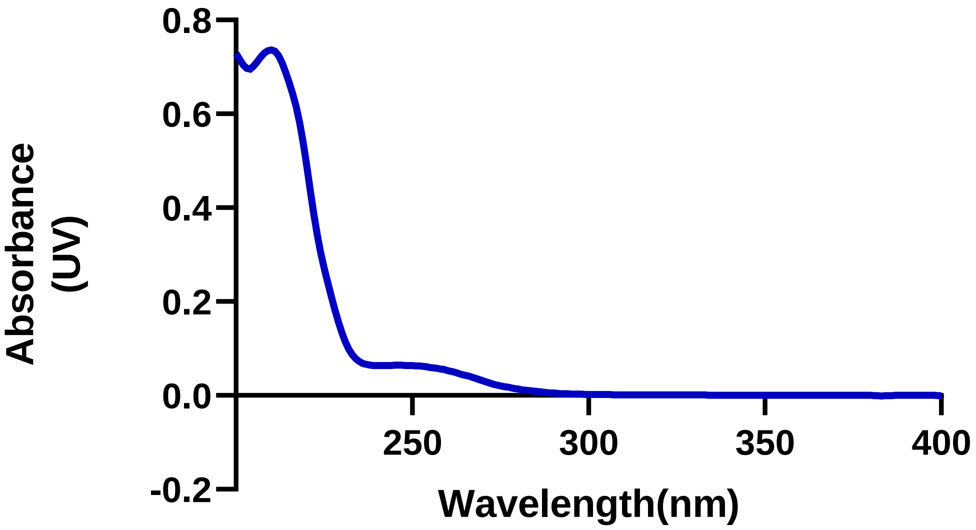

Figure S62. UV spectrum of compound **8** in MeCN.

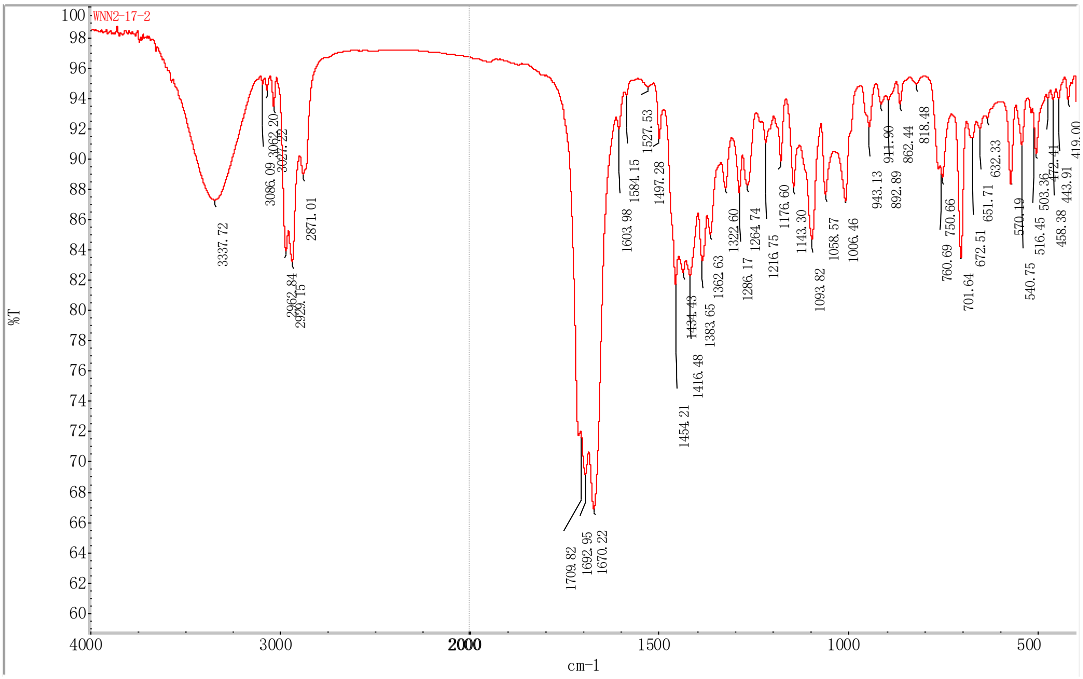

Figure S63. IR spectrum of compound **8**.

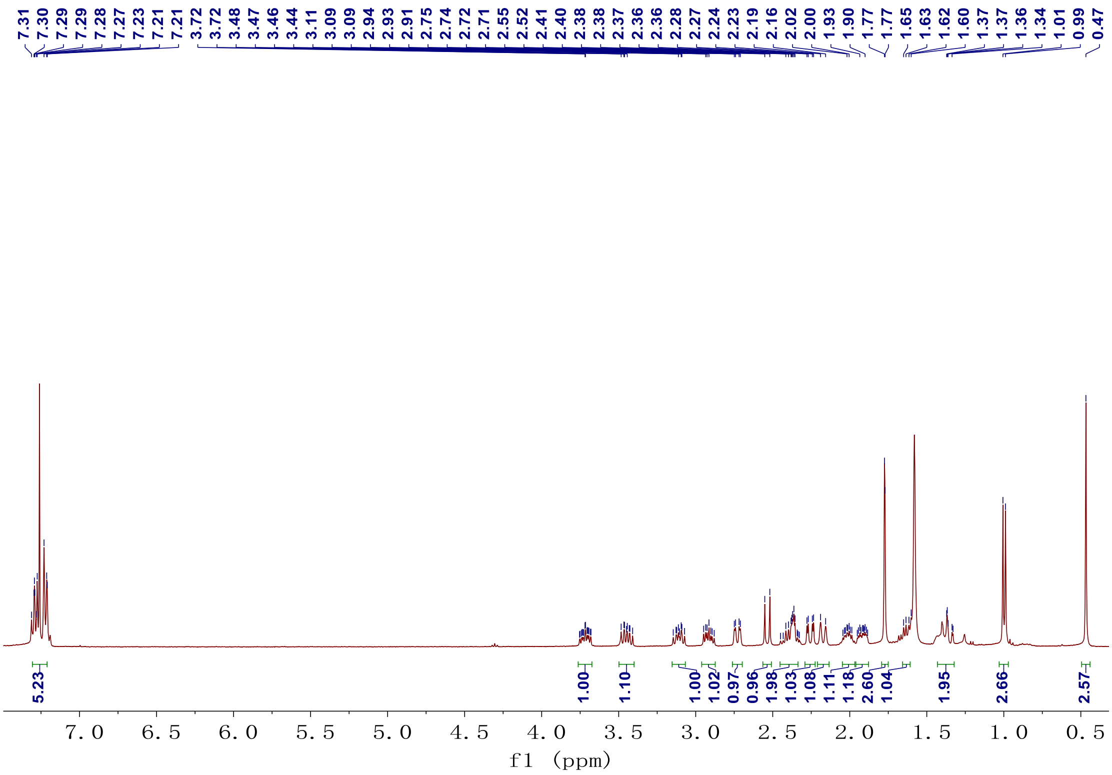

Figure S64. ^{1}H NMR spectrum of compound **8** (400 MHz, $CDCl_3$).

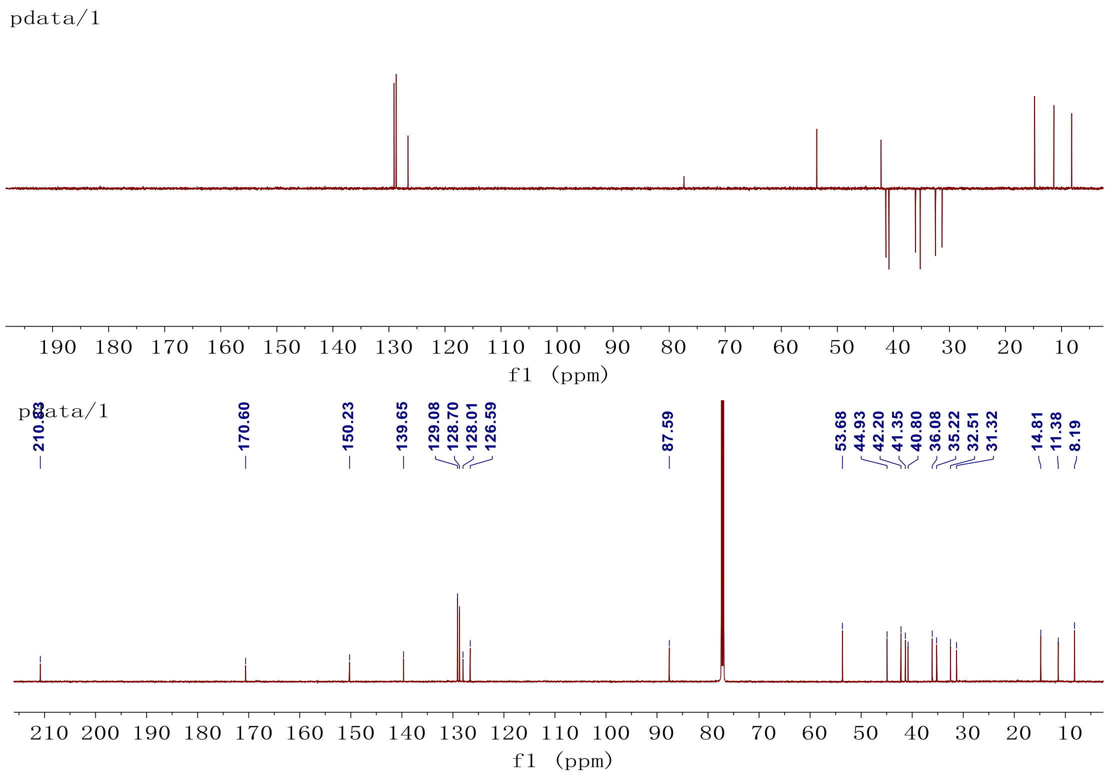

Figure S65. ^{13}C and DEPT-135 NMR spectra of compound **8** (150 MHz, $CDCl_3$).

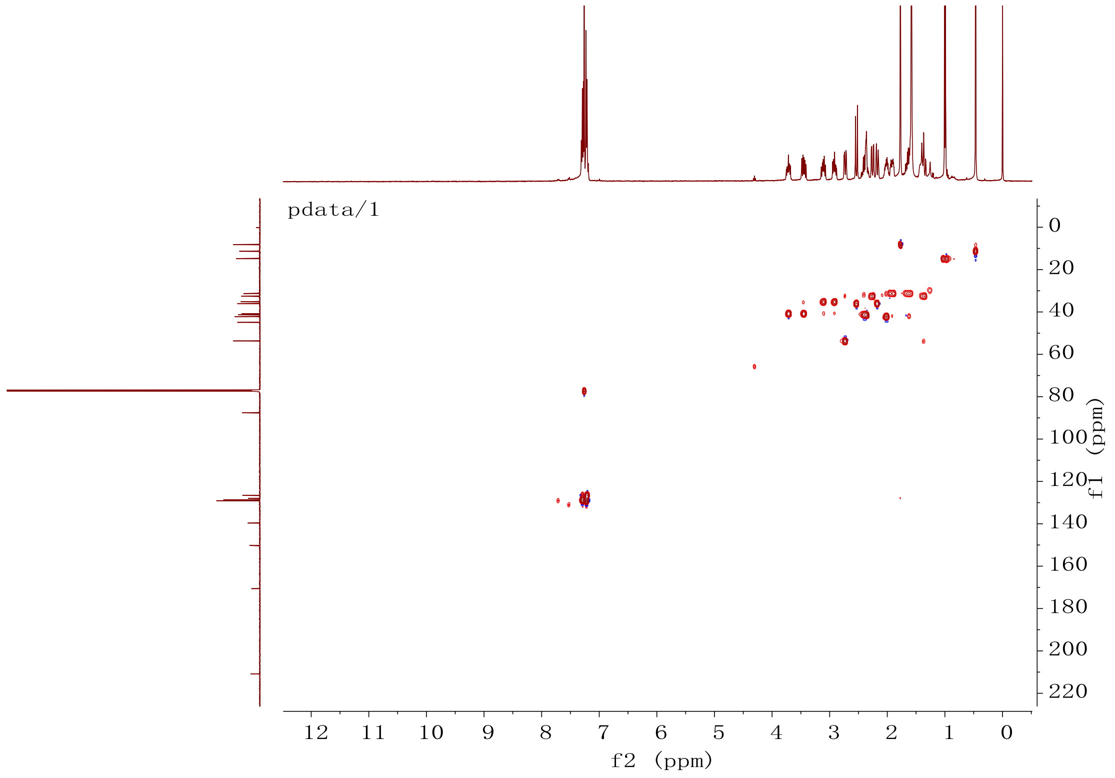

Figure S66. HSQC spectrum of compound **8** ($CDCl_3$).

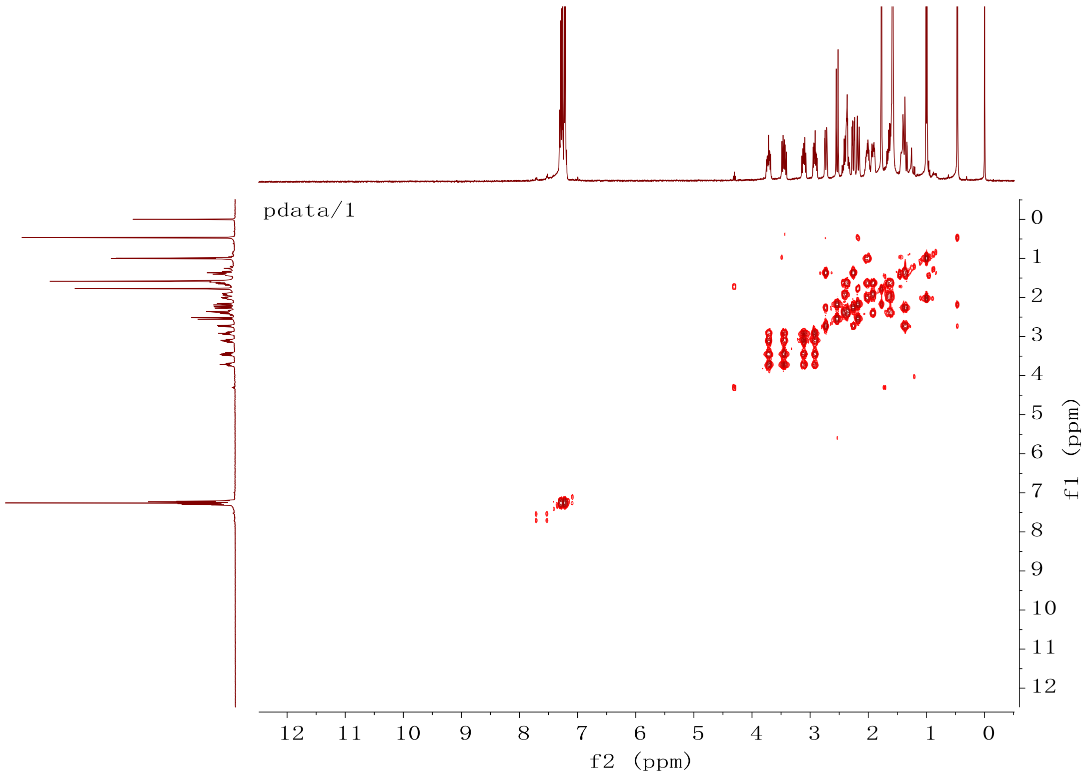

Figure S67. ^{1}H–^{1}H COSY spectrum of compound **8** ($CDCl_3$).

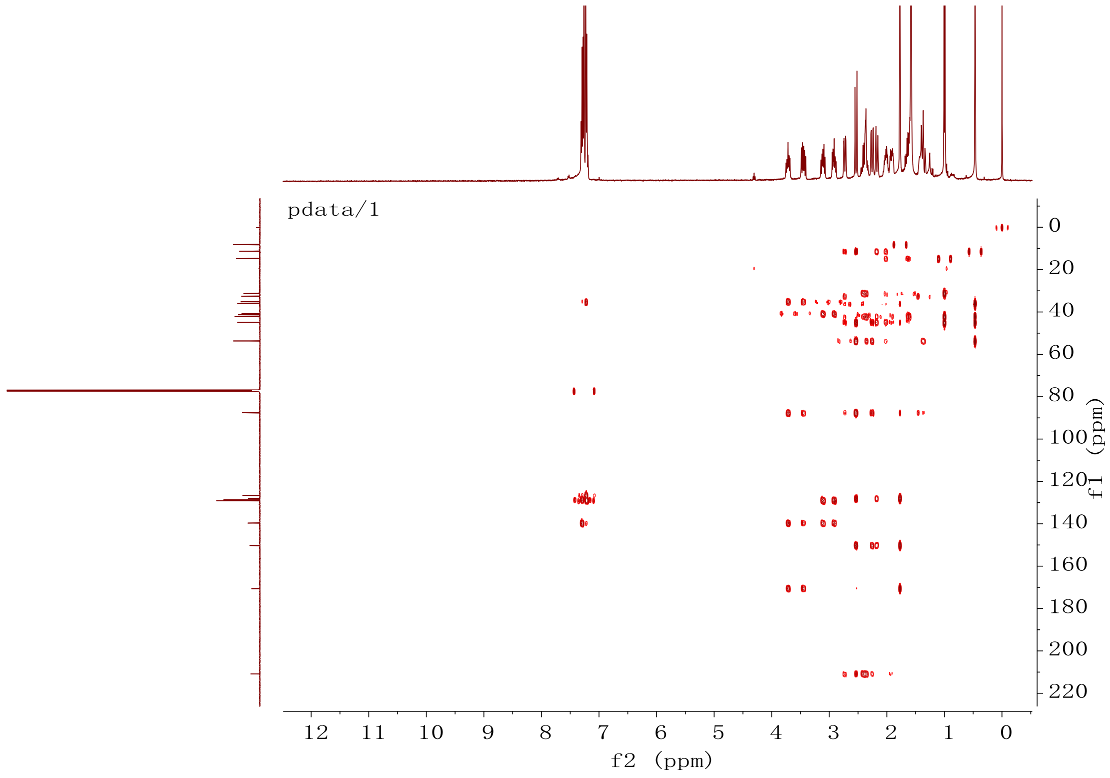

Figure S68. HMBC spectrum of compound **8** ($CDCl_3$).

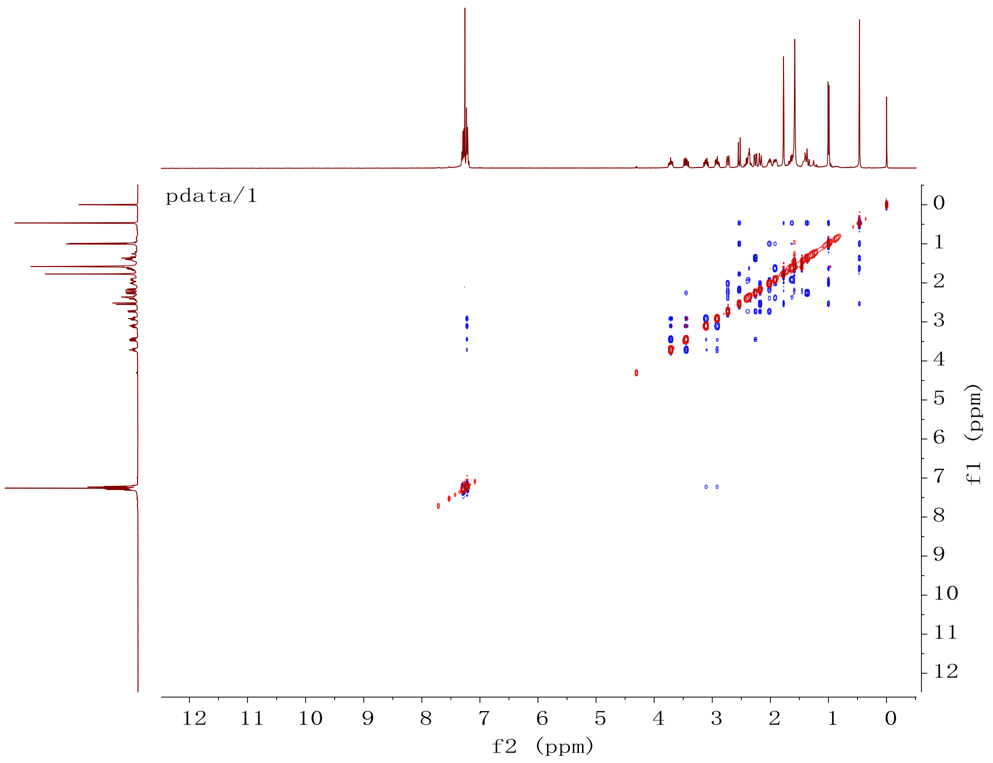

Figure S69. NOESY spectrum of compound **8** ($CDCl_3$).

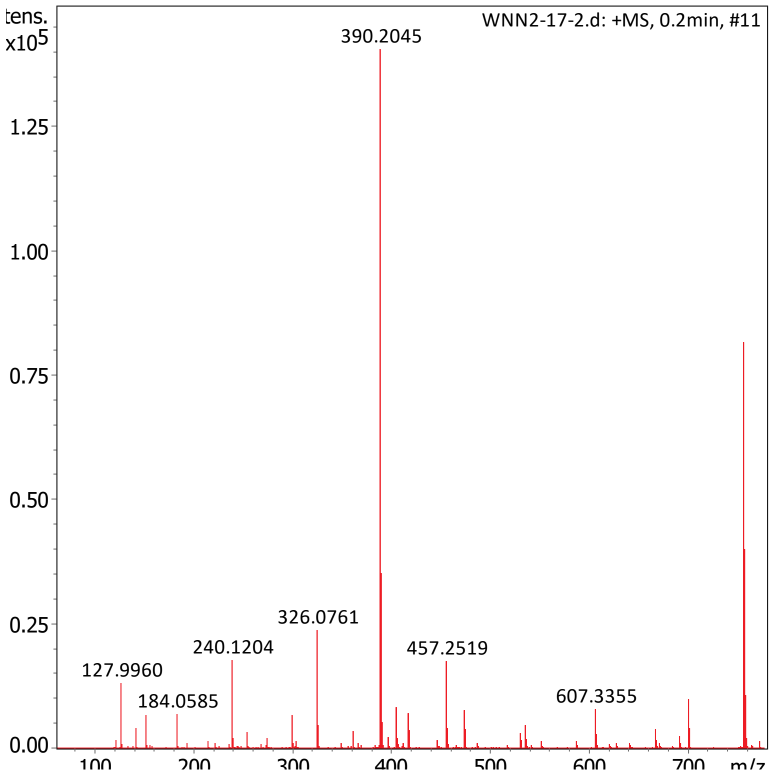

Figure S70. HRESIMS spectrum of compound **8**.

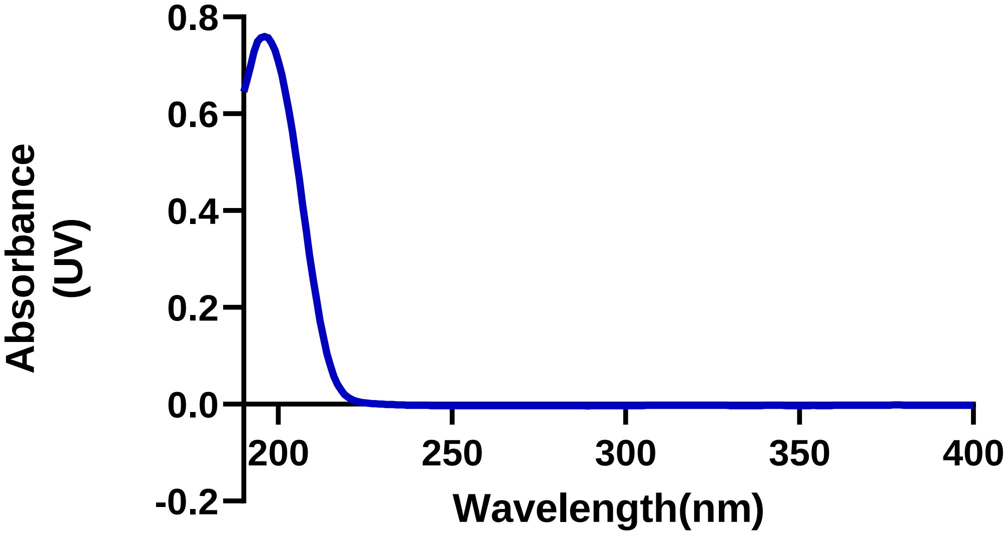

Figure S71. UV spectrum of compound **9** in MeCN.

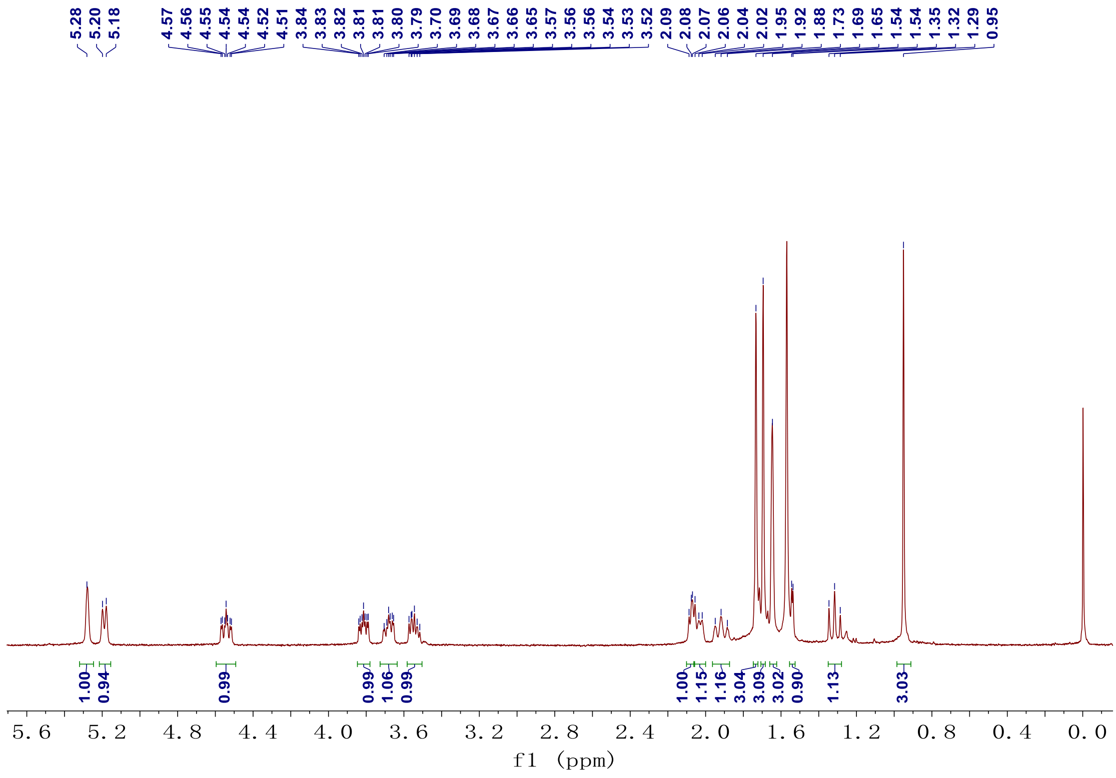

Figure S72. ^{1}H NMR spectrum of compound **9** (600 MHz, $CDCl_3$).

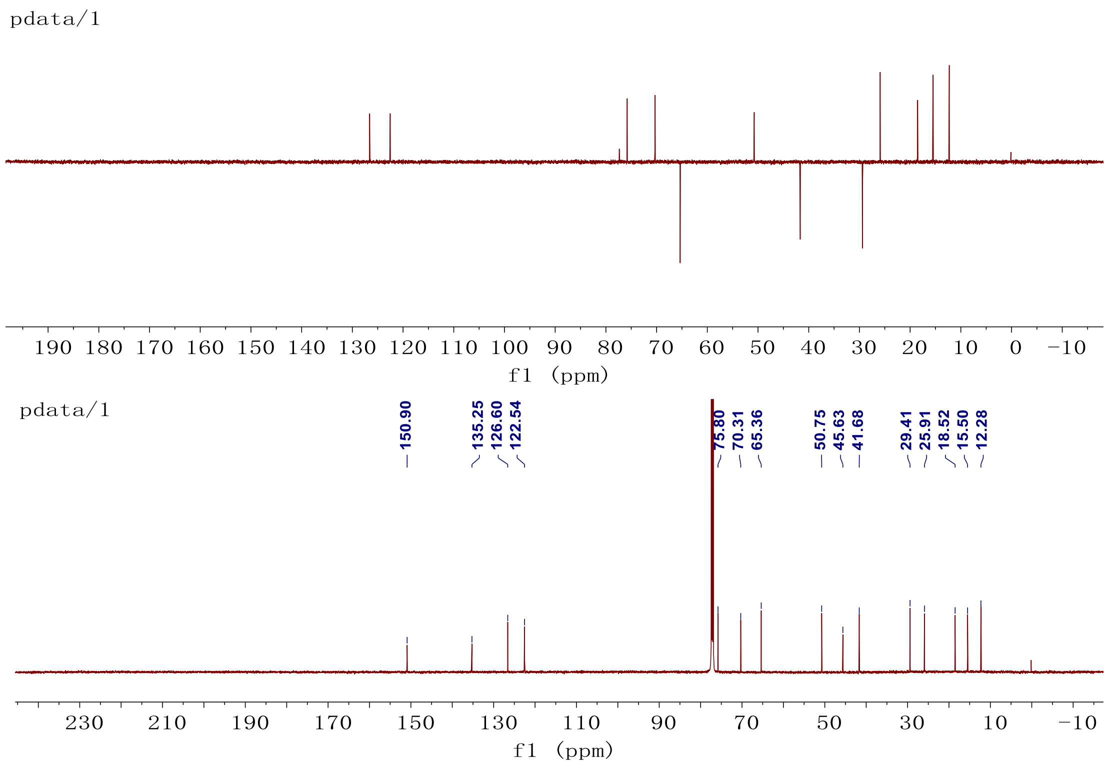

Figure S73. ^{13}C and DEPT-135 NMR spectra of compound **9** (150 MHz, $CDCl_3$).

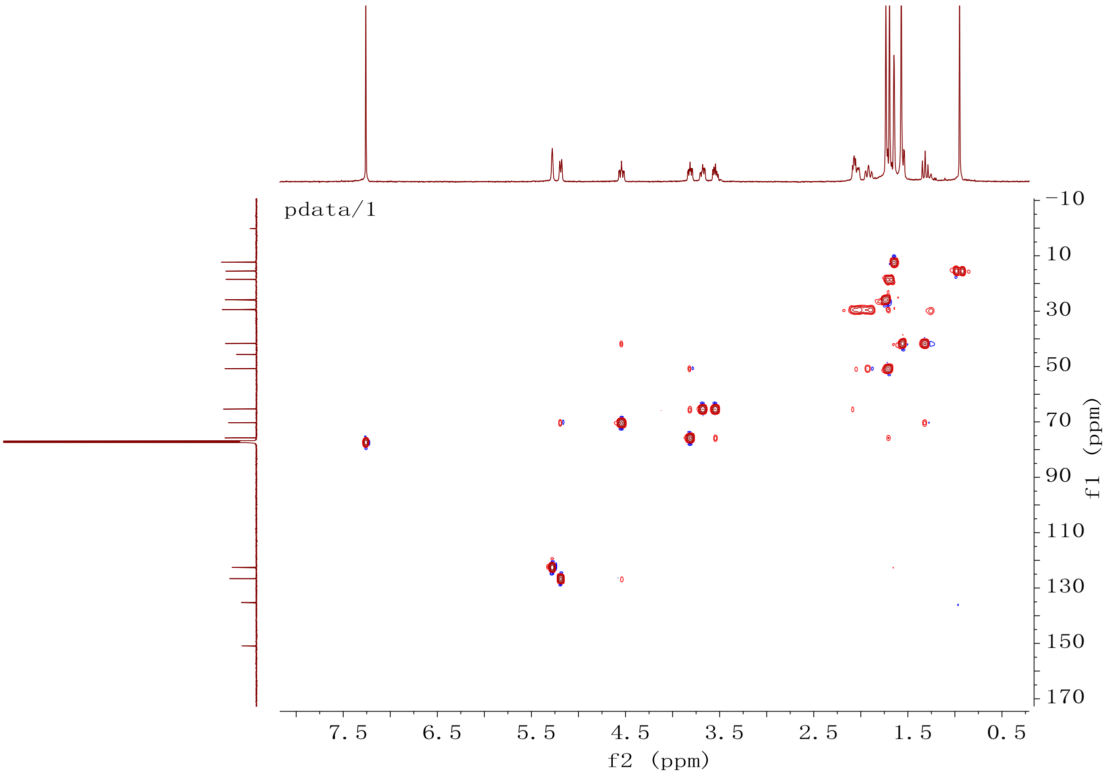

Figure S74. HSQC spectrum of compound **9** ($CDCl_3$).

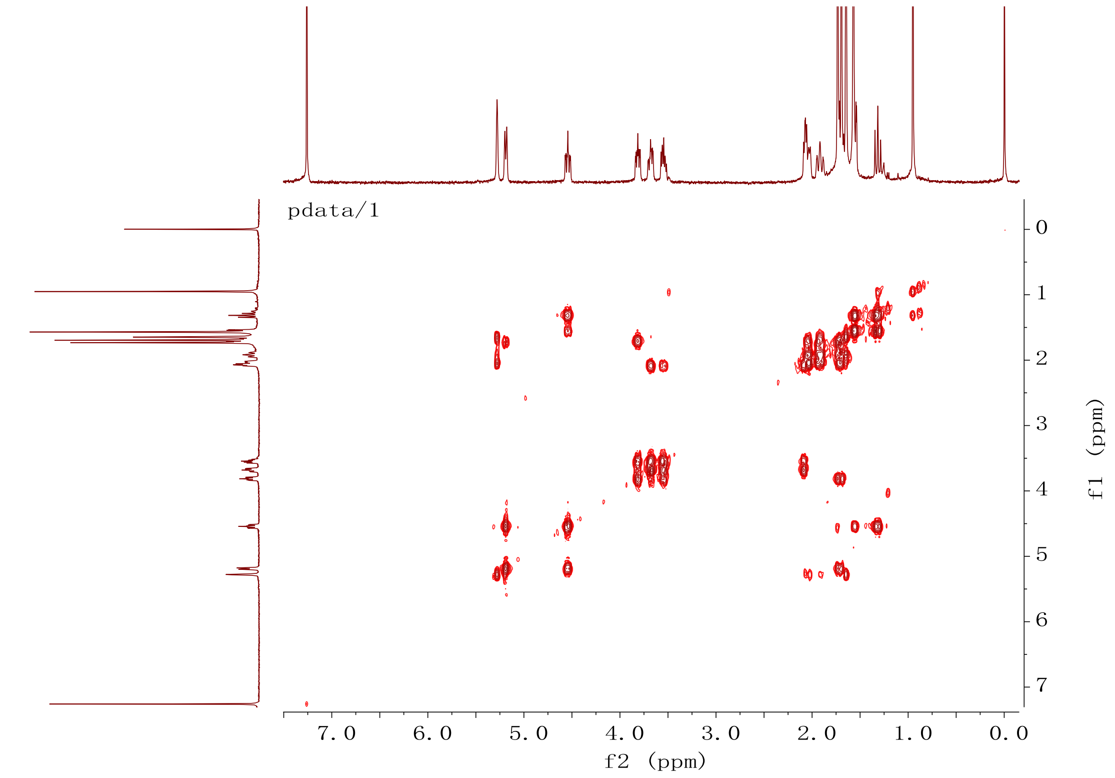

Figure S75. ^1H–^1H COSY spectrum of compound **9** ($CDCl_3$).

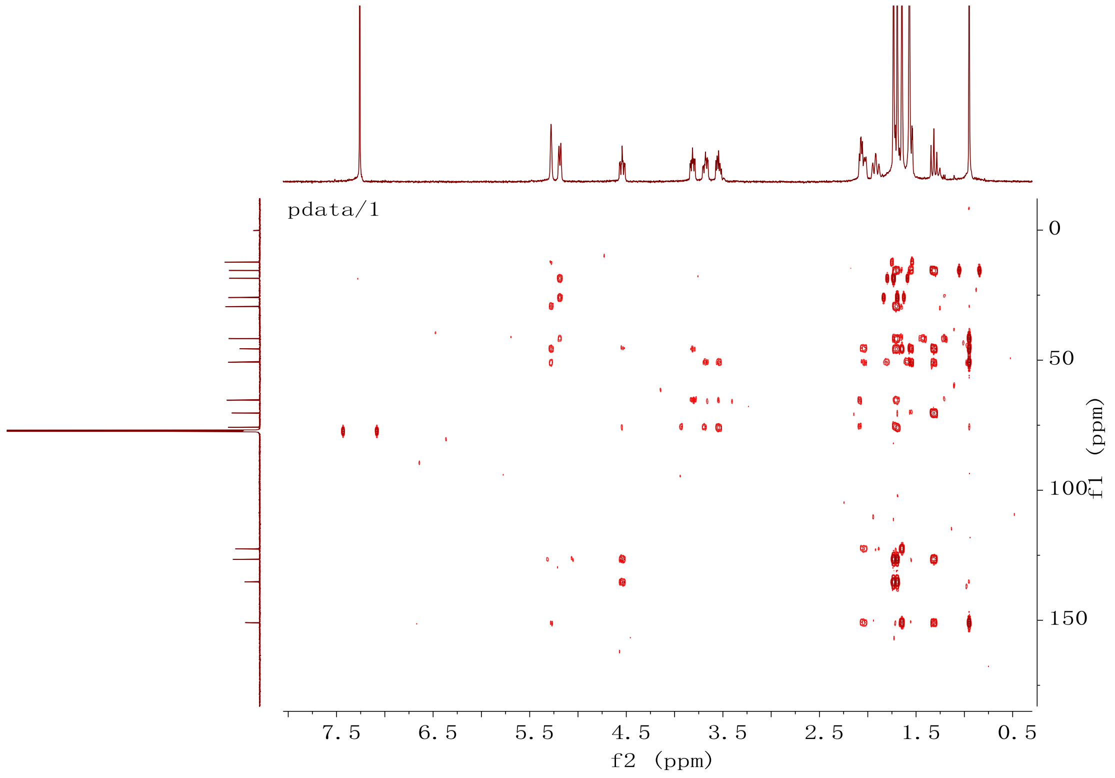

Figure S76. HMBC spectrum of compound **9** ($CDCl_3$).

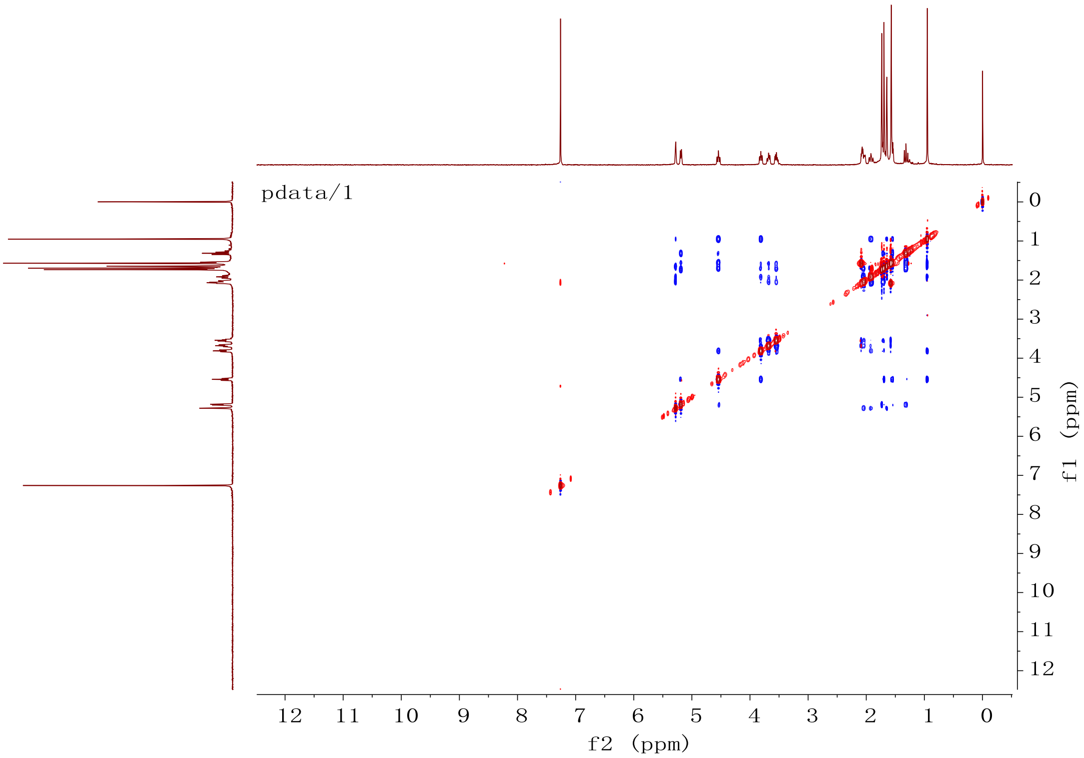

Figure S77. NOESY spectrum of compound **9** ($CDCl_3$).

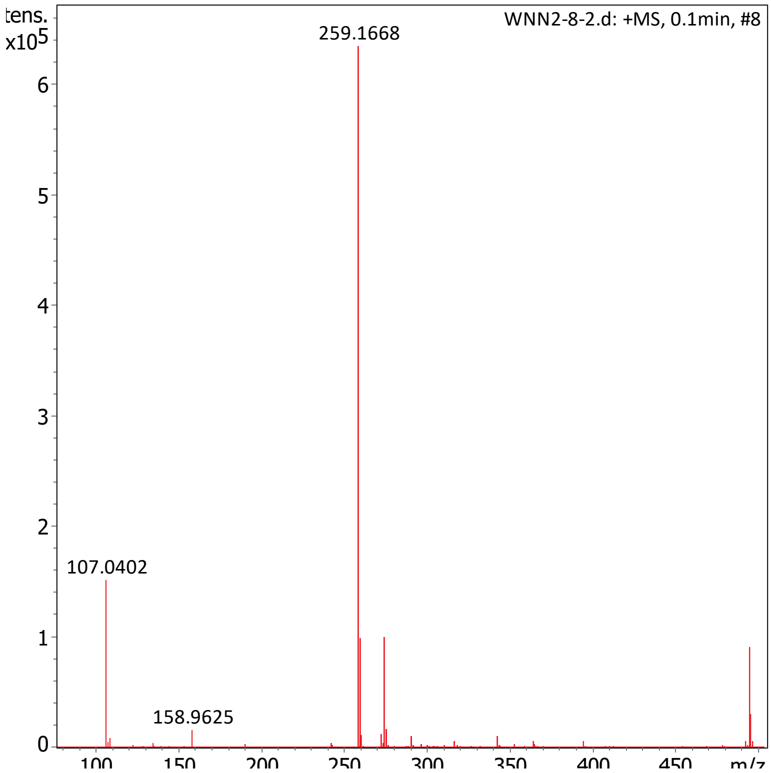

Figure S78. HRESIMS spectrum of compound **9**.

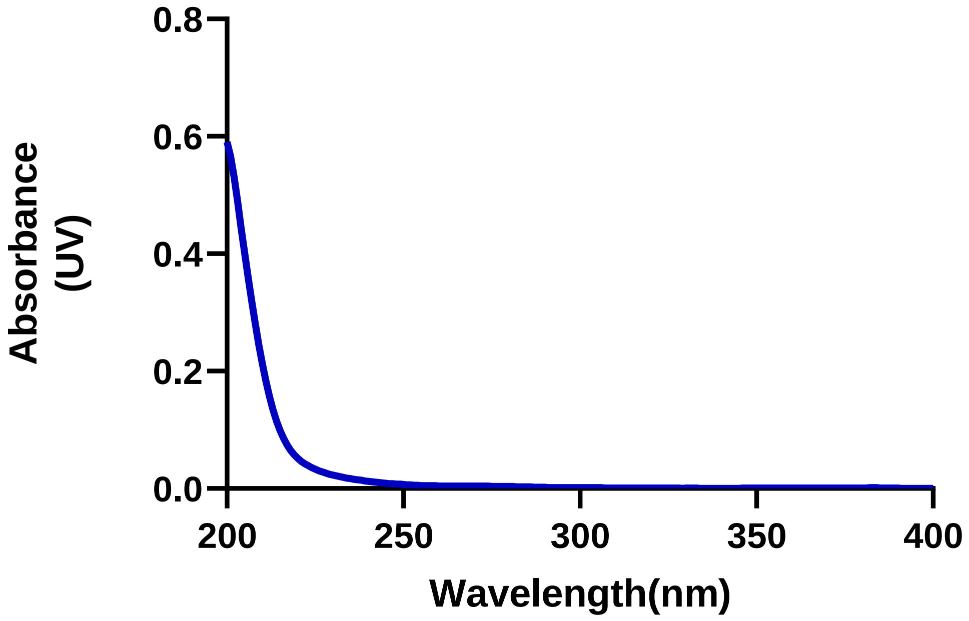

Figure S79. UV spectrum of compound **10** in MeCN.

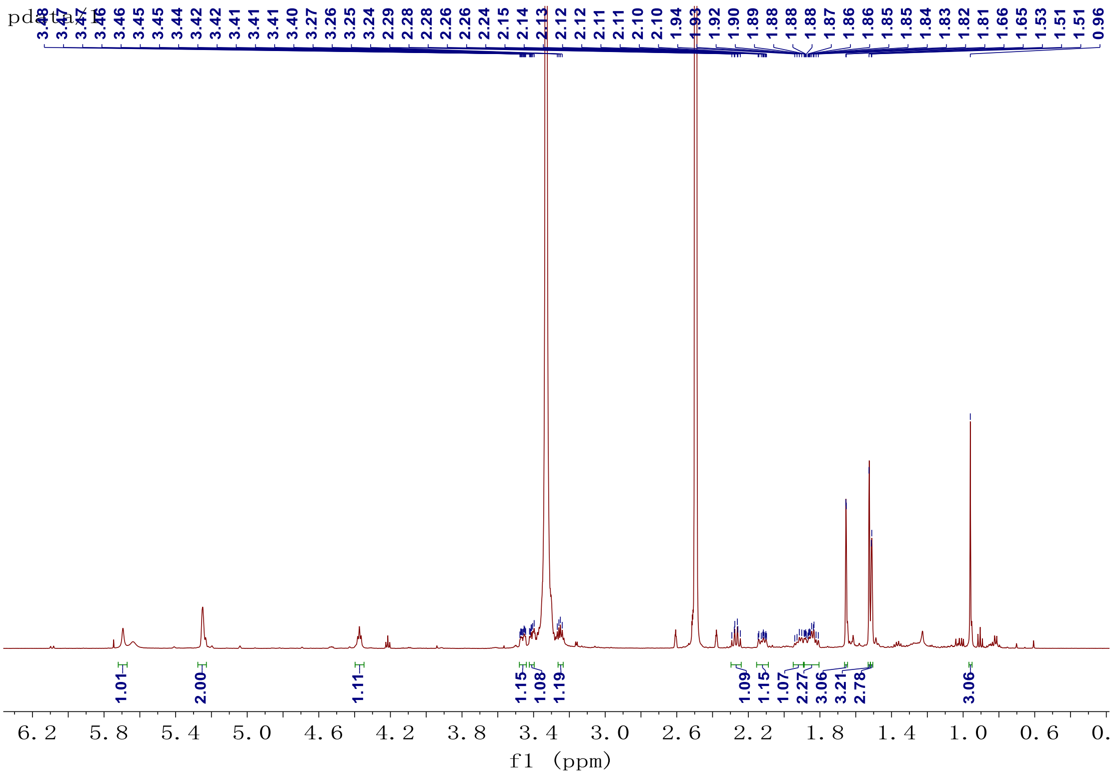

Figure S80. ^{1}H NMR spectrum of compound **10** (600 MHz, $CDCl_3$).

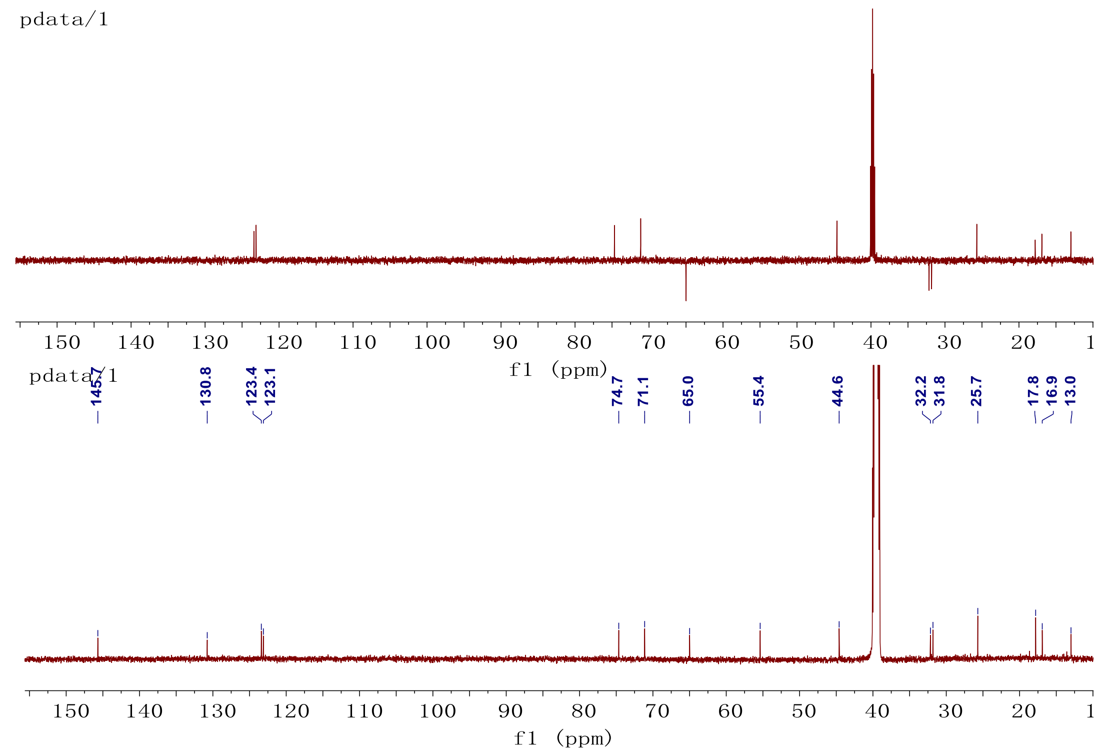

Figure S81. ^{13}C and DEPT-135 NMR spectra of compound **10** (150 MHz, $CDCl_3$).

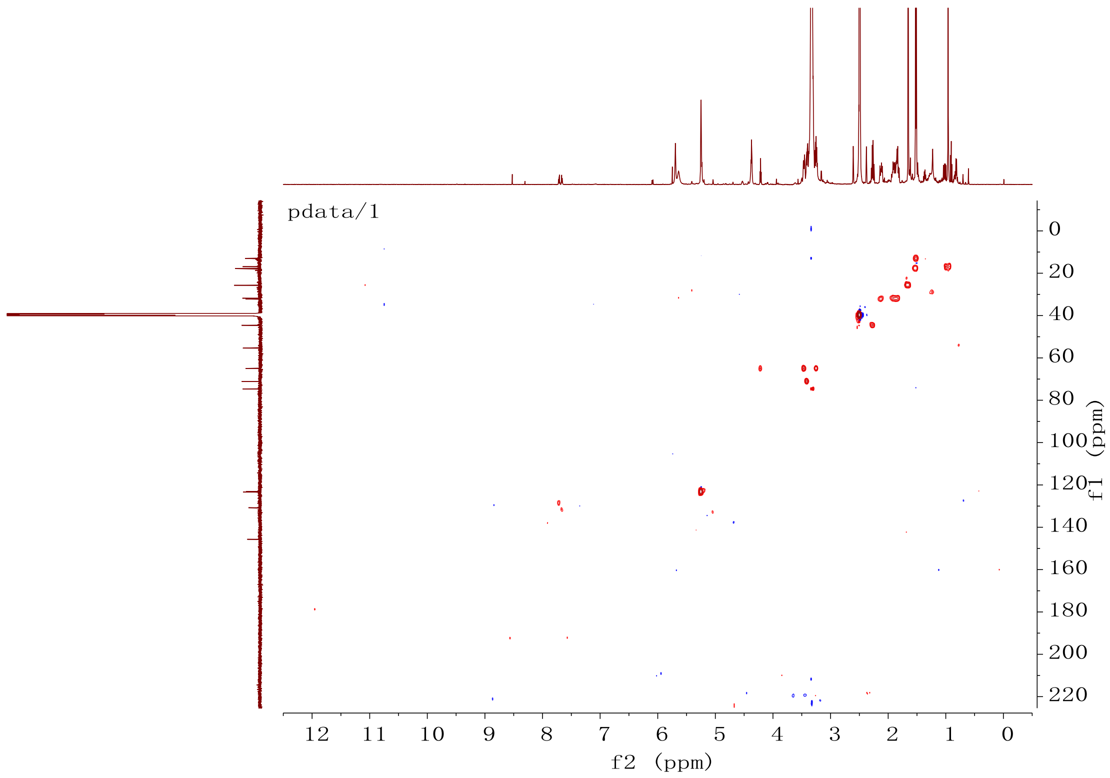

Figure S82. HSQC spectrum of compound **10** ($CDCl_3$).

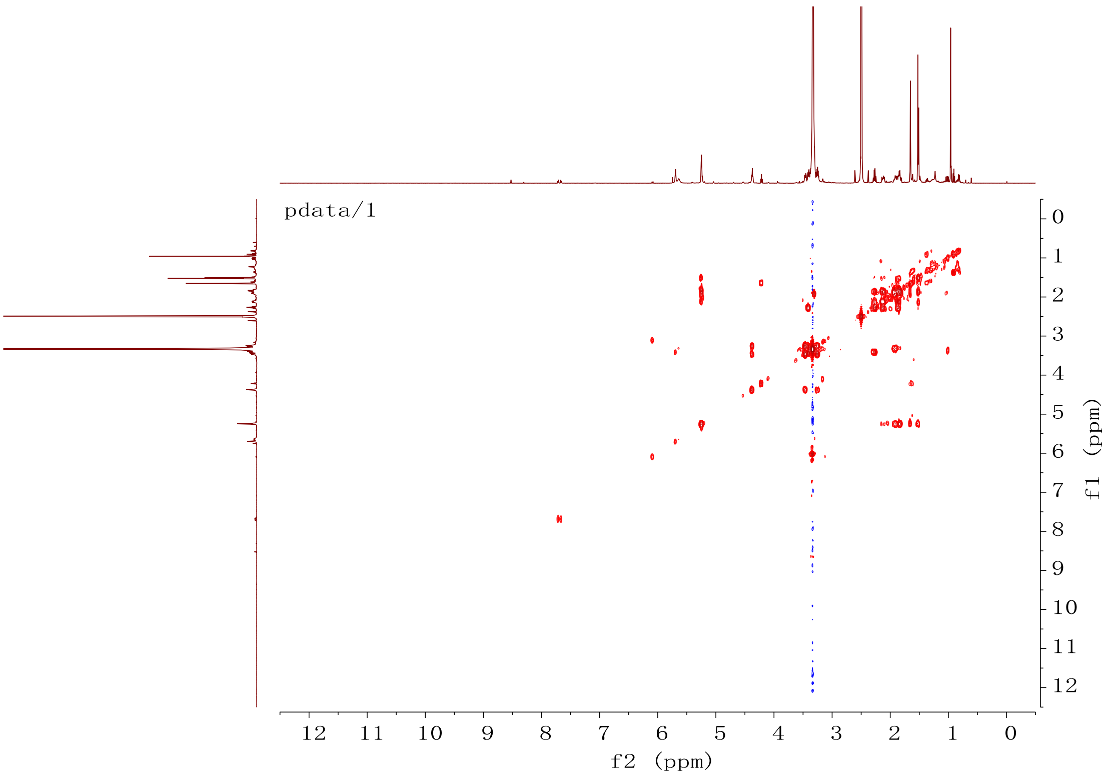

Figure S83. ^{1}H–^{1}H COSY spectrum of compound **10** ($CDCl_3$).

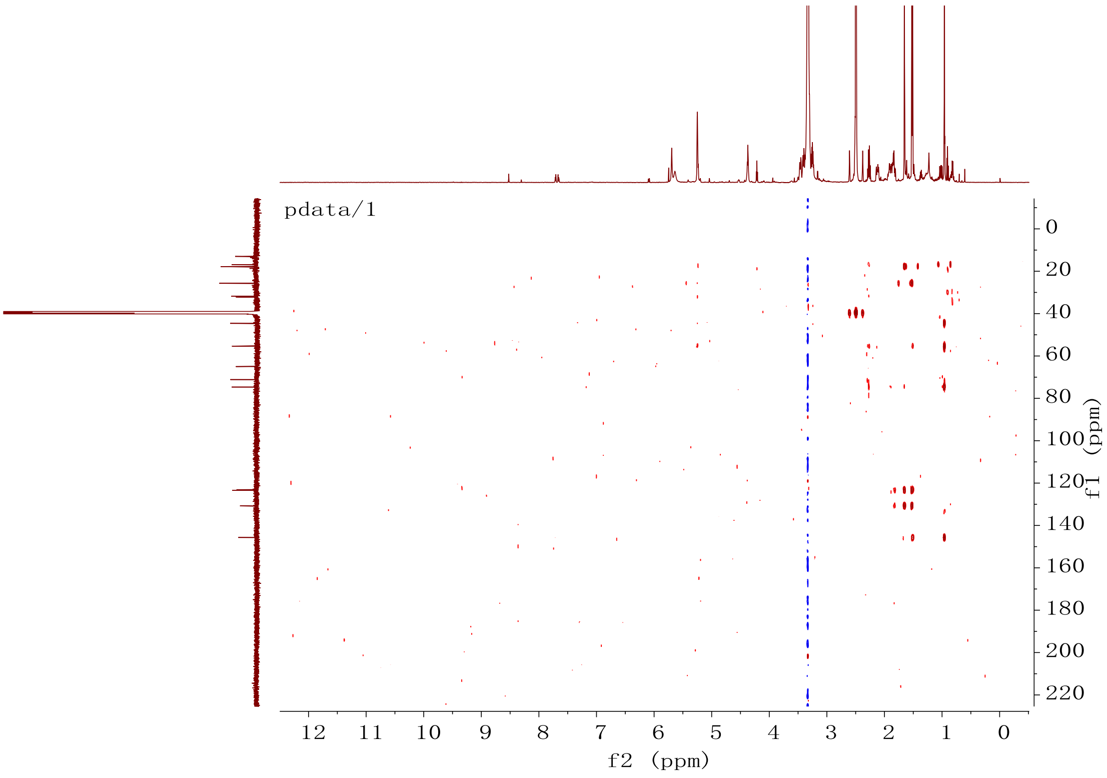

Figure S84. HMBC spectrum of compound **10** ($CDCl_3$).

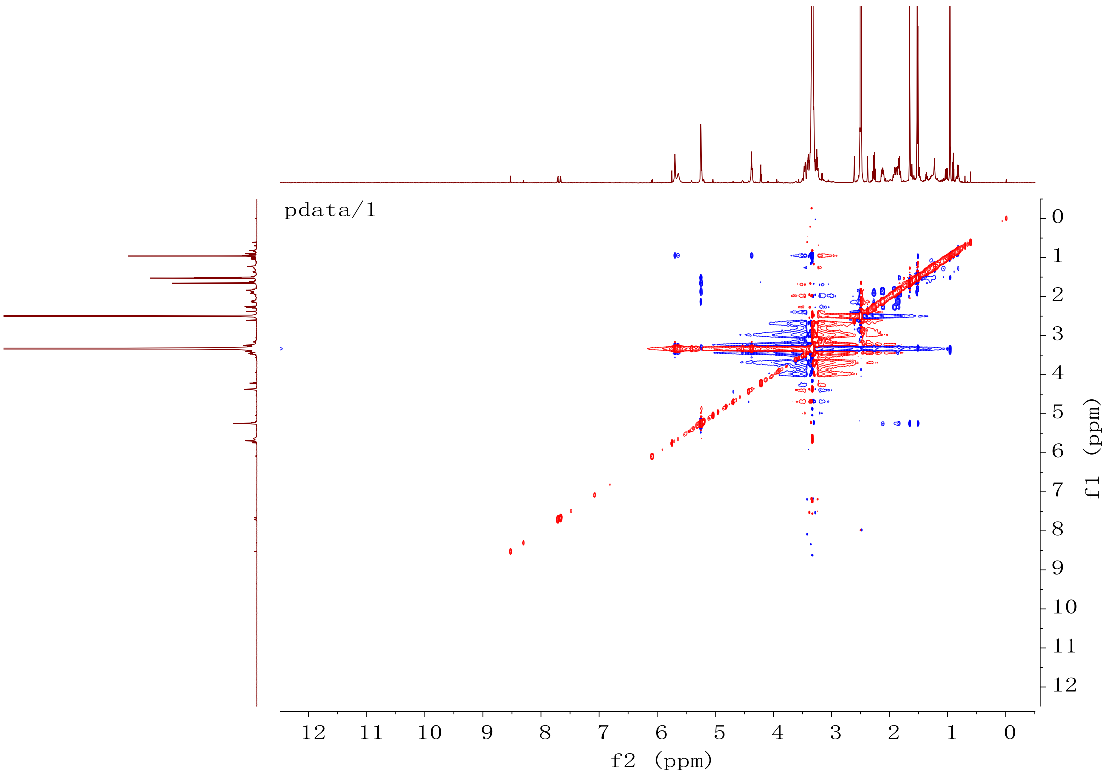

Figure S85. NOESY spectrum of compound **10** ($CDCl_3$).

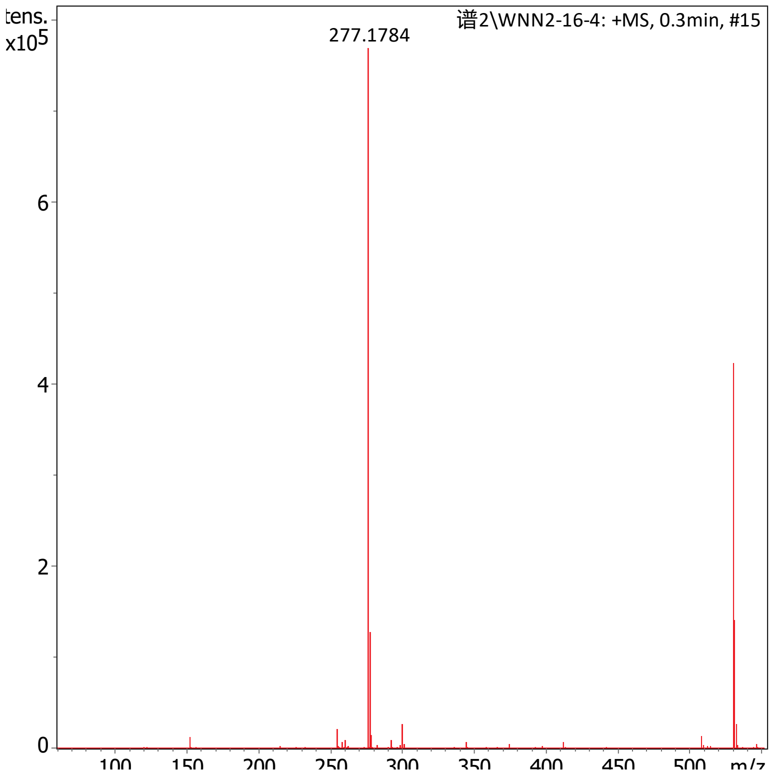

Figure S86. HRESIMS spectrum of compound **10**.

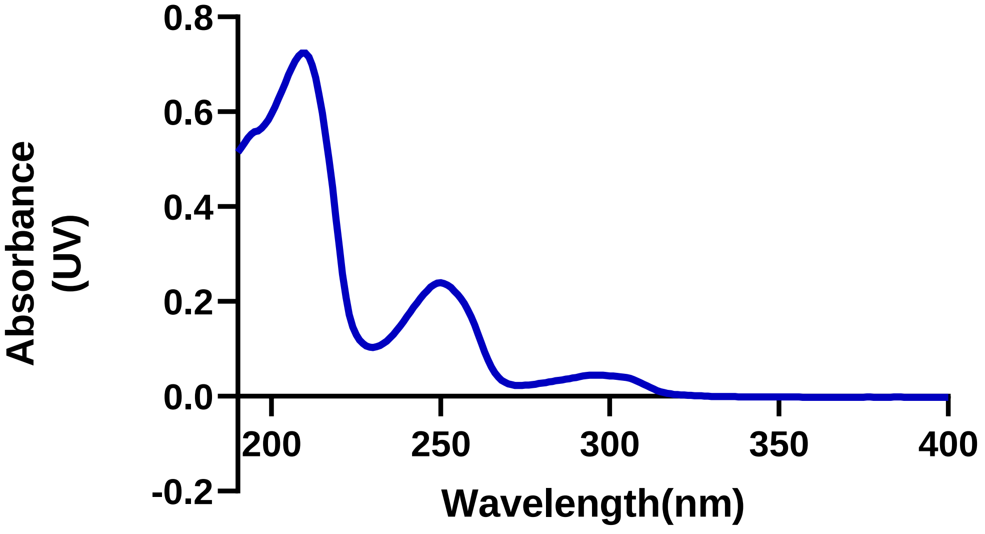

Figure S87. UV spectrum of compound **11** in MeCN.

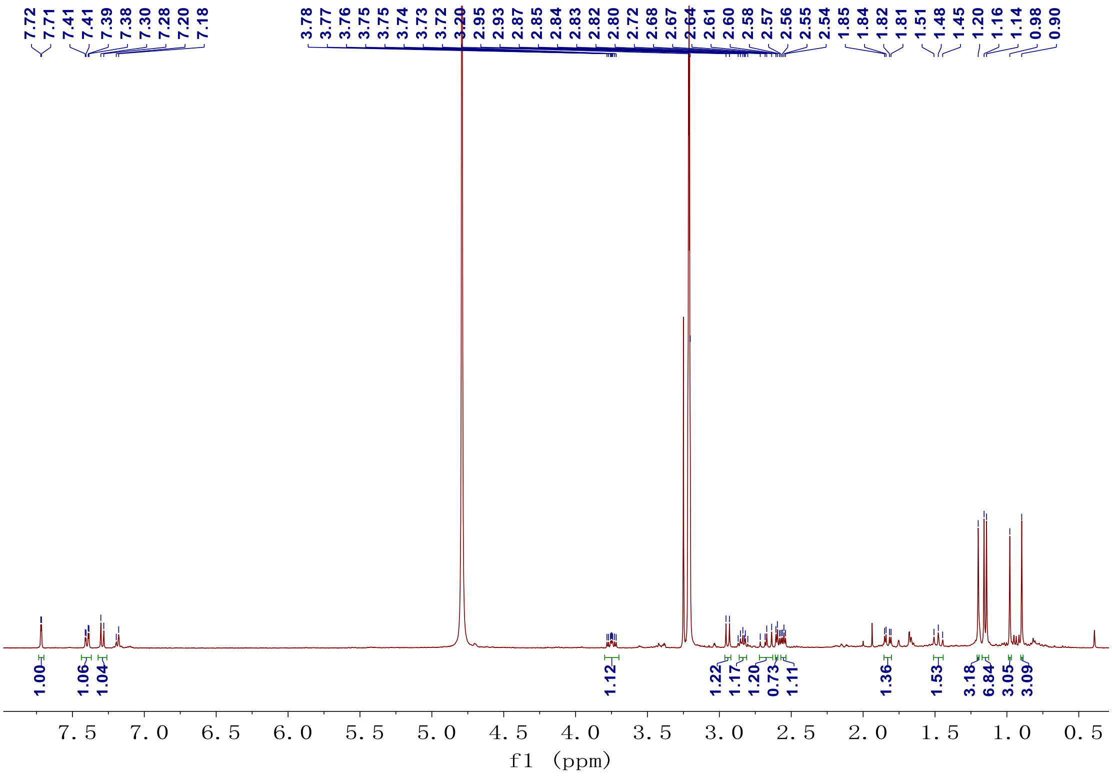

Figure S88. ^{1}H NMR spectrum of compound **11** (600 MHz, CD_3OD).

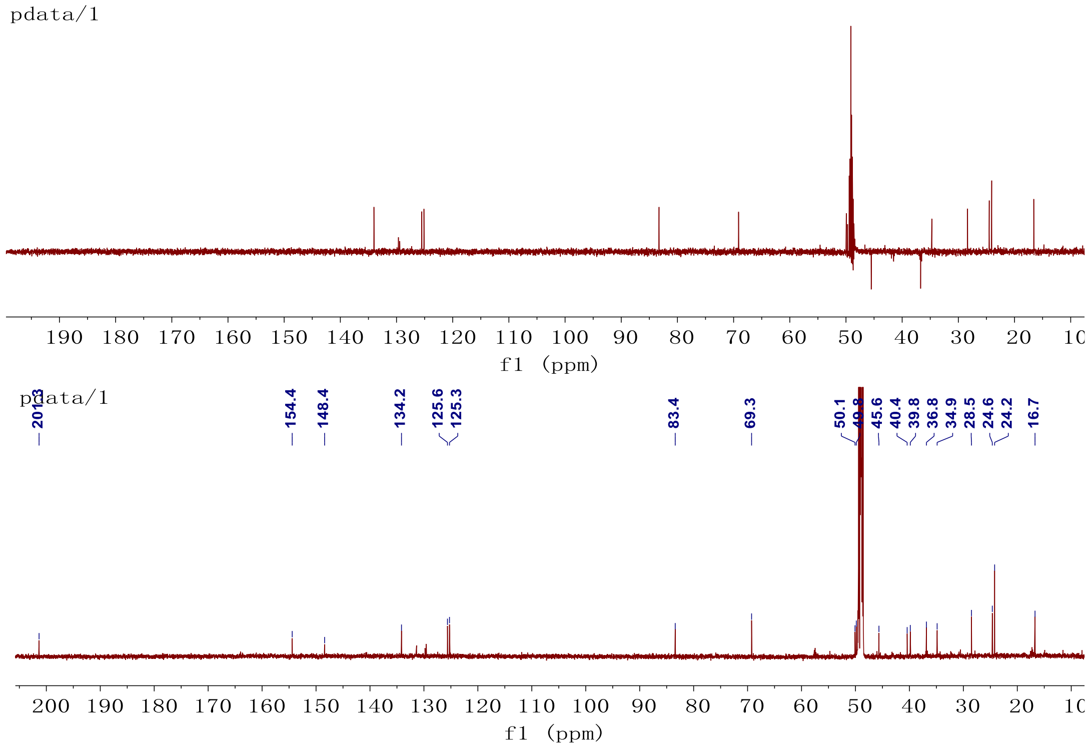

Figure S89. ^{13}C and DEPT-135 NMR spectra of compound **11** (150 MHz, CD_3OD).

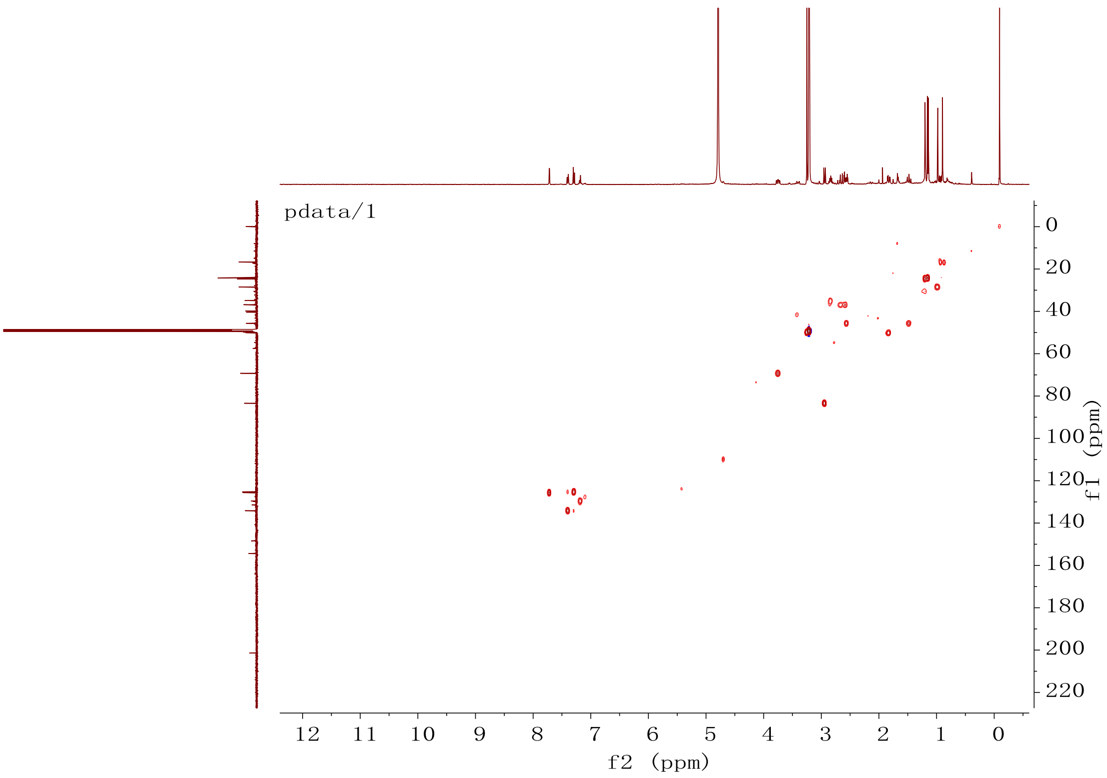

Figure S90. HSQC spectrum of compound **11** (CD_3OD).

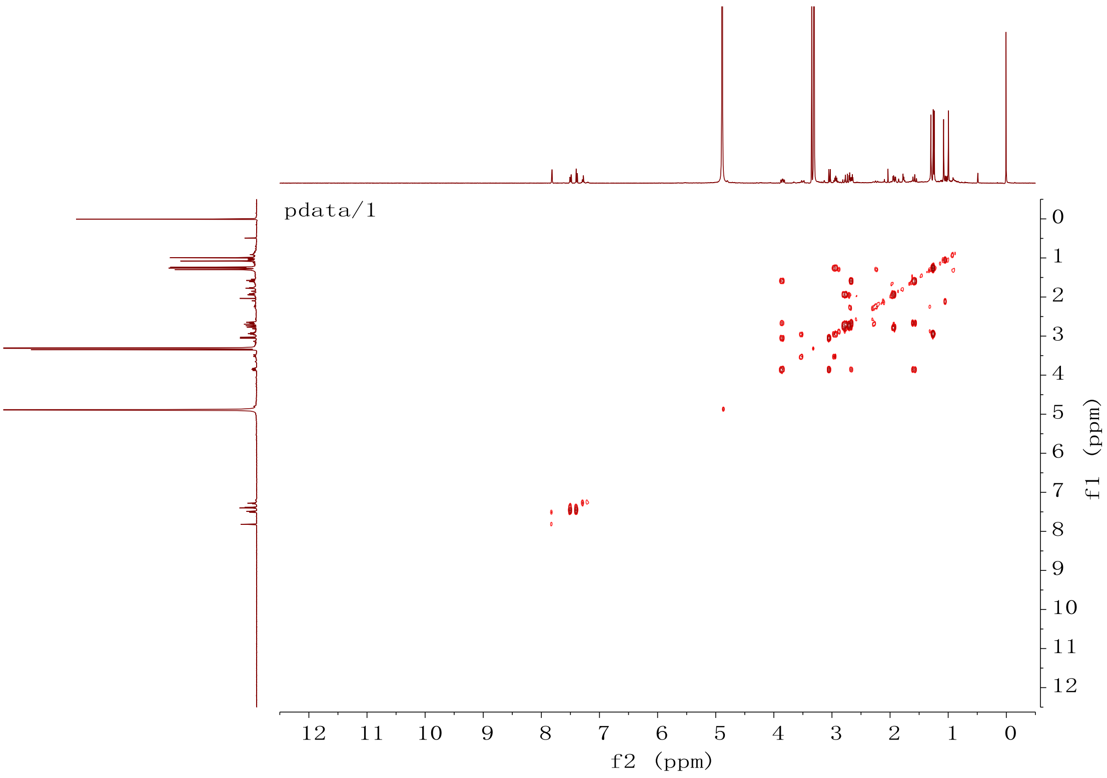

Figure S91. ${}^1H$–${}^1H$ COSY spectrum of compound **11** (CD_3OD).

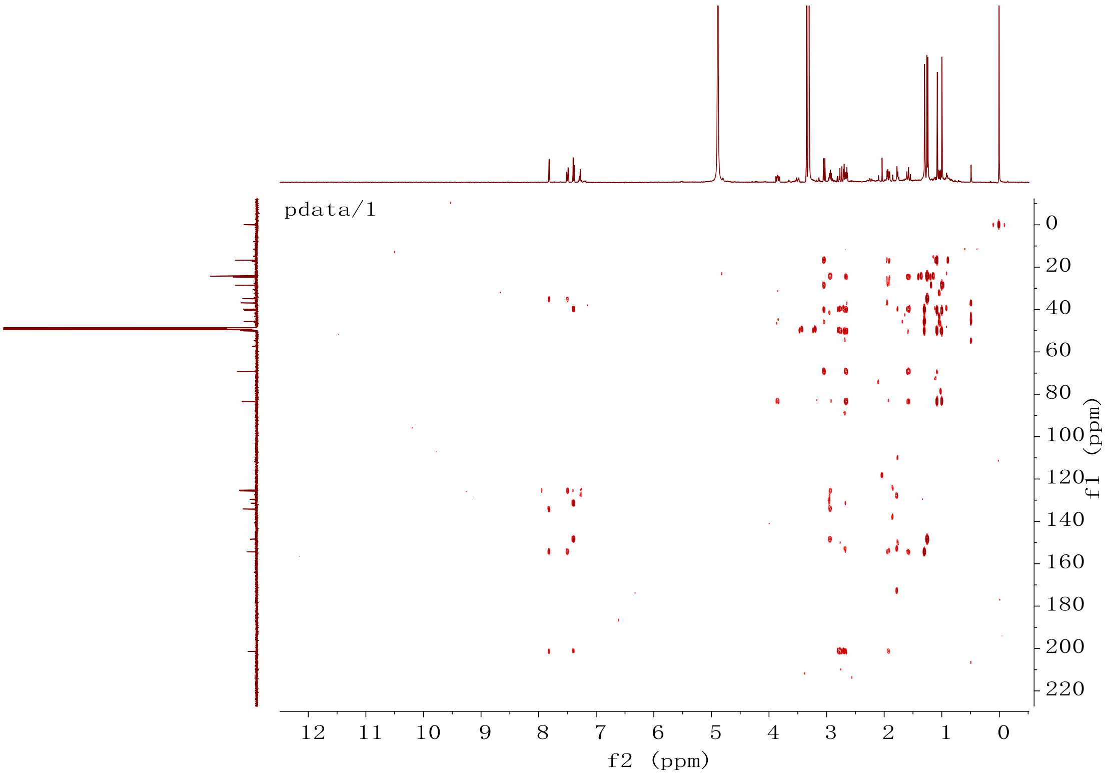

Figure S92. HMBC spectrum of compound **11** (CD_3OD).

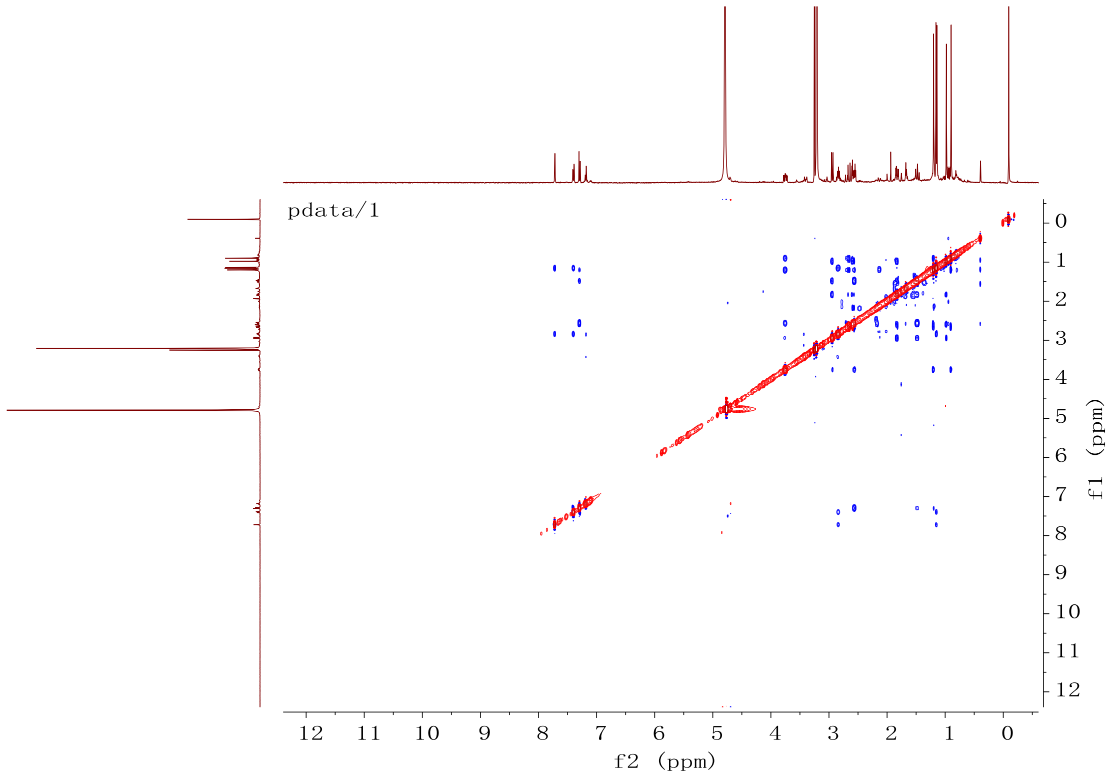

Figure S93. NOESY spectrum of compound **11** (CD_3OD).

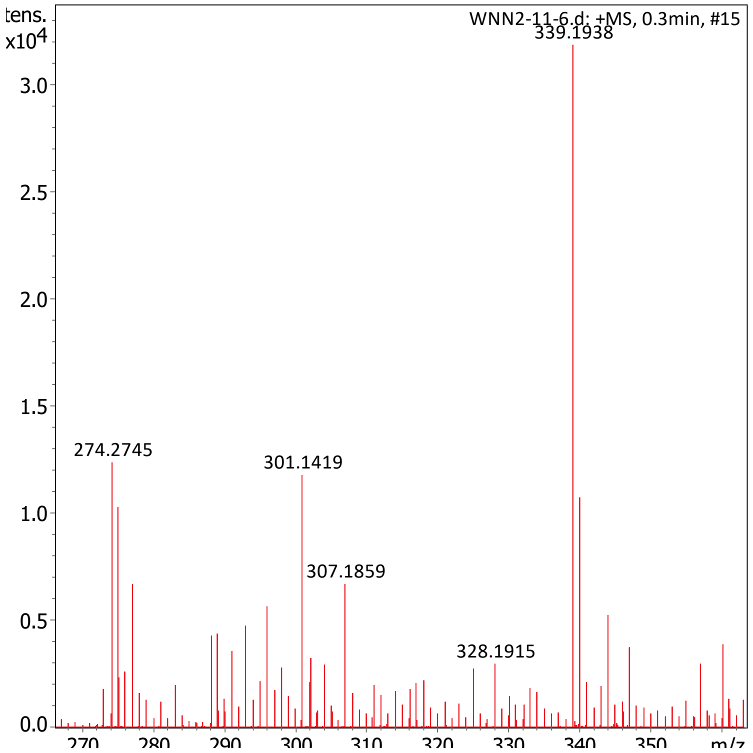

Figure S94. HRESIMS spectrum of compound **11**.

^{13}C NMR Calculation Data of 10

In general, conformational analyses were carried out via random searching in the Sybyl-X 2.0 using the MMFF94S force field with an energy cutoff of 5.0 kcal/mol [1]. The results showed eighteen lowest energy conformers. Subsequently, the conformers were re-optimized at the B3LYP-D3(BJ)/6-31G* level in polarizable continuum model (PCM) chloroform by the GAUSSIAN09 program [2]. All conformers used for property calculations in this work were characterized to be stable point on potential energy surface (PES) with no imaginary frequencies. NMR shielding constants were computed using the gauge-independent atomic orbital (GIAO) method at the mPW1PW91/6-311+G** level in PCM chloroform by the GAUSSIAN09 program [2]. Gibbs free energies for conformers were determined by using thermal correction at B3LYP-D3(BJ)/6-31G* level and electronic energies evaluated at the wB97M-V/def2-TZVP level in conductor-like polarizable continuum model (CPCM) chloroform using ORCA [3, 4]. Boltzmann weights were computed using relative Gibbs free energies [5]. The unscaled chemical shifts (δ_u) were computed using Tetramethylsilane (TMS) as a reference standard according to $\delta_u = \sigma_0 - \sigma_x$, where σ_x is the Boltzmann averaged shielding tensor (over all significantly populated conformations) and σ_0 is the shielding tensor of the TMS computed at the same level of theory employed for σ_x. The scaled chemical shifts (δ_s) were calculated as $\delta_s = (\delta_u - b) / m$, where m and b are the slope and intercept, respectively, deduced from a linear regression calculation on a plot of δ_u against δ_{exp}. The DP4+ calculations were run by the Excel spreadsheet available for free at sarotti-nmr.weebly.com or as part of the Supporting Information of the original paper [5]. Finally, we identified **10B** was the most likely structure.

References:

[1]. Sybyl Software, version X 2.0; Tripos Associates Inc.: St. Louis, MO, 2013.

[2].Gaussian 09, Revision E.01, M. J. Frisch, G. W. Trucks, H. B. Schlegel, Gaussian, Inc., Wallingford CT, 2009.

[3]. Neese, F. (2012) The ORCA program system, Wiley Interdiscip. Rev.: Comput. Mol. Sci., 2, 73-78

[4]. Neese, F. (2017) Software update: the ORCA program system, version 4.0, Wiley Interdiscip. Rev.: Comput. Mol. Sci., 8, e1327.

[5]. Nicolás Grimblat, María M. Zanardi, and Ariel M. Sarotti J. Org. Chem. 2015, 80, 12526−12534

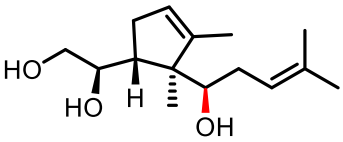

10A(0.00%)

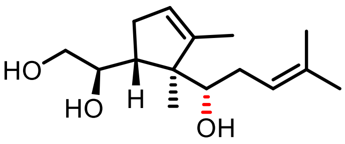

10B(100.00%)

Table S1. Gibbs free energies[a] and equilibrium populations[b] of low-energy conformers of **10A**.

Conformers	ΔG(a.u.)	P(%)/100	G(a.u.)
10A000001.out	0.00125	9.31	-812.526044
10A000003.out	0.00753	0.01	-812.519768
10A000004.out	0.00134	8.5	-812.525959
10A000005.out	0.00265	2.11	-812.524641
10A000006.out	0.01404	0.0	-812.513251
10A000007.out	0.00842	0.01	-812.518875
10A000008.out	0.01553	0.0	-812.511762
10A000009.out	0.00753	0.01	-812.519769
10A000011.out	0.00234	2.93	-812.524952
10A000012.out	0.01199	0.0	-812.515304
10A000013.out	0.00744	0.01	-812.519854
10A000014.out	0.00395	0.53	-812.523346
10A000015.out	0.0158	0.0	-812.511495
10A000016.out	0.0058	0.07	-812.521498
10A000017.out	0.00052	20.25	-812.526778
10A000018.out	0.00438	0.34	-812.52291
10A000019.out	0.00049	20.88	-812.526807
10A000020.out	0.0	35.03	-812.527295

[a]wB97M-V/def2-TZVP, in a.u.

[b]From ΔG values at 298.15K.

Table S2. Cartesian coordinates for the low-energy reoptimized random reseach conformers of **10A** at B3LYP-D3(BJ)/6-31G* level of theory in chloroform.

10A000001_en_		Standard Orientation (A.U.)			
Center number	Atomic number	Atomic Type	X	Y	Z
0	6	0	2.17279	-2.432459	-0.5783
1	6	0	-0.781063	-2.595832	-0.37961
2	6	0	-1.07612	-4.713854	1.577173
3	6	0	1.038781	-6.097062	1.750342
4	6	0	3.10273	-5.124705	0.055274
5	6	0	3.2739	-1.321219	-2.988479
6	6	0	6.151809	-1.4219	-3.061131
7	8	0	6.890712	-0.017864	-5.226228
8	8	0	2.545123	1.26627	-3.164254
9	6	0	-2.105599	-0.102669	0.456628
10	6	0	-0.928364	1.102385	2.840716
11	6	0	-2.52996	3.185502	3.910276
12	6	0	-2.427896	5.645215	3.28754
13	6	0	-4.225564	7.531203	4.435301
14	6	0	-0.597885	6.737654	1.401277
15	6	0	-1.993355	-3.492943	-2.871841
16	6	0	-3.515614	-5.223449	2.913926
17	8	0	-2.318057	1.665153	-1.519187
18	1	0	2.819672	-1.209168	0.97371
19	1	0	1.235503	-7.795211	2.909763
20	1	0	4.981324	-5.124363	0.957751
21	1	0	3.277473	-6.311239	-1.661976
22	1	0	2.56525	-2.363923	-4.657302
23	1	0	6.910449	-0.57198	-1.306649
24	1	0	6.807699	-3.400806	-3.173162
25	1	0	8.677254	0.330373	-5.141648
26	1	0	3.61052	2.006946	-4.46201
27	1	0	-4.070763	-0.620586	0.92558
28	1	0	-0.63566	-0.378998	4.27755
29	1	0	0.958312	1.823537	2.348559
30	1	0	-3.987594	2.602244	5.265019
31	1	0	-3.192311	9.047442	5.432481
32	1	0	-5.360424	8.47134	2.956265

33	1	0	-5.533148	6.640938	5.784197
34	1	0	-1.636138	7.452974	-0.260177
35	1	0	0.4263	8.362193	2.219474
36	1	0	0.783462	5.358768	0.705533
37	1	0	-1.088898	-5.233126	-3.564666
38	1	0	-4.013601	-3.898205	-2.585244
39	1	0	-1.872779	-2.024711	-4.3316
40	1	0	-5.093018	-5.423559	1.565636
41	1	0	-3.405251	-6.967638	4.039008
42	1	0	-4.03216	-3.665405	4.19834
43	1	0	-0.636244	1.895274	-2.239454

10A000003_en_		Standard Orientation (A.U.)			
Center number	Atomic number	Atomic Type	X	Y	Z
0	6	0	3.252468	-2.268318	0.464788
1	6	0	0.507756	-2.251187	-0.668629
2	6	0	-0.884171	-4.063321	1.125697
3	6	0	0.749341	-5.383444	2.545323
4	6	0	3.458318	-4.724758	2.022693
5	6	0	5.422291	-1.885276	-1.418156
6	6	0	7.980527	-1.617422	-0.085775
7	8	0	8.118641	0.731683	1.204203
8	8	0	5.056626	0.273298	-2.962218
9	6	0	-0.500182	0.511488	-0.578523
10	6	0	-3.020425	1.029211	-1.950037
11	6	0	-3.617414	3.805432	-2.035299
12	6	0	-4.954654	5.132857	-0.334155
13	6	0	-5.301041	7.945934	-0.600706
14	6	0	-6.152774	4.000322	1.985163
15	6	0	0.429042	-3.331387	-3.366634
16	6	0	-3.692867	-4.459591	1.116282
17	8	0	-0.586174	1.391638	1.959686
18	1	0	3.279246	-0.675588	1.806136
19	1	0	0.192624	-6.87862	3.859631
20	1	0	4.593083	-4.490431	3.756434
21	1	0	4.383337	-6.255326	0.932612
22	1	0	5.519401	-3.532314	-2.692036
23	1	0	8.352204	-3.232994	1.184051
24	1	0	9.485773	-1.570175	-1.519565
25	1	0	6.9933	0.667355	2.641877

26	1	0	5.695478	1.686233	-1.979978
27	1	0	0.948835	1.669671	-1.515322
28	1	0	-2.878726	0.308267	-3.894786
29	1	0	-4.554542	-0.028216	-1.035101
30	1	0	-2.770635	4.852255	-3.611403
31	1	0	-7.324278	8.449909	-0.709165
32	1	0	-4.535819	8.947662	1.063755
33	1	0	-4.353045	8.690403	-2.293984
34	1	0	-6.142838	1.926413	1.973993
35	1	0	-5.12764	4.618322	3.693296
36	1	0	-8.126112	4.643161	2.19465
37	1	0	1.354716	-2.04071	-4.705044
38	1	0	1.41039	-5.163401	-3.439799
39	1	0	-1.520897	-3.65519	-4.00584
40	1	0	-4.412503	-4.778995	-0.811649
41	1	0	-4.212914	-6.101723	2.279609
42	1	0	-4.729399	-2.81141	1.858873
43	1	0	-1.365983	0.08784	2.976687

10A000004_en_		Standard Orientation (A.U.)			
Center number	Atomic number	Atomic Type	X	Y	Z
0	6	0	2.255082	-2.42381	-0.593091
1	6	0	-0.703912	-2.531211	-0.440476
2	6	0	-1.070381	-4.686715	1.462106
3	6	0	1.01346	-6.116626	1.633698
4	6	0	3.121618	-5.148103	-0.00846
5	6	0	3.414782	-1.278262	-2.959434
6	6	0	6.290626	-1.440816	-2.995605
7	8	0	7.089508	-0.001708	-5.115946
8	8	0	2.745759	1.328426	-3.080979
9	6	0	-1.989628	-0.030043	0.433811
10	6	0	-0.827466	1.09434	2.864585
11	6	0	-2.394381	3.198944	3.944253
12	6	0	-2.216469	5.665788	3.367964
13	6	0	-3.985276	7.578677	4.515897
14	6	0	-0.323848	6.74167	1.534134
15	6	0	-1.898725	-3.345233	-2.969058
16	6	0	-3.540338	-5.175892	2.749843
17	8	0	-2.13214	1.786082	-1.50385
18	1	0	2.90308	-1.251605	0.997413

19	1	0	1.157217	-7.844888	2.755837
20	1	0	4.986101	-5.205304	0.920988
21	1	0	3.298948	-6.299136	-1.74945
22	1	0	2.707318	-2.264225	-4.663005
23	1	0	7.042784	-0.65016	-1.210863
24	1	0	6.904976	-3.430497	-3.146273
25	1	0	8.880481	0.311542	-4.995296
26	1	0	3.845264	2.076257	-4.345792
27	1	0	-3.972601	-0.516268	0.860175
28	1	0	-0.604975	-0.423193	4.275851
29	1	0	1.08674	1.774787	2.424418
30	1	0	-3.892201	2.630918	5.260988
31	1	0	-2.932008	9.044896	5.565708
32	1	0	-5.061114	8.579461	3.032527
33	1	0	-5.346326	6.699497	5.818471
34	1	0	-1.311982	7.500659	-0.138396
35	1	0	0.720585	8.331032	2.39458
36	1	0	1.040054	5.340693	0.848185
37	1	0	-1.018668	-5.084903	-3.694252
38	1	0	-3.929629	-3.71945	-2.717545
39	1	0	-1.733157	-1.844259	-4.390609
40	1	0	-5.101062	-5.303868	1.373729
41	1	0	-3.486322	-6.950783	3.83011
42	1	0	-4.040671	-3.639955	4.066951
43	1	0	-0.433097	1.999149	-2.188059

10A000005_en_		Standard Orientation (A.U.)			
Center number	Atomic number	Atomic Type	X	Y	Z
0	6	0	1.845212	-1.461622	0.151324
1	6	0	-0.606819	-3.051433	-0.337019
2	6	0	-0.97289	-4.369215	2.221277
3	6	0	1.148483	-4.278312	3.600079
4	6	0	3.233987	-2.863158	2.300651
5	6	0	3.530847	-0.830584	-2.114339
6	6	0	5.790212	0.803204	-1.331282
7	8	0	4.945088	3.16587	-0.428354
8	8	0	2.272307	0.645442	-3.962559
9	6	0	-2.973953	-1.478304	-1.014008
10	6	0	-3.63332	0.695878	0.831634
11	6	0	-2.406491	3.208715	0.309135

12	6	0	-0.839575	4.536709	1.804794
13	6	0	0.178086	7.064131	0.980055
14	6	0	0.148451	3.640244	4.315968
15	6	0	-0.210383	-5.097457	-2.377856
16	6	0	-3.353686	-5.735069	2.91437
17	8	0	-2.697503	-0.593493	-3.555176
18	1	0	1.24868	0.357653	0.921559
19	1	0	1.38107	-5.210328	5.42948
20	1	0	4.240292	-1.546038	3.561518
21	1	0	4.679888	-4.188975	1.566077
22	1	0	4.265666	-2.604027	-2.95775
23	1	0	6.90663	-0.107459	0.167228
24	1	0	7.032593	1.031426	-3.001305
25	1	0	3.611832	3.633839	-1.602913
26	1	0	0.471602	0.24279	-3.999031
27	1	0	-4.576159	-2.817245	-0.98823
28	1	0	-5.704441	0.968687	0.736974
29	1	0	-3.237292	0.046096	2.763524
30	1	0	-2.894597	4.063334	-1.513603
31	1	0	-0.362816	8.561227	2.32966
32	1	0	2.261877	7.024961	0.938213
33	1	0	-0.514349	7.609081	-0.903019
34	1	0	2.214134	3.394271	4.166194
35	1	0	-0.190422	5.065333	5.79973
36	1	0	-0.672892	1.840911	4.940961
37	1	0	-1.88219	-6.327867	-2.525902
38	1	0	0.114837	-4.256015	-4.245941
39	1	0	1.419649	-6.292818	-1.894853
40	1	0	-4.964235	-4.431733	3.151283
41	1	0	-3.906875	-7.098764	1.437556
42	1	0	-3.122784	-6.780024	4.695966
43	1	0	-4.101594	0.490297	-3.983828

10A000006_en_		Standard Orientation (A.U.)			
Center number	Atomic number	Atomic Type	X	Y	Z
0	6	0	3.716577	-2.092775	0.302829
1	6	0	0.949311	-2.049068	-0.796729
2	6	0	-0.273069	-4.286961	0.605481
3	6	0	1.469217	-5.719823	1.763619
4	6	0	4.111262	-4.773853	1.384357

5	6	0	5.956617	-1.211568	-1.321622
6	6	0	5.798821	1.514319	-2.24419
7	8	0	5.563293	3.102443	-0.113879
8	8	0	6.40102	-2.689643	-3.513441
9	6	0	-0.285139	0.525831	-0.059659
10	6	0	-2.805049	1.184017	-1.37545
11	6	0	-3.67149	3.808987	-0.719929
12	6	0	-5.18298	4.484433	1.204398
13	6	0	-5.796519	7.218423	1.707581
14	6	0	-6.329719	2.645292	3.047525
15	6	0	0.783976	-2.56132	-3.654789
16	6	0	-3.044799	-4.886044	0.521398
17	8	0	-0.539615	0.705979	2.607361
18	1	0	3.681748	-0.796196	1.925346
19	1	0	1.03567	-7.470518	2.773423
20	1	0	5.25493	-4.760762	3.127313
21	1	0	5.120092	-6.038011	0.04953
22	1	0	7.628717	-1.288383	-0.056998
23	1	0	7.538611	1.909859	-3.334533
24	1	0	4.187624	1.718213	-3.557198
25	1	0	5.275715	4.811058	-0.676796
26	1	0	6.746421	-4.400384	-2.988347
27	1	0	1.069269	2.01956	-0.572266
28	1	0	-2.536067	1.06195	-3.433784
29	1	0	-4.250094	-0.219416	-0.877623
30	1	0	-2.889671	5.327875	-1.895577
31	1	0	-4.874265	8.488827	0.344805
32	1	0	-7.85535	7.555875	1.624739
33	1	0	-5.188415	7.773449	3.626254
34	1	0	-5.373391	2.797248	4.894311
35	1	0	-8.34785	3.06682	3.363253
36	1	0	-6.162316	0.672402	2.431541
37	1	0	-1.182281	-2.893997	-4.238214
38	1	0	1.514404	-0.972423	-4.773773
39	1	0	1.903708	-4.228149	-4.16706
40	1	0	-3.764871	-4.910877	-1.432282
41	1	0	-3.424324	-6.745496	1.369364
42	1	0	-4.182497	-3.477332	1.55049
43	1	0	-0.750059	-0.985902	3.267803

10A000007_en_			Standard Orientation (A.U.)		
Center number	Atomic number	Atomic Type	X	Y	Z
0	6	0	1.726558	-1.278455	-1.010314
1	6	0	-0.83671	-2.660706	-0.35628
2	6	0	-0.063627	-4.533011	1.703204
3	6	0	2.44894	-4.611402	1.993153
4	6	0	3.832582	-2.923306	0.187948
5	6	0	2.179912	-0.625913	-3.801785
6	6	0	4.726965	0.690572	-4.263243
7	8	0	6.856512	-0.91153	-4.135511
8	8	0	0.346549	1.045174	-4.784889
9	6	0	-2.904757	-0.802814	0.613621
10	6	0	-2.532545	0.31727	3.292997
11	6	0	-0.522141	2.316844	3.502959
12	6	0	-0.833076	4.834985	3.262901
13	6	0	1.381851	6.60643	3.511659
14	6	0	-3.33506	6.11494	2.785933
15	6	0	-1.926863	-4.14131	-2.619712
16	6	0	-1.942582	-6.189815	3.02859
17	8	0	-3.313986	1.198731	-1.148901
18	1	0	1.719294	0.547149	-0.020116
19	1	0	3.419335	-5.874021	3.311388
20	1	0	5.312075	-1.765673	1.087628
21	1	0	4.839407	-4.048774	-1.249256
22	1	0	2.209657	-2.410877	-4.914138
23	1	0	4.574407	1.648028	-6.115467
24	1	0	5.006971	2.178552	-2.832448
25	1	0	6.729366	-2.121856	-5.496115
26	1	0	-1.226251	0.864582	-3.843864
27	1	0	-4.694768	-1.866632	0.658793
28	1	0	-4.372367	1.095104	3.871522
29	1	0	-2.094134	-1.226835	4.608343
30	1	0	1.392233	1.634573	3.887673
31	1	0	1.629265	7.73553	1.774774
32	1	0	1.072036	7.970254	5.060647
33	1	0	3.154998	5.588704	3.882767
34	1	0	-3.220849	7.341786	1.102795
35	1	0	-4.899988	4.788097	2.490999
36	1	0	-3.820108	7.36715	4.383318
37	1	0	-0.535747	-5.503499	-3.346714
38	1	0	-3.620392	-5.207258	-2.052836

39	1	0	-2.471792	-2.884521	-4.179672
40	1	0	-2.849425	-7.511535	1.694892
41	1	0	-1.024575	-7.321767	4.510406
42	1	0	-3.483527	-5.102737	3.914415
43	1	0	-2.200051	2.570353	-0.647997

10A000008_en_		Standard Orientation (A.U.)			
Center number	Atomic number	Atomic Type	X	Y	Z
0	6	0	3.378095	-2.408106	0.096726
1	6	0	0.529781	-2.084466	-0.678504
2	6	0	-0.26734	-4.887249	-0.848568
3	6	0	1.766166	-6.368715	-1.178053
4	6	0	4.194245	-4.902239	-1.153237
5	6	0	5.278258	-0.23905	-0.167562
6	6	0	4.701293	2.053622	1.517628
7	8	0	4.246691	1.426428	4.054859
8	8	0	5.551076	0.728017	-2.651679
9	6	0	-0.891913	-0.494439	1.331316
10	6	0	-3.721375	0.112134	0.895798
11	6	0	-4.194077	2.258034	-0.919945
12	6	0	-3.740891	4.716312	-0.462987
13	6	0	-4.204629	6.699903	-2.450242
14	6	0	-2.709334	5.700572	2.0029
15	6	0	0.192856	-0.90221	-3.318221
16	6	0	-2.945771	-5.808742	-0.94927
17	8	0	-0.512936	-1.503432	3.81013
18	1	0	3.351898	-2.823931	2.131906
19	1	0	1.669297	-8.401433	-1.536121
20	1	0	5.738603	-5.836533	-0.112539
21	1	0	4.890944	-4.620394	-3.110752
22	1	0	7.104068	-1.023646	0.505428
23	1	0	6.361875	3.306246	1.475691
24	1	0	3.134657	3.134487	0.646114
25	1	0	2.695522	0.452191	4.208099
26	1	0	6.100478	-0.624435	-3.743651
27	1	0	0.036693	1.354263	1.390982
28	1	0	-4.775051	-1.565812	0.291989
29	1	0	-4.444511	0.605701	2.783846
30	1	0	-4.927497	1.756729	-2.788
31	1	0	-5.578674	8.122623	-1.783401
32	1	0	-2.44725	7.739732	-2.883913
33	1	0	-4.922771	5.878246	-4.219146
34	1	0	-2.678216	4.276847	3.51219
35	1	0	-0.757068	6.398441	1.758549
36	1	0	-3.832499	7.325525	2.671324
37	1	0	-1.765751	-1.115411	-3.976109
38	1	0	0.667784	1.115188	-3.325606

39	1	0	1.419097	-1.852907	-4.698284
40	1	0	-3.003243	-7.843108	-1.373028
41	1	0	-3.95925	-5.51231	0.847242
42	1	0	-4.043266	-4.818501	-2.417956
43	1	0	-0.669354	-3.324675	3.736472

10A000009_en_		Standard Orientation (A.U.)			
Center number	Atomic number	Atomic Type	X	Y	Z
0	6	0	3.3213	-2.344642	0.616412
1	6	0	0.384794	-2.306796	0.149162
2	6	0	-0.546641	-4.234357	2.116353
3	6	0	1.377608	-5.608671	3.030499
4	6	0	3.883365	-4.891112	1.917809
5	6	0	5.013593	-1.79558	-1.660444
6	6	0	7.829014	-1.866448	-0.990251
7	8	0	8.476408	-0.041752	0.853087
8	8	0	4.435151	0.582897	-2.792976
9	6	0	-0.616039	0.416443	0.62367
10	6	0	-3.385427	0.94755	-0.105587
11	6	0	-4.026071	3.689709	0.241724
12	6	0	-4.98111	5.303556	-1.472252
13	6	0	-5.474278	8.026453	-0.792358
14	6	0	-5.653509	4.620256	-4.153488
15	6	0	-0.31076	-3.235912	-2.515728
16	6	0	-3.276577	-4.668062	2.754055
17	8	0	-0.12877	1.175187	3.153964
18	1	0	3.708862	-0.859793	2.021689
19	1	0	1.146145	-7.179247	4.354343
20	1	0	5.406677	-4.738435	3.330089
21	1	0	4.502949	-6.346836	0.545564
22	1	0	4.691801	-3.215318	-3.151392
23	1	0	8.344539	-3.724289	-0.210531
24	1	0	8.926766	-1.596514	-2.749058
25	1	0	8.461262	1.591597	0.039476
26	1	0	4.264159	1.798641	-1.439588
27	1	0	0.531468	1.675972	-0.569696
28	1	0	-3.699823	0.35172	-2.067073
29	1	0	-4.647494	-0.208025	1.081434
30	1	0	-3.599231	4.421411	2.129888
31	1	0	-4.952216	8.440396	1.176031

32	1	0	-4.400356	9.314362	-2.036403
33	1	0	-7.489041	8.511136	-1.049526
34	1	0	-7.680066	4.968321	-4.512694
35	1	0	-4.607215	5.822318	-5.500976
36	1	0	-5.256632	2.639583	-4.626475
37	1	0	0.266126	-1.850274	-3.953706
38	1	0	0.640175	-5.039096	-2.926386
39	1	0	-2.353225	-3.564322	-2.706415
40	1	0	-4.436126	-4.901934	1.039563
41	1	0	-3.498255	-6.375883	3.918094
42	1	0	-4.105683	-3.074388	3.80927
43	1	0	-0.407854	-0.272984	4.235634

10A000011_en_		Standard Orientation (A.U.)			
Center number	Atomic number	Atomic Type	X	Y	Z
0	6	0	2.577431	-1.711083	0.365787
1	6	0	-0.199619	-2.10038	-0.582863
2	6	0	-1.64762	-2.354846	1.920412
3	6	0	-0.060165	-2.911941	3.813197
4	6	0	2.649288	-3.01653	2.972976
5	6	0	4.722881	-2.466699	-1.422174
6	6	0	7.323577	-1.991454	-0.249669
7	8	0	7.678125	0.602819	0.229575
8	8	0	4.737273	-1.001243	-3.66674
9	6	0	-1.222354	0.066214	-2.280272
10	6	0	-0.619333	2.779156	-1.447645
11	6	0	-1.397305	3.44146	1.201259
12	6	0	-3.602375	4.474097	1.925604
13	6	0	-4.14965	4.976633	4.67276
14	6	0	-5.710055	5.161664	0.140221
15	6	0	-0.516041	-4.632134	-2.005772
16	6	0	-4.470092	-2.263161	2.081989
17	8	0	-0.247017	-0.10792	-4.810825
18	1	0	2.841264	0.313831	0.732301
19	1	0	-0.66291	-3.337358	5.742899
20	1	0	3.962546	-2.063556	4.277546
21	1	0	3.303447	-5.001742	2.83042
22	1	0	4.582163	-4.512874	-1.86129
23	1	0	7.533363	-3.014722	1.547988
24	1	0	8.76872	-2.733185	-1.572509

25	1	0	7.154702	1.426579	-1.323853
26	1	0	3.016392	-0.707164	-4.269783
27	1	0	-3.297963	-0.143039	-2.371427
28	1	0	1.428867	3.057664	-1.682805
29	1	0	-1.529248	4.027398	-2.83793
30	1	0	-0.033766	2.978571	2.684118
31	1	0	-5.841177	3.922465	5.295835
32	1	0	-2.560184	4.426965	5.892799
33	1	0	-4.562559	6.995893	5.001977
34	1	0	-7.419428	4.037714	0.557987
35	1	0	-6.257632	7.160524	0.378238
36	1	0	-5.226306	4.860971	-1.855121
37	1	0	0.597858	-4.698934	-3.759878
38	1	0	0.107866	-6.216341	-0.815723
39	1	0	-2.514069	-4.970165	-2.490209
40	1	0	-5.119952	-2.746314	3.996068
41	1	0	-5.198119	-0.369262	1.620699
42	1	0	-5.35802	-3.601423	0.750919
43	1	0	-1.018245	-1.532067	-5.650117

10A000012_en_		Standard Orientation (A.U.)			
Center number	Atomic number	Atomic Type	X	Y	Z
0	6	0	3.336476	-2.21835	0.310713
1	6	0	0.430506	-1.737535	-0.101023
2	6	0	-0.612358	-2.253553	2.564067
3	6	0	1.095331	-3.452728	4.001395
4	6	0	3.551495	-3.913914	2.669714
5	6	0	4.815759	-3.227339	-1.969605
6	6	0	7.661915	-3.37048	-1.446423
7	8	0	8.711571	-0.902306	-1.332357
8	8	0	4.455876	-1.742208	-4.172967
9	6	0	0.155946	1.034548	-1.091965
10	6	0	-2.261008	1.821789	-2.541249
11	6	0	-4.661847	2.086239	-1.051081
12	6	0	-5.48085	4.157241	0.179293
13	6	0	-7.953903	4.135535	1.591142
14	6	0	-4.080433	6.6369	0.255192
15	6	0	-0.743817	-3.633698	-1.965247
16	6	0	-3.271751	-1.746999	3.394472
17	8	0	0.755235	2.804325	0.828892

18	1	0	4.108066	-0.350862	0.82322
19	1	0	0.712359	-4.129649	5.916249
20	1	0	5.217409	-3.461118	3.840328
21	1	0	3.743746	-5.935121	2.1539
22	1	0	4.138912	-5.14728	-2.417324
23	1	0	8.069199	-4.475831	0.276595
24	1	0	8.594062	-4.312803	-3.048759
25	1	0	8.134162	-0.102931	0.205606
26	1	0	5.690128	-0.389255	-4.045248
27	1	0	1.673007	1.222776	-2.493616
28	1	0	-1.79072	3.641655	-3.432259
29	1	0	-2.556008	0.481997	-4.102091
30	1	0	-5.866921	0.406667	-0.959737
31	1	0	-8.927841	2.306065	1.433025
32	1	0	-7.662524	4.54523	3.617805
33	1	0	-9.236454	5.61907	0.875988
34	1	0	-3.877338	7.295138	2.224909
35	1	0	-2.17931	6.522456	-0.559227
36	1	0	-5.162282	8.118965	-0.740975
37	1	0	-0.034916	-3.319421	-3.89242
38	1	0	-0.285694	-5.585237	-1.410129
39	1	0	-2.816068	-3.474273	-1.999126
40	1	0	-3.604074	-2.530379	5.29115
41	1	0	-3.724869	0.281985	3.442004
42	1	0	-4.655282	-2.603763	2.095071
43	1	0	-0.565638	2.72819	2.089489

10A000013_en_		Standard Orientation (A.U.)			
Center number	Atomic number	Atomic Type	X	Y	Z
0	6	0	3.058994	-2.234178	0.681329
1	6	0	0.429032	-2.241894	-0.697725
2	6	0	-1.042286	-4.207728	0.856458
3	6	0	0.509036	-5.543306	2.35191
4	6	0	3.224559	-4.761186	2.122251
5	6	0	5.37851	-1.696783	-0.938559
6	6	0	7.807844	-1.546639	0.626877
7	8	0	7.6726	0.375279	2.45298
8	8	0	5.014783	0.699004	-2.144369
9	6	0	-0.700586	0.470763	-0.531582
10	6	0	-3.114981	0.978455	-2.089557

11	6	0	-3.793455	3.737788	-2.096243
12	6	0	-5.295453	4.94705	-0.445178
13	6	0	-5.708239	7.758868	-0.614703
14	6	0	-6.628481	3.675914	1.723277
15	6	0	0.622893	-3.154849	-3.450729
16	6	0	-3.821504	-4.699239	0.571164
17	8	0	-1.059139	1.178031	2.034122
18	1	0	2.953461	-0.725213	2.102235
19	1	0	-0.104213	-7.12505	3.533096
20	1	0	4.18666	-4.558549	3.958942
21	1	0	4.30302	-6.206033	1.055571
22	1	0	5.608533	-3.201086	-2.374048
23	1	0	8.166382	-3.345282	1.610377
24	1	0	9.418924	-1.249785	-0.693212
25	1	0	6.883505	1.805732	1.622016
26	1	0	6.314578	0.910667	-3.409644
27	1	0	0.771135	1.746971	-1.255653
28	1	0	-2.795708	0.360314	-4.049284
29	1	0	-4.684794	-0.163325	-1.354456
30	1	0	-2.863988	4.881346	-3.554503
31	1	0	-7.732878	8.210435	-0.852839
32	1	0	-5.099158	8.704921	1.14398
33	1	0	-4.66203	8.605694	-2.198766
34	1	0	-5.759857	4.251848	3.529691
35	1	0	-8.634641	4.242579	1.801342
36	1	0	-6.534877	1.606674	1.630097
37	1	0	1.590006	-1.743467	-4.630766
38	1	0	1.69129	-4.936653	-3.550703
39	1	0	-1.248739	-3.515818	-4.27584
40	1	0	-4.35991	-4.919796	-1.428459
41	1	0	-4.373446	-6.429565	1.582232
42	1	0	-4.981295	-3.138461	1.31897
43	1	0	-1.737373	-0.265917	2.927078

10A000014_en_		Standard Orientation (A.U.)			
Center number	Atomic number	Atomic Type	X	Y	Z
0	6	0	2.559594	-1.994151	-1.234059
1	6	0	-0.294034	-1.313505	-1.662515
2	6	0	-1.614344	-3.490874	-0.27015
3	6	0	-0.031154	-5.44706	0.013521
4	6	0	2.553853	-4.907955	-1.0293
5	6	0	4.486739	-0.830136	-3.056779
6	6	0	7.169271	-1.807073	-2.640909
7	8	0	7.393291	-4.280048	-3.628036
8	8	0	4.665624	1.814249	-2.752037
9	6	0	-1.140678	1.281111	-0.604624
10	6	0	-0.316733	1.801918	2.118168
11	6	0	-1.757398	3.944618	3.288333
12	6	0	-3.296671	3.863723	5.308231
13	6	0	-4.640039	6.205526	6.217547
14	6	0	-3.834825	1.525795	6.843526
15	6	0	-1.038055	-1.467869	-4.4785
16	6	0	-4.351648	-3.418696	0.445688
17	8	0	-0.230329	3.359105	-2.096959
18	1	0	3.069588	-1.283345	0.654306
19	1	0	-0.573477	-7.270071	0.821848
20	1	0	4.107017	-5.614306	0.159827
21	1	0	2.829296	-5.812811	-2.890217
22	1	0	3.983686	-1.346133	-5.026433
23	1	0	8.446664	-0.463559	-3.606072
24	1	0	7.615551	-1.731664	-0.594517
25	1	0	9.0864	-4.873619	-3.31129
26	1	0	2.975943	2.549879	-2.679033
27	1	0	-3.225933	1.321625	-0.673644
28	1	0	-0.499213	0.052281	3.219077
29	1	0	1.713864	2.270001	2.057135
30	1	0	-1.532626	5.757669	2.313607
31	1	0	-6.710015	5.932731	6.208064
32	1	0	-4.118373	6.648542	8.189869
33	1	0	-4.197327	7.861336	5.042369
34	1	0	-5.879148	1.105046	6.84246
35	1	0	-2.837229	-0.160655	6.162456
36	1	0	-3.302315	1.818899	8.839933
37	1	0	-3.099523	-1.275601	-4.709075
38	1	0	-0.119028	0.01432	-5.611483

39	1	0	-0.483798	-3.29895	-5.286766
40	1	0	-5.561781	-2.956841	-1.189641
41	1	0	-4.974585	-5.253138	1.198491
42	1	0	-4.741215	-1.971806	1.895951
43	1	0	-1.210319	3.465425	-3.631366

10A000015_en_		Standard Orientation (A.U.)			
Center number	Atomic number	Atomic Type	X	Y	Z
0	6	0	3.050902	-2.269777	0.642906
1	6	0	0.390559	-2.358681	-0.68498
2	6	0	0.014379	-5.215432	-1.044351
3	6	0	2.187394	-6.475426	-0.704501
4	6	0	4.364933	-4.77637	-0.052867
5	6	0	4.784155	0.036651	0.270592
6	6	0	3.660932	2.588226	1.081721
7	8	0	2.83359	2.629512	3.622769
8	8	0	5.816981	0.247775	-2.19222
9	6	0	-1.6237	-1.191191	1.136557
10	6	0	-4.078989	-0.18075	-0.050684
11	6	0	-3.825465	2.396866	-1.241879
12	6	0	-3.470183	4.581641	0.010488
13	6	0	-3.138871	7.043639	-1.379253
14	6	0	-3.326096	4.762238	2.849934
15	6	0	0.382163	-1.129578	-3.325372
16	6	0	-2.459781	-6.34437	-1.824306
17	8	0	-2.364837	-2.980119	3.007121
18	1	0	2.66325	-2.321604	2.69141
19	1	0	2.390076	-8.514361	-0.97466
20	1	0	5.554143	-5.499392	1.500429
21	1	0	5.631095	-4.509739	-1.691515
22	1	0	6.443562	-0.302897	1.497727
23	1	0	5.096973	4.058608	0.700839
24	1	0	1.989507	3.053461	-0.065453
25	1	0	4.280082	2.402074	4.713584
26	1	0	4.4412	0.49788	-3.361524
27	1	0	-0.718275	0.41542	2.09728
28	1	0	-4.84474	-1.542349	-1.420755
29	1	0	-5.444951	-0.117632	1.520079
30	1	0	-3.901198	2.486868	-3.307763
31	1	0	-1.312591	7.930109	-0.890008

32	1	0	-3.206253	6.793696	-3.442138
33	1	0	-4.615872	8.415447	-0.83795
34	1	0	-1.350587	4.987641	3.479585
35	1	0	-4.384168	6.423807	3.531849
36	1	0	-4.080522	3.078443	3.801269
37	1	0	-1.420658	-1.463181	-4.299478
38	1	0	0.633117	0.930845	-3.24671
39	1	0	1.868425	-1.973299	-4.511054
40	1	0	-2.282268	-8.392635	-2.133028
41	1	0	-3.897781	-6.012096	-0.359082
42	1	0	-3.170356	-5.494326	-3.590731
43	1	0	-0.914153	-4.001826	3.439294

10A000016_en_		Standard Orientation (A.U.)			
Center number	Atomic number	Atomic Type	X	Y	Z
0	6	0	1.791234	-1.58483	-0.81242
1	6	0	-0.798625	-2.877465	-0.11256
2	6	0	-0.082628	-4.628079	2.070775
3	6	0	2.426454	-4.752627	2.38028
4	6	0	3.855056	-3.208317	0.479172
5	6	0	2.316959	-1.132632	-3.619011
6	6	0	4.872143	0.156551	-4.047914
7	8	0	5.161675	0.836697	-6.594887
8	8	0	0.519495	0.472069	-4.800159
9	6	0	-2.843048	-0.916833	0.688652
10	6	0	-2.509059	0.364621	3.302068
11	6	0	-0.527398	2.401509	3.401199
12	6	0	-0.870997	4.895145	2.9982
13	6	0	1.317559	6.710221	3.146264
14	6	0	-3.38789	6.108404	2.432219
15	6	0	-1.881489	-4.482342	-2.29374
16	6	0	-2.009648	-6.153952	3.480955
17	8	0	-3.146403	0.979643	-1.208533
18	1	0	1.817958	0.28766	0.084839
19	1	0	3.361734	-5.956379	3.77573
20	1	0	5.363098	-2.042399	1.322578
21	1	0	4.805919	-4.46558	-0.895987
22	1	0	2.360875	-2.969776	-4.622656
23	1	0	4.982452	1.827887	-2.776199
24	1	0	6.439295	-1.117773	-3.545969

25	1	0	3.533487	1.525047	-7.084236
26	1	0	-1.047895	0.494349	-3.828086
27	1	0	-4.659742	-1.93318	0.752349
28	1	0	-4.361444	1.152395	3.823439
29	1	0	-2.059801	-1.092022	4.71011
30	1	0	1.39414	1.774445	3.840651
31	1	0	3.104187	5.742832	3.581022
32	1	0	1.553841	7.738213	1.346549
33	1	0	0.981667	8.157706	4.611366
34	1	0	-4.938582	4.746928	2.237028
35	1	0	-3.884697	7.46515	3.937564
36	1	0	-3.288755	7.212703	0.66556
37	1	0	-0.501751	-5.905602	-2.91711
38	1	0	-3.597576	-5.485051	-1.681698
39	1	0	-2.383693	-3.314204	-3.934051
40	1	0	-2.941744	-7.526237	2.218281
41	1	0	-1.131046	-7.220156	5.033467
42	1	0	-3.525479	-4.974003	4.287265
43	1	0	-2.088773	2.394051	-0.700837

10A000017_en_		Standard Orientation (A.U.)			
Center number	Atomic number	Atomic Type	X	Y	Z
0	6	0	1.953431	-2.550688	-0.572697
1	6	0	-0.977767	-2.369208	-0.978617
2	6	0	-1.878204	-4.796454	0.3309
3	6	0	0.025283	-6.455051	0.534535
4	6	0	2.471979	-5.41951	-0.471969
5	6	0	3.650184	-1.049469	-2.35745
6	6	0	6.458377	-1.578282	-1.936803
7	8	0	7.944902	0.058033	-3.403945
8	8	0	3.401326	1.599045	-1.994001
9	6	0	-2.246699	0.008052	0.142678
10	6	0	-1.605806	0.573282	2.924761
11	6	0	-2.768713	3.016589	3.79142
12	6	0	-1.617902	5.290568	3.845084
13	6	0	-3.000241	7.610292	4.74895
14	6	0	1.105713	5.723349	3.113947
15	6	0	-1.707222	-2.556966	-3.798242
16	6	0	-4.586356	-5.262806	1.004524
17	8	0	-1.629173	2.13529	-1.405792

18	1	0	2.350608	-1.831772	1.337304
19	1	0	-0.182451	-8.381128	1.251834
20	1	0	4.120742	-5.883205	0.711737
21	1	0	2.878019	-6.196367	-2.373078
22	1	0	3.214122	-1.540223	-4.345964
23	1	0	6.869755	-1.378814	0.114661
24	1	0	6.923102	-3.5321	-2.482446
25	1	0	7.113955	1.687629	-3.268007
26	1	0	1.608155	2.053397	-2.055907
27	1	0	-4.312988	-0.28841	0.007304
28	1	0	-2.2885	-1.005837	4.095532
29	1	0	0.452348	0.644888	3.154784
30	1	0	-4.758714	2.932898	4.366135
31	1	0	-4.960199	7.190746	5.297781
32	1	0	-2.027704	8.449309	6.39383
33	1	0	-3.030743	9.085871	3.274589
34	1	0	2.246257	6.152077	4.808629
35	1	0	1.979588	4.12609	2.122495
36	1	0	1.264068	7.391938	1.874075
37	1	0	-0.839059	-4.230864	-4.671579
38	1	0	-3.770712	-2.734271	-4.000454
39	1	0	-1.126526	-0.871846	-4.858724
40	1	0	-5.256666	-3.972514	2.499089
41	1	0	-5.838073	-4.966348	-0.636732
42	1	0	-4.865036	-7.208999	1.678228
43	1	0	-2.199348	3.628919	-0.503406

10A000018_en_		Standard Orientation (A.U.)			
Center number	Atomic number	Atomic Type	X	Y	Z
0	6	0	1.974042	-2.133274	-0.852161
1	6	0	-0.794148	-1.638368	-1.808997
2	6	0	-2.008809	-4.243614	-1.421161
3	6	0	-0.233536	-6.040345	-1.251458
4	6	0	2.392966	-4.972584	-1.402014
5	6	0	4.055745	-0.332788	-1.759516
6	6	0	6.712254	-1.115207	-0.900126
7	8	0	7.764176	-3.146759	-2.272558
8	8	0	3.711721	2.128463	-0.779848
9	6	0	-2.267516	0.446746	-0.389869
10	6	0	-2.245768	0.229375	2.512869

11	6	0	-3.541785	2.438756	3.741696
12	6	0	-2.401781	4.540173	4.62642
13	6	0	-3.9171	6.638789	5.813593
14	6	0	0.420011	4.961285	4.600491
15	6	0	-0.859236	-1.03674	-4.663933
16	6	0	-4.805536	-4.666616	-1.498475
17	8	0	-1.327465	2.857279	-1.156558
18	1	0	1.945619	-1.959266	1.220701
19	1	0	-0.630456	-8.063943	-1.131468
20	1	0	3.694422	-5.855789	-0.030049
21	1	0	3.211068	-5.280583	-3.301059
22	1	0	4.101549	-0.315478	-3.853938
23	1	0	7.93842	0.534254	-1.213781
24	1	0	6.693043	-1.475313	1.163476
25	1	0	7.03288	-4.698133	-1.66451
26	1	0	1.966908	2.656706	-1.080137
27	1	0	-4.255667	0.315488	-1.026418
28	1	0	-3.18337	-1.549426	3.048708
29	1	0	-0.287202	0.097814	3.176354
30	1	0	-5.609208	2.34364	3.858483
31	1	0	-5.954132	6.227409	5.822116
32	1	0	-3.30789	6.95855	7.783793
33	1	0	-3.613944	8.441836	4.809185
34	1	0	1.468321	3.610821	3.427417
35	1	0	0.857749	6.872626	3.892331
36	1	0	1.177014	4.876875	6.543935
37	1	0	-2.822297	-1.000825	-5.353706
38	1	0	-0.025211	0.816753	-5.075646
39	1	0	0.167292	-2.486806	-5.74164
40	1	0	-5.255706	-6.694527	-1.431789
41	1	0	-5.783601	-3.748134	0.097211
42	1	0	-5.645202	-3.881456	-3.238293
43	1	0	-2.057693	4.098912	-0.018565

10A000019_en_		Standard Orientation (A.U.)			
Center number	Atomic number	Atomic Type	X	Y	Z
0	6	0	1.983311	-2.676971	-0.472794
1	6	0	-0.928441	-2.433208	-0.974212
2	6	0	-1.926844	-4.819253	0.340413
3	6	0	-0.070619	-6.519037	0.625231
4	6	0	2.430231	-5.555664	-0.318515
5	6	0	3.76903	-1.238944	-2.222021
6	6	0	6.549589	-1.821156	-1.701275
7	8	0	8.119203	-0.241581	-3.143692
8	8	0	3.566261	1.419753	-1.903557
9	6	0	-2.176142	-0.011023	0.072195
10	6	0	-1.601377	0.586943	2.861637
11	6	0	-2.742603	3.064051	3.657671
12	6	0	-1.554708	5.319203	3.700747
13	6	0	-2.92006	7.677366	4.527818
14	6	0	1.192844	5.694459	3.030561
15	6	0	-1.572188	-2.64672	-3.812768
16	6	0	-4.664294	-5.21067	0.938521
17	8	0	-1.467896	2.076371	-1.491816
18	1	0	2.338336	-1.943041	1.439852
19	1	0	-0.344883	-8.429195	1.362457
20	1	0	4.029391	-6.041632	0.92298
21	1	0	2.876637	-6.368781	-2.19518
22	1	0	3.387822	-1.748976	-4.216922
23	1	0	6.897007	-1.596522	0.359348
24	1	0	6.989347	-3.793111	-2.200288
25	1	0	7.323473	1.408783	-3.057645
26	1	0	1.785852	1.911345	-2.0259
27	1	0	-4.243552	-0.26535	-0.118374
28	1	0	-2.34248	-0.961026	4.038418
29	1	0	0.45096	0.627279	3.144599
30	1	0	-4.747247	3.024873	4.184088
31	1	0	-4.900321	7.300341	5.032953
32	1	0	-1.976223	8.53063	6.182126
33	1	0	-2.887859	9.12643	3.027339
34	1	0	1.40775	7.327107	1.751583
35	1	0	2.295103	6.153914	4.742487
36	1	0	2.069613	4.059695	2.104949
37	1	0	-0.716	-4.353184	-4.633299
38	1	0	-3.631681	-2.77985	-4.078838

39	1	0	-0.917538	-0.9918	-4.877523
40	1	0	-5.859788	-4.909157	-0.743322
41	1	0	-5.010409	-7.139193	1.631639
42	1	0	-5.347239	-3.882486	2.393656
43	1	0	-2.037673	3.596504	-0.634642

10A000020_en_		Standard Orientation (A.U.)			
Center number	Atomic number	Atomic Type	X	Y	Z
0	6	0	2.386548	-2.582951	-0.348526
1	6	0	-0.523403	-2.622382	-0.904922
2	6	0	-1.405006	-4.799825	0.796236
3	6	0	0.547191	-6.280165	1.436842
4	6	0	3.012972	-5.332199	0.396432
5	6	0	4.133697	-1.439023	-2.35166
6	6	0	6.918508	-1.623864	-1.596747
7	8	0	7.383466	-0.260939	0.642059
8	8	0	3.711416	1.189821	-2.675915
9	6	0	-1.924153	-0.127526	-0.276374
10	6	0	-1.365482	0.947943	2.351896
11	6	0	-3.082523	3.093257	3.065204
12	6	0	-2.622895	5.568099	2.724815
13	6	0	-4.527954	7.536264	3.501196
14	6	0	-0.237922	6.605128	1.568189
15	6	0	-1.099225	-3.352418	-3.669408
16	6	0	-4.128216	-5.244869	1.423911
17	8	0	-1.340817	1.822637	-2.072903
18	1	0	2.679624	-1.451289	1.365439
19	1	0	0.371547	-8.027056	2.525761
20	1	0	4.580556	-5.422567	1.762542
21	1	0	3.606958	-6.471696	-1.257883
22	1	0	3.89369	-2.465749	-4.164459
23	1	0	7.4794	-3.600004	-1.275858
24	1	0	8.063448	-0.893835	-3.19198
25	1	0	6.569986	1.359683	0.366754
26	1	0	1.899458	1.552094	-2.657354
27	1	0	-3.971807	-0.511789	-0.401952
28	1	0	-1.530204	-0.601143	3.732207
29	1	0	0.618623	1.555403	2.393765
30	1	0	-4.90568	2.557124	3.894852
31	1	0	-5.104755	8.701261	1.867887

32	1	0	-6.235134	6.689126	4.331061
33	1	0	-3.710868	8.85514	4.89769
34	1	0	0.712249	7.911495	2.889469
35	1	0	1.108071	5.139696	0.992622
36	1	0	-0.696608	7.718031	-0.13531
37	1	0	-3.14385	-3.633269	-3.951531
38	1	0	-0.45872	-1.895407	-5.006835
39	1	0	-0.151628	-5.131047	-4.173923
40	1	0	-4.373694	-7.026839	2.464539
41	1	0	-4.916871	-3.712737	2.598133
42	1	0	-5.307385	-5.341878	-0.294015
43	1	0	-2.419441	1.647745	-3.531036

Table S3. Gibbs free energies[a] and equilibrium populations[b] of low-energy conformers of **10B**.

Conformers	ΔG(a.u.)	P(%)/100	G(a.u.)
10B000001.out	0.00076	20.25	-812.522658
10B000002.out	0.00449	0.39	-812.518932
10B000003.out	0.0038	0.81	-812.519624
10B000004.out	0.00293	2.04	-812.520493
10B000005.out	0.0034	1.25	-812.520026
10B000006.out	0.01311	0.0	-812.510312
10B000007.out	0.00851	0.01	-812.514908
10B000008.out	0.01319	0.0	-812.510235
10B000009.out	0.0	45.47	-812.523422
10B000010.out	0.01243	0.0	-812.510987
10B000011.out	0.00487	0.26	-812.518548
10B000012.out	0.01068	0.0	-812.512743
10B000013.out	0.00599	0.08	-812.517429
10B000014.out	0.00431	0.47	-812.519114
10B000015.out	0.00316	1.61	-812.520266
10B000016.out	0.00292	2.06	-812.5205
10B000017.out	0.00077	20.0	-812.522647
10B000018.out	0.00204	5.26	-812.521385
10B000019.out	0.00677	0.04	-812.516649
10B000020.out	0.01295	0.0	-812.510475

[a]wB97M-V/def2-TZVP, in a.u.

[b]From ΔG values at 298.15K.

Table S4. Cartesian coordinates for the low-energy reoptimized random reseach conformers of **10B** at B3LYP-D3(BJ)/6-31G* level of theory in chloroform.

10B000001_en_		Standard Orientation (A.U.)			
Center number	Atomic number	Atomic Type	X	Y	Z
0	6	0	1.421675	-2.102171	-0.649454
1	6	0	-1.259217	-1.017737	-1.308208
2	6	0	-3.013389	-3.154439	-0.445006
3	6	0	-1.71542	-5.282973	0.006741
4	6	0	1.070821	-4.994695	-0.455522
5	6	0	3.600954	-1.286433	-2.375688
6	6	0	6.154944	-2.33658	-1.505375
7	8	0	6.902474	-1.196985	0.810679
8	8	0	3.803214	1.3818	-2.52657
9	6	0	-1.728789	1.522711	0.066821
10	6	0	-1.758556	1.352681	2.982982
11	6	0	-2.347839	3.877221	4.139899
12	6	0	-4.590016	4.691179	5.031513
13	6	0	-4.891694	7.33418	6.050227
14	6	0	-6.951197	3.101908	5.135883
15	6	0	-1.598803	-0.604826	-4.171988
16	6	0	-5.830795	-2.897013	-0.375175
17	8	0	-4.067286	2.516131	-0.794738
18	1	0	1.898931	-1.413302	1.256239
19	1	0	-2.583913	-7.083928	0.528929
20	1	0	2.249866	-5.84514	1.041581
21	1	0	1.627443	-5.951095	-2.233745
22	1	0	3.244335	-1.977634	-4.307927
23	1	0	6.124166	-4.419236	-1.376673
24	1	0	7.601985	-1.802674	-2.900244
25	1	0	5.941233	-1.958236	2.162591
26	1	0	4.758586	1.880899	-1.04243
27	1	0	-0.163097	2.786052	-0.502263
28	1	0	-3.129721	-0.094311	3.56433
29	1	0	0.103854	0.721301	3.660861
30	1	0	-0.77773	5.23233	4.151477
31	1	0	-6.39752	8.360237	5.03291
32	1	0	-5.465114	7.293861	8.056657
33	1	0	-3.136084	8.435503	5.897227
34	1	0	-6.655266	1.146244	4.517545

35	1	0	-7.714355	3.063681	7.076924
36	1	0	-8.445243	3.927781	3.936158
37	1	0	-1.183048	-2.353788	-5.219671
38	1	0	-3.547949	-0.037343	-4.590583
39	1	0	-0.338397	0.899857	-4.851611
40	1	0	-6.431031	-1.295356	0.802385
41	1	0	-6.578452	-2.492532	-2.277829
42	1	0	-6.724664	-4.641681	0.318532
43	1	0	-4.41759	3.992562	0.227631

10B000002_en_		Standard Orientation (A.U.)			
Center number	Atomic number	Atomic Type	X	Y	Z
0	6	0	0.572466	-1.480081	-0.220525
1	6	0	-2.091065	-1.486803	-1.49623
2	6	0	-2.808802	-4.289006	-1.225274
3	6	0	-0.751075	-5.712362	-0.820194
4	6	0	1.622719	-4.163399	-0.642615
5	6	0	2.372598	0.669356	-0.935101
6	6	0	4.833337	0.549529	0.567763
7	8	0	4.225318	0.634653	3.179761
8	8	0	1.239025	3.074998	-0.617421
9	6	0	-3.960316	0.40367	-0.240672
10	6	0	-4.254698	0.14271	2.663718
11	6	0	-2.310445	1.540314	4.215992
12	6	0	-2.138813	4.067326	4.470149
13	6	0	-0.047326	5.248833	6.001763
14	6	0	-3.936991	5.900417	3.240914
15	6	0	-1.930232	-0.8362	-4.331509
16	6	0	-5.425808	-5.303515	-1.616426
17	8	0	-6.366383	0.3636	-1.441649
18	1	0	0.268727	-1.331305	1.822992
19	1	0	-0.76745	-7.776777	-0.726008
20	1	0	2.890021	-4.759901	0.899343
21	1	0	2.735798	-4.300626	-2.412215
22	1	0	2.875849	0.529148	-2.95296
23	1	0	5.889989	-1.191608	0.085784
24	1	0	5.998072	2.19164	0.009141
25	1	0	5.758593	0.881085	4.131907
26	1	0	0.663154	3.157472	1.123465
27	1	0	-3.220976	2.297051	-0.641226

28	1	0	-6.153207	0.878071	3.098733
29	1	0	-4.250833	-1.8726	3.193696
30	1	0	-0.878938	0.402134	5.180193
31	1	0	1.312572	3.826382	6.66408
32	1	0	0.997171	6.654376	4.865735
33	1	0	-0.810978	6.274688	7.651458
34	1	0	-5.564822	4.977703	2.344751
35	1	0	-4.648605	7.275368	4.638363
36	1	0	-2.951677	7.006428	1.770479
37	1	0	-0.577814	-2.077571	-5.309223
38	1	0	-3.788735	-1.047747	-5.230811
39	1	0	-1.333143	1.138454	-4.588716
40	1	0	-5.4233	-7.382455	-1.644719
41	1	0	-6.725494	-4.728894	-0.084679
42	1	0	-6.247843	-4.635041	-3.409546
43	1	0	-7.203821	-1.192778	-1.00055

10B000003_en_		Standard Orientation (A.U.)			
Center number	Atomic number	Atomic Type	X	Y	Z
0	6	0	0.695355	-0.754466	-0.508712
1	6	0	-1.743938	-0.315019	-2.123387
2	6	0	-2.520076	-3.014727	-2.810941
3	6	0	-0.654051	-4.656657	-2.317619
4	6	0	1.660848	-3.407857	-1.24261
5	6	0	2.711031	1.325768	-0.585318
6	6	0	4.806874	0.848145	1.355414
7	8	0	3.870513	1.205462	3.847951
8	8	0	1.67453	3.753544	-0.108601
9	6	0	-3.774055	1.147068	-0.57435
10	6	0	-4.919694	-0.262443	1.704792
11	6	0	-3.268427	-0.622287	3.999976
12	6	0	-2.578641	1.168966	5.66832
13	6	0	-0.916226	0.567228	7.899943
14	6	0	-3.345121	3.899679	5.439854
15	6	0	-1.154823	1.156263	-4.571436
16	6	0	-4.975732	-3.624175	-4.080624
17	8	0	-5.896055	1.810265	-2.093072
18	1	0	0.053487	-0.858937	1.459925
19	1	0	-0.739038	-6.675314	-2.751793
20	1	0	2.471391	-4.440303	0.378956

21	1	0	3.184115	-3.307204	-2.677549
22	1	0	3.57405	1.398205	-2.481188
23	1	0	5.656816	-1.042205	1.106149
24	1	0	6.306218	2.265935	1.10079
25	1	0	2.698984	-0.141877	4.235725
26	1	0	1.59481	3.896443	1.718827
27	1	0	-2.83242	2.873249	0.120247
28	1	0	-6.607246	0.845545	2.213977
29	1	0	-5.61729	-2.112884	1.06803
30	1	0	-2.599035	-2.551809	4.34603
31	1	0	-1.878089	1.021584	9.695249
32	1	0	-0.382465	-1.443065	7.954965
33	1	0	0.827804	1.712424	7.859392
34	1	0	-4.069208	4.616981	7.258963
35	1	0	-1.695466	5.08784	4.961589
36	1	0	-4.795042	4.220401	3.991005
37	1	0	-0.614692	3.119061	-4.149761
38	1	0	0.412928	0.259435	-5.601252
39	1	0	-2.787858	1.161067	-5.85885
40	1	0	-6.603631	-2.897494	-3.013879
41	1	0	-5.081111	-2.717757	-5.955746
42	1	0	-5.189311	-5.674834	-4.350271
43	1	0	-5.319872	2.910403	-3.426627

10B000004_en_		Standard Orientation (A.U.)			
Center number	Atomic number	Atomic Type	X	Y	Z
0	6	0	0.390981	-1.434998	0.101996
1	6	0	-2.171412	-1.490729	-1.370745
2	6	0	-2.866117	-4.304938	-1.170168
3	6	0	-0.81197	-5.686118	-0.620184
4	6	0	1.517089	-4.096626	-0.276871
5	6	0	2.196361	0.736239	-0.457884
6	6	0	4.63849	0.595362	1.089886
7	8	0	6.103908	2.77415	0.716852
8	8	0	1.064921	3.14947	0.008324
9	6	0	-4.161617	0.340973	-0.258019
10	6	0	-4.749641	0.004344	2.59414
11	6	0	-2.986134	1.33323	4.401034
12	6	0	-2.791243	3.849667	4.746414
13	6	0	-0.918105	4.954641	6.585531

14	6	0	-4.365294	5.758183	3.334116
15	6	0	-1.797632	-0.822184	-4.183274
16	6	0	-5.427002	-5.344282	-1.774914
17	8	0	-6.410439	0.032261	-1.701593
18	1	0	-0.077239	-1.321708	2.124135
19	1	0	-0.791814	-7.750321	-0.537638
20	1	0	2.703862	-4.690956	1.328547
21	1	0	2.741428	-4.181129	-1.973564
22	1	0	2.72004	0.756822	-2.472518
23	1	0	4.12832	0.33331	3.115735
24	1	0	5.773641	-1.056895	0.527498
25	1	0	4.887365	4.144039	0.611907
26	1	0	0.155254	3.031094	1.600681
27	1	0	-3.412673	2.263	-0.546324
28	1	0	-6.686087	0.725961	2.874258
29	1	0	-4.823643	-2.025418	3.041282
30	1	0	-1.757069	0.130312	5.552703
31	1	0	-1.888594	6.07487	8.053879
32	1	0	0.211143	3.485863	7.527227
33	1	0	0.391555	6.261933	5.619507
34	1	0	-5.137942	7.181598	4.645744
35	1	0	-3.199301	6.795002	1.94826
36	1	0	-5.953669	4.902095	2.308535
37	1	0	-0.298673	-1.988812	-5.02851
38	1	0	-3.553583	-1.143931	-5.23847
39	1	0	-1.279706	1.180329	-4.403761
40	1	0	-5.4264	-7.420348	-1.652352
41	1	0	-6.887084	-4.590695	-0.501524
42	1	0	-6.024471	-4.791682	-3.691436
43	1	0	-7.517854	1.432989	-1.334806

10B000005_en_		Standard Orientation (A.U.)			
Center number	Atomic number	Atomic Type	X	Y	Z
0	6	0	-0.303088	-1.525931	-0.027688
1	6	0	-1.630446	-0.126525	-2.268467
2	6	0	-3.665515	-2.076805	-2.976205
3	6	0	-3.047138	-4.376902	-2.108864
4	6	0	-0.627826	-4.35978	-0.621777
5	6	0	2.371718	-0.717905	0.652485
6	6	0	3.49975	-2.270774	2.821541

7	8	0	5.82212	-1.248716	3.588282
8	8	0	2.482267	1.888834	1.358991
9	6	0	-2.664015	2.524871	-1.520589
10	6	0	-4.412393	2.540576	0.833313
11	6	0	-3.071797	3.215594	3.247228
12	6	0	-2.856427	1.871375	5.399133
13	6	0	-1.388771	2.885085	7.620234
14	6	0	-3.99229	-0.705123	5.805991
15	6	0	0.160519	0.215287	-4.539198
16	6	0	-5.903293	-1.520598	-4.62316
17	8	0	-3.829279	3.749906	-3.607914
18	1	0	-1.466898	-1.184378	1.653841
19	1	0	-4.115194	-6.098244	-2.515522
20	1	0	-0.707151	-5.529159	1.100081
21	1	0	0.959854	-5.1034	-1.765686
22	1	0	3.643163	-0.895793	-0.986465
23	1	0	2.10904	-2.320837	4.402128
24	1	0	3.816933	-4.241687	2.229631
25	1	0	5.606052	0.568507	3.445188
26	1	0	1.0778	2.205513	2.50083
27	1	0	-1.005395	3.703262	-1.105815
28	1	0	-5.83655	4.017813	0.466343
29	1	0	-5.435822	0.736491	0.993934
30	1	0	-2.171207	5.08513	3.214887
31	1	0	-2.594394	3.008794	9.318383
32	1	0	0.188122	1.608119	8.111648
33	1	0	-0.602921	4.771935	7.24322
34	1	0	-2.503097	-2.094145	6.260597
35	1	0	-5.285432	-0.675689	7.442123
36	1	0	-5.049348	-1.409443	4.167105
37	1	0	-0.875949	0.971845	-6.169412
38	1	0	1.673313	1.568178	-4.084953
39	1	0	1.029869	-1.591773	-5.089176
40	1	0	-6.926455	-3.265757	-5.101845
41	1	0	-7.278947	-0.263322	-3.678435
42	1	0	-5.341014	-0.599669	-6.40538
43	1	0	-5.382764	2.881116	-3.995633

10B000006_en_		Standard Orientation (A.U.)			
Center number	Atomic number	Atomic Type	X	Y	Z
0	6	0	0.859332	-0.406467	-0.458005
1	6	0	-1.746149	-0.351657	-1.894568
2	6	0	-2.265472	-3.169991	-2.323851
3	6	0	-0.23621	-4.586945	-1.789709
4	6	0	1.988984	-3.055553	-0.935637
5	6	0	2.823018	1.692535	-0.880839
6	6	0	1.977028	4.360199	-0.1115
7	8	0	1.073516	4.504431	2.396704
8	8	0	3.911213	1.74568	-3.326895
9	6	0	-3.815074	0.974791	-0.253792
10	6	0	-4.739256	-0.50406	2.097725
11	6	0	-2.889665	-0.951938	4.208772
12	6	0	-2.356031	0.664021	6.096784
13	6	0	-0.408183	0.021675	8.069127
14	6	0	-3.543562	3.235544	6.356676
15	6	0	-1.625881	0.941513	-4.507628
16	6	0	-4.685204	-4.134698	-3.44426
17	8	0	-5.960262	1.6661	-1.721909
18	1	0	0.394637	-0.284691	1.556947
19	1	0	-0.155785	-6.634428	-2.062031
20	1	0	2.918807	-3.824736	0.766509
21	1	0	3.449474	-2.979784	-2.426724
22	1	0	4.423204	1.205194	0.377531
23	1	0	3.587176	5.653276	-0.441926
24	1	0	0.416978	5.013513	-1.322298
25	1	0	2.332616	3.799172	3.51614
26	1	0	2.579709	2.085999	-4.52422
27	1	0	-3.020266	2.776552	0.412627
28	1	0	-6.36308	0.59317	2.796186
29	1	0	-5.496151	-2.339872	1.471721
30	1	0	-1.886614	-2.762494	4.175174
31	1	0	0.436616	-1.851952	7.755627
32	1	0	1.131048	1.43534	8.084233
33	1	0	-1.234738	0.05391	9.985396
34	1	0	-5.236737	3.45828	5.17708
35	1	0	-4.068038	3.620307	8.337073
36	1	0	-2.188768	4.70402	5.763398
37	1	0	-3.450048	0.799927	-5.480234
38	1	0	-1.227105	2.977343	-4.354789

39	1	0	-0.191004	0.027458	-5.704588
40	1	0	-4.589572	-6.185028	-3.773224
41	1	0	-6.332856	-3.793349	-2.210811
42	1	0	-5.096839	-3.220207	-5.271482
43	1	0	-6.901673	0.149933	-2.101493

10B000007_en_		Standard Orientation (A.U.)			
Center number	Atomic number	Atomic Type	X	Y	Z
0	6	0	1.105184	-1.138033	-0.720039
1	6	0	-1.58374	-0.573016	-1.855724
2	6	0	-2.376964	-3.184395	-2.832526
3	6	0	-0.404647	-4.771199	-2.921364
4	6	0	2.011544	-3.580744	-2.038491
5	6	0	3.157627	0.905925	-0.680615
6	6	0	2.511501	3.293764	0.838645
7	8	0	2.227583	2.840321	3.438721
8	8	0	3.827351	1.560532	-3.195812
9	6	0	-3.402331	0.467552	0.21373
10	6	0	-3.976458	-1.38424	2.400742
11	6	0	-2.346828	-1.125574	4.725311
12	6	0	-2.381059	0.824461	6.364692
13	6	0	-0.65905	0.889751	8.629582
14	6	0	-4.05382	3.10644	6.047668
15	6	0	-1.528892	1.273604	-4.114144
16	6	0	-5.001302	-3.756284	-3.727524
17	8	0	-5.794671	1.162956	-0.795193
18	1	0	0.804179	-1.634951	1.275796
19	1	0	-0.507541	-6.699642	-3.657195
20	1	0	3.114556	-4.792359	-0.74799
21	1	0	3.25977	-3.13019	-3.649983
22	1	0	4.793536	0.029962	0.295857
23	1	0	4.10121	4.629812	0.638021
24	1	0	0.842088	4.233746	0.001436
25	1	0	0.783932	1.740974	3.721525
26	1	0	5.386674	2.504802	-3.144308
27	1	0	-2.499968	2.169445	1.02598
28	1	0	-5.966669	-1.042343	2.89853
29	1	0	-3.871206	-3.336996	1.698767
30	1	0	-1.052498	-2.689184	5.136725
31	1	0	0.625558	2.52917	8.521049

32	1	0	-1.757459	1.09424	10.391115
33	1	0	0.499748	-0.829214	8.774344
34	1	0	-2.880519	4.794436	5.689775
35	1	0	-5.411975	2.926279	4.489373
36	1	0	-5.113639	3.481662	7.803564
37	1	0	-0.088874	0.705102	-5.48908
38	1	0	-3.365462	1.294859	-5.094431
39	1	0	-1.071875	3.214776	-3.525426
40	1	0	-6.417765	-3.405103	-2.247234
41	1	0	-5.538024	-2.533561	-5.328929
42	1	0	-5.152149	-5.731931	-4.35834
43	1	0	-5.519684	2.304073	-2.190905

10B000008_en_		Standard Orientation (A.U.)			
Center number	Atomic number	Atomic Type	X	Y	Z
0	6	0	2.054975	-0.804602	-0.454188
1	6	0	-0.574441	0.535769	-0.908569
2	6	0	-2.175481	-1.59347	-2.046241
3	6	0	-1.078472	-3.856398	-1.711801
4	6	0	1.391683	-3.674058	-0.327151
5	6	0	4.20897	-0.467868	-2.408749
6	6	0	5.875595	1.907752	-2.304642
7	8	0	4.875476	4.043177	-3.513908
8	8	0	3.406934	-0.731005	-4.95767
9	6	0	-1.665119	1.410167	1.692896
10	6	0	-1.880147	-0.6517	3.747125
11	6	0	-3.18868	0.326666	6.071276
12	6	0	-5.654601	0.078639	6.661099
13	6	0	-6.71654	1.213335	9.047663
14	6	0	-7.542812	-1.351111	5.077498
15	6	0	-0.5568	2.877163	-2.624941
16	6	0	-4.674028	-1.119387	-3.281377
17	8	0	-4.074716	2.493618	1.26112
18	1	0	2.862672	-0.167462	1.355553
19	1	0	-1.915672	-5.652759	-2.297584
20	1	0	1.201962	-4.355541	1.635488
21	1	0	2.887991	-4.850237	-1.180616
22	1	0	5.541781	-2.018341	-1.93778
23	1	0	6.248019	2.400109	-0.31474
24	1	0	7.717247	1.382087	-3.158424

25	1	0	4.128451	3.42315	-5.066841
26	1	0	1.927936	-1.805879	-4.996976
27	1	0	-0.345368	2.883847	2.396764
28	1	0	-2.892373	-2.279225	2.949044
29	1	0	0.024	-1.296316	4.272264
30	1	0	-2.008337	1.426352	7.373806
31	1	0	-5.275023	2.246055	10.131158
32	1	0	-8.280842	2.526352	8.62032
33	1	0	-7.517767	-0.270524	10.277381
34	1	0	-6.72716	-2.152836	3.349481
35	1	0	-8.393302	-2.899524	6.187661
36	1	0	-9.116706	-0.102979	4.51475
37	1	0	-0.023516	2.368038	-4.561789
38	1	0	-2.446215	3.728898	-2.646161
39	1	0	0.80072	4.289172	-1.951212
40	1	0	-5.946305	-0.141261	-1.959671
41	1	0	-4.470049	0.133564	-4.933192
42	1	0	-5.554772	-2.893615	-3.912844
43	1	0	-4.827578	2.775356	2.906372

10B000009_en_		Standard Orientation (A.U.)			
Center number	Atomic number	Atomic Type	X	Y	Z
0	6	0	1.21369	-2.26303	-0.506962
1	6	0	-1.09857	-0.61888	-1.371945
2	6	0	-3.270511	-2.531819	-1.287178
3	6	0	-2.396721	-4.902157	-1.097453
4	6	0	0.439214	-5.030052	-1.022056
5	6	0	3.774741	-1.54401	-1.605275
6	6	0	5.924513	-3.186311	-0.584986
7	8	0	8.297272	-2.185449	-1.352122
8	8	0	4.317928	1.007417	-1.018702
9	6	0	-1.468523	1.679014	0.403277
10	6	0	-2.102001	0.999535	3.171416
11	6	0	-2.5351	3.343383	4.712703
12	6	0	-4.766562	4.381391	5.366202
13	6	0	-4.877995	6.811251	6.84859
14	6	0	-7.306753	3.256714	4.737099
15	6	0	-0.794872	0.351816	-4.102966
16	6	0	-5.98279	-1.799008	-1.626533
17	8	0	-3.426837	3.208704	-0.615321

18	1	0	1.393698	-2.045031	1.552905
19	1	0	-3.593471	-6.586684	-1.122554
20	1	0	1.172447	-6.342569	0.422524
21	1	0	1.18969	-5.711189	-2.855794
22	1	0	3.720134	-1.806147	-3.690263
23	1	0	5.914811	-3.118343	1.495932
24	1	0	5.69927	-5.177547	-1.167737
25	1	0	8.516546	-2.543146	-3.130558
26	1	0	6.137579	1.169886	-1.192457
27	1	0	0.33938	2.725841	0.389301
28	1	0	-3.756337	-0.255782	3.206591
29	1	0	-0.518883	-0.064888	3.998806
30	1	0	-0.817216	4.364037	5.267476
31	1	0	-2.985631	7.580963	7.230088
32	1	0	-5.979324	8.260851	5.828044
33	1	0	-5.845259	6.522308	8.675549
34	1	0	-8.392094	4.539804	3.50034
35	1	0	-7.178467	1.421161	3.783874
36	1	0	-8.442949	3.013956	6.469653
37	1	0	-0.443188	-1.230336	-5.407752
38	1	0	-2.512844	1.338125	-4.712567
39	1	0	0.789803	1.6891	-4.230331
40	1	0	-7.229253	-3.457182	-1.479712
41	1	0	-6.57465	-0.364638	-0.24652
42	1	0	-6.280454	-0.923451	-3.494102
43	1	0	-3.760897	4.51657	0.61983

10B000010_en_		Standard Orientation (A.U.)			
Center number	Atomic number	Atomic Type	X	Y	Z
0	6	0	0.904682	-0.396032	-0.464364
1	6	0	-1.718874	-0.374975	-1.86814
2	6	0	-2.208727	-3.194654	-2.315332
3	6	0	-0.162191	-4.588414	-1.783503
4	6	0	2.056568	-3.033817	-0.950445
5	6	0	2.836058	1.727368	-0.910035
6	6	0	1.971546	4.379431	-0.106365
7	8	0	1.13355	4.501031	2.427703
8	8	0	3.877569	1.812854	-3.374597
9	6	0	-3.770036	0.876122	-0.172621
10	6	0	-4.631324	-0.682919	2.151085

11	6	0	-2.746076	-1.100221	4.236016
12	6	0	-2.213028	0.515257	6.124538
13	6	0	-0.217747	-0.095012	8.059151
14	6	0	-3.442255	3.064622	6.415923
15	6	0	-1.669029	0.949504	-4.468773
16	6	0	-4.623258	-4.155532	-3.444476
17	8	0	-5.917914	1.354121	-1.722767
18	1	0	0.467016	-0.27731	1.556545
19	1	0	-0.061533	-6.635694	-2.050674
20	1	0	3.009939	-3.788205	0.74589
21	1	0	3.504815	-2.942976	-2.452912
22	1	0	4.46465	1.252286	0.316682
23	1	0	3.553244	5.697709	-0.471965
24	1	0	0.370547	5.01391	-1.27267
25	1	0	2.435209	3.811264	3.507549
26	1	0	2.512359	2.097825	-4.548216
27	1	0	-2.984256	2.679541	0.532372
28	1	0	-6.284023	0.333969	2.921511
29	1	0	-5.34044	-2.516153	1.480096
30	1	0	-1.708095	-2.889747	4.171376
31	1	0	1.294377	1.347486	8.04632
32	1	0	-1.008645	-0.08305	9.990625
33	1	0	0.655538	-1.951412	7.723178
34	1	0	-5.182646	3.245977	5.297913
35	1	0	-3.908145	3.448818	8.410844
36	1	0	-2.138476	4.556373	5.768654
37	1	0	-3.506527	0.768935	-5.407255
38	1	0	-1.297737	2.990582	-4.306899
39	1	0	-0.2371	0.068274	-5.694351
40	1	0	-4.681672	-6.233855	-3.422088
41	1	0	-6.296263	-3.40875	-2.465119
42	1	0	-4.806702	-3.535611	-5.427482
43	1	0	-7.202549	2.126053	-0.683128

10B000011_en_		Standard Orientation (A.U.)			
Center number	Atomic number	Atomic Type	X	Y	Z
0	6	0	0.516419	-1.04424	-0.293894
1	6	0	-2.10024	-1.445489	-1.618471
2	6	0	-2.673042	-4.207997	-0.963828
3	6	0	-0.605258	-5.403207	-0.119508

4	6	0	1.670983	-3.708782	-0.015275
5	6	0	2.285044	0.91691	-1.483936
6	6	0	4.665815	1.335534	0.110292
7	8	0	4.058106	2.584497	2.409781
8	8	0	1.07317	3.284567	-1.816197
9	6	0	-4.109973	0.474756	-0.654458
10	6	0	-4.87459	0.269641	2.164402
11	6	0	-2.972558	1.058086	4.135802
12	6	0	-2.25964	3.43707	4.682065
13	6	0	-0.312515	3.98369	6.684825
14	6	0	-3.275825	5.725957	3.331551
15	6	0	-1.885168	-1.190102	-4.512957
16	6	0	-5.176893	-5.438738	-1.46389
17	8	0	-6.338181	0.411915	-2.158511
18	1	0	0.093407	-0.358838	1.61749
19	1	0	-0.53018	-7.418539	0.332272
20	1	0	2.775883	-3.926214	1.740564
21	1	0	2.990225	-4.149328	-1.58164
22	1	0	2.874462	0.269872	-3.37497
23	1	0	5.682888	-0.456669	0.445074
24	1	0	5.944699	2.616738	-0.912169
25	1	0	3.118779	1.434525	3.473152
26	1	0	1.188883	4.124864	-0.190471
27	1	0	-3.299632	2.356281	-0.984481
28	1	0	-6.579437	1.454707	2.329373
29	1	0	-5.47976	-1.682459	2.562935
30	1	0	-2.109724	-0.470015	5.235923
31	1	0	-1.082386	5.246171	8.157156
32	1	0	0.352568	2.247054	7.618395
33	1	0	1.338143	4.960837	5.864589
34	1	0	-4.863628	5.292099	2.069286
35	1	0	-3.90102	7.169534	4.700354
36	1	0	-1.785069	6.626801	2.179691
37	1	0	-0.375878	-2.419607	-5.246672
38	1	0	-3.666435	-1.718955	-5.433152
39	1	0	-1.468856	0.774143	-5.045232
40	1	0	-5.766091	-5.182173	-3.446303
41	1	0	-5.09737	-7.481118	-1.084688
42	1	0	-6.697169	-4.658307	-0.26544
43	1	0	-7.221241	-1.144781	-1.810101

10B000012_en_		Standard Orientation (A.U.)			
Center number	Atomic number	Atomic Type	X	Y	Z
0	6	0	0.416214	-1.196361	-0.065199
1	6	0	-1.727701	-0.010554	-1.741292
2	6	0	-3.390908	-2.346184	-2.220494
3	6	0	-2.122151	-4.492431	-1.770738
4	6	0	0.51691	-4.005246	-0.860192
5	6	0	3.065251	-0.030396	0.185248
6	6	0	3.239527	2.565764	1.441366
7	8	0	2.489081	4.546322	-0.17236
8	8	0	4.415364	0.223284	-2.126227
9	6	0	-3.134857	2.192695	-0.3818
10	6	0	-4.463877	1.444985	2.119721
11	6	0	-2.909662	1.79393	4.467383
12	6	0	-2.232802	0.055894	6.194176
13	6	0	-0.661045	0.777231	8.456455
14	6	0	-2.89531	-2.708378	6.039247
15	6	0	-0.787354	0.946658	-4.330092
16	6	0	-6.012128	-2.213941	-3.288613
17	8	0	-4.882104	3.394509	-2.040853
18	1	0	-0.322669	-1.209506	1.870494
19	1	0	-2.869551	-6.385131	-2.128251
20	1	0	1.112315	-5.234602	0.714494
21	1	0	1.894927	-4.354732	-2.404202
22	1	0	4.098903	-1.34366	1.457092
23	1	0	2.024114	2.610402	3.126164
24	1	0	5.217574	2.83954	2.071986
25	1	0	3.328524	4.222037	-1.768192
26	1	0	4.064672	-1.220234	-3.18156
27	1	0	-1.713158	3.654827	0.006633
28	1	0	-6.106561	2.721784	2.260579
29	1	0	-5.214172	-0.490431	1.992709
30	1	0	-2.278972	3.743173	4.788039
31	1	0	1.148209	-0.265578	8.468044
32	1	0	-0.236204	2.811304	8.492609
33	1	0	-1.632045	0.283168	10.236696
34	1	0	-1.16564	-3.874056	6.122531
35	1	0	-4.062881	-3.281154	7.671302
36	1	0	-3.917459	-3.215052	4.306932
37	1	0	0.309739	-0.513492	-5.323455
38	1	0	-2.397021	1.474999	-5.526228

39	1	0	0.395053	2.631657	-4.104619
40	1	0	-6.068255	-1.105363	-5.051879
41	1	0	-6.741776	-4.114418	-3.710052
42	1	0	-7.357652	-1.331877	-1.958312
43	1	0	-6.287397	2.266169	-2.308204

10B000013_en_		Standard Orientation (A.U.)			
Center number	Atomic number	Atomic Type	X	Y	Z
0	6	0	0.60081	-0.600974	-0.295901
1	6	0	-1.781114	-0.346649	-2.042185
2	6	0	-2.591096	-3.09208	-2.41607
3	6	0	-0.832196	-4.699783	-1.556235
4	6	0	1.456002	-3.384568	-0.503627
5	6	0	2.71363	1.335836	-0.704084
6	6	0	4.78671	1.108258	1.294243
7	8	0	3.696586	1.864757	3.636232
8	8	0	1.79867	3.859949	-0.67566
9	6	0	-3.826763	1.316771	-0.717453
10	6	0	-5.251118	0.111082	1.522995
11	6	0	-3.733394	-0.879031	3.716757
12	6	0	-2.681518	0.479713	5.587232
13	6	0	-1.090861	-0.761505	7.592827
14	6	0	-2.79722	3.31789	5.721
15	6	0	-1.122477	0.804643	-4.6378
16	6	0	-4.962107	-3.793368	-3.797864
17	8	0	-5.78814	2.053554	-2.406455
18	1	0	-0.049435	-0.319352	1.646144
19	1	0	-0.971829	-6.757158	-1.700332
20	1	0	2.066436	-4.154595	1.336473
21	1	0	3.090587	-3.610887	-1.79592
22	1	0	3.595911	1.050283	-2.569895
23	1	0	5.521778	-0.843812	1.386485
24	1	0	6.357271	2.381079	0.769716
25	1	0	5.021242	2.155146	4.852342
26	1	0	1.54353	4.268111	1.092169
27	1	0	-2.817228	3.011645	-0.032964
28	1	0	-6.588485	1.574248	2.160749
29	1	0	-6.420399	-1.428814	0.76429
30	1	0	-3.42404	-2.925591	3.739658
31	1	0	-1.691227	-0.195624	9.509244

32	1	0	-1.167985	-2.835271	7.467706
33	1	0	0.900386	-0.176289	7.373031
34	1	0	-3.312628	3.96378	7.635654
35	1	0	-0.907736	4.110275	5.314426
36	1	0	-4.142835	4.140946	4.371946
37	1	0	-0.521988	2.785812	-4.448701
38	1	0	0.429183	-0.255232	-5.52894
39	1	0	-2.744846	0.707028	-5.932652
40	1	0	-6.606273	-2.722717	-3.11631
41	1	0	-4.787226	-3.33446	-5.825784
42	1	0	-5.350971	-5.830185	-3.644019
43	1	0	-5.062939	3.130466	-3.684745

10B000014_en_		Standard Orientation (A.U.)			
Center number	Atomic number	Atomic Type	X	Y	Z
0	6	0	0.65993	-0.73218	-0.662204
1	6	0	-1.880533	-0.312124	-2.109772
2	6	0	-2.285444	-2.94808	-3.26203
3	6	0	-0.113684	-4.2539	-3.313077
4	6	0	2.043316	-2.819545	-2.149091
5	6	0	2.245597	1.615982	-0.07924
6	6	0	4.601657	0.965567	1.454651
7	8	0	3.816682	-0.24458	3.716583
8	8	0	0.825912	3.492354	1.200694
9	6	0	-4.047285	0.622682	-0.357001
10	6	0	-4.545647	-1.020872	2.017251
11	6	0	-2.894055	-0.447043	4.272006
12	6	0	-2.992026	1.659809	5.69687
13	6	0	-1.173543	2.063112	7.852625
14	6	0	-4.843805	3.776427	5.252293
15	6	0	-1.585737	1.614162	-4.27642
16	6	0	-4.728272	-3.831167	-4.39507
17	8	0	-6.329398	1.013137	-1.726221
18	1	0	0.183572	-1.586429	1.163901
19	1	0	0.102142	-6.11268	-4.190288
20	1	0	3.251026	-3.988649	-0.918602
21	1	0	3.294524	-2.028096	-3.6314
22	1	0	2.889932	2.492184	-1.857121
23	1	0	5.876959	-0.258241	0.334296
24	1	0	5.606957	2.751337	1.862051

25	1	0	5.247231	-0.38447	4.835618
26	1	0	0.165636	2.702101	2.719516
27	1	0	-3.499503	2.516713	0.278895
28	1	0	-6.540004	-0.687101	2.514317
29	1	0	-4.376003	-3.038039	1.523312
30	1	0	-1.454303	-1.846858	4.765043
31	1	0	-2.181453	2.161386	9.677998
32	1	0	0.247792	0.552375	7.95048
33	1	0	-0.165398	3.878565	7.638917
34	1	0	-5.799602	4.297033	7.031461
35	1	0	-3.847526	5.487523	4.591895
36	1	0	-6.294086	3.313671	3.842665
37	1	0	-1.21794	3.51394	-3.516153
38	1	0	-0.021189	1.084809	-5.540645
39	1	0	-3.330348	1.718619	-5.395455
40	1	0	-4.478524	-5.644555	-5.381228
41	1	0	-6.199725	-4.139534	-2.943917
42	1	0	-5.486274	-2.455399	-5.763073
43	1	0	-7.016307	-0.613543	-2.174376

10B000015_en_		Standard Orientation (A.U.)			
Center number	Atomic number	Atomic Type	X	Y	Z
0	6	0	1.186871	-1.902769	-0.939372
1	6	0	-1.58898	-0.948454	-1.363957
2	6	0	-3.144691	-3.287468	-0.665813
3	6	0	-1.693166	-5.357472	-0.523373
4	6	0	1.042877	-4.817407	-1.053044
5	6	0	3.209581	-0.758215	-2.669554
6	6	0	5.858739	-1.701116	-2.022194
7	8	0	6.422484	-0.780277	0.436617
8	8	0	3.216726	1.920884	-2.575423
9	6	0	-2.16007	1.41628	0.295933
10	6	0	-2.111412	0.916719	3.169059
11	6	0	-2.123565	3.322324	4.675627
12	6	0	-4.145462	4.574024	5.563856
13	6	0	-3.861105	7.058146	6.925143
14	6	0	-6.835001	3.700777	5.234135
15	6	0	-2.095984	-0.273589	-4.151813
16	6	0	-5.967477	-3.25227	-0.416116
17	8	0	-4.457237	2.620028	-0.400512

18	1	0	1.745997	-1.400047	0.997244
19	1	0	-2.415015	-7.262161	-0.172158
20	1	0	2.340401	-5.725972	0.301959
21	1	0	1.576215	-5.545101	-2.944061
22	1	0	2.814617	-1.280511	-4.645967
23	1	0	5.950719	-3.786766	-2.107177
24	1	0	7.192044	-0.930569	-3.434742
25	1	0	8.196931	-1.017319	0.773219
26	1	0	4.063442	2.353762	-1.008919
27	1	0	-0.679597	2.800213	-0.16503
28	1	0	-3.729802	-0.294716	3.667063
29	1	0	-0.4168	-0.18481	3.653168
30	1	0	-0.263817	4.189215	4.973587
31	1	0	-1.871779	7.642999	7.070095
32	1	0	-4.909187	8.569209	5.936272
33	1	0	-4.655735	6.958342	8.853369
34	1	0	-7.796214	4.860406	3.790558
35	1	0	-6.980794	1.715044	4.646103
36	1	0	-7.912928	3.921856	7.005236
37	1	0	-1.612256	-1.870625	-5.394711
38	1	0	-4.094243	0.198404	-4.441729
39	1	0	-0.97906	1.383442	-4.717252
40	1	0	-6.724044	-5.173044	-0.170313
41	1	0	-6.601067	-2.137619	1.230304
42	1	0	-6.871469	-2.422497	-2.100654
43	1	0	-5.842026	1.55923	0.127866

10B000016_en_		Standard Orientation (A.U.)			
Center number	Atomic number	Atomic Type	X	Y	Z
0	6	0	0.135127	-1.606231	0.186363
1	6	0	-2.150257	-1.114188	-1.623352
2	6	0	-3.393213	-3.739369	-1.676457
3	6	0	-1.746152	-5.51433	-0.923991
4	6	0	0.769627	-4.427797	-0.17637
5	6	0	2.385562	0.178065	-0.006317
6	6	0	4.516491	-0.489687	1.835047
7	8	0	6.4146	1.361739	1.771501
8	8	0	1.684906	2.753042	0.444309
9	6	0	-3.891369	1.043276	-0.692607
10	6	0	-4.957186	0.747228	2.022137

11	6	0	-3.255771	1.617812	4.140042
12	6	0	-2.612605	4.025445	4.669415
13	6	0	-0.857981	4.652925	6.823402
14	6	0	-3.535649	6.265425	3.171197
15	6	0	-1.251881	-0.452106	-4.316223
16	6	0	-5.989024	-4.235082	-2.687
17	8	0	-5.916137	1.233882	-2.453613
18	1	0	-0.588073	-1.451552	2.129355
19	1	0	-2.13505	-7.543165	-0.952804
20	1	0	1.571406	-5.286987	1.543568
21	1	0	2.189348	-4.710906	-1.689185
22	1	0	3.179922	0.156258	-1.930329
23	1	0	3.692689	-0.699448	3.76108
24	1	0	5.374678	-2.319083	1.334521
25	1	0	5.51362	2.950174	1.591344
26	1	0	0.555504	2.769055	1.893693
27	1	0	-2.749722	2.784417	-0.758132
28	1	0	-6.724887	1.852824	2.068793
29	1	0	-5.511014	-1.234086	2.327024
30	1	0	-2.480819	0.147391	5.373198
31	1	0	0.814438	5.700038	6.142455
32	1	0	-1.799469	5.891051	8.213882
33	1	0	-0.196386	2.948759	7.811901
34	1	0	-4.202141	7.773935	4.445471
35	1	0	-1.984931	7.082108	2.038448
36	1	0	-5.083352	5.790015	1.873074
37	1	0	0.108567	-1.86221	-5.011427
38	1	0	-2.86435	-0.396181	-5.618683
39	1	0	-0.339992	1.417089	-4.349106
40	1	0	-6.177607	-3.534774	-4.638818
41	1	0	-6.408129	-6.272054	-2.670329
42	1	0	-7.447562	-3.233221	-1.596229
43	1	0	-6.776607	2.814486	-2.163336

10B000017_en_		Standard Orientation (A.U.)			
Center number	Atomic number	Atomic Type	X	Y	Z
0	6	0	0.956368	-2.251877	-0.109219
1	6	0	-1.384449	-0.889793	-1.317744
2	6	0	-3.377453	-2.981937	-1.342352
3	6	0	-2.344037	-5.243886	-0.858915

4	6	0	0.463448	-5.11159	-0.443248
5	6	0	3.545829	-1.398783	-1.032539
6	6	0	5.709581	-2.708286	0.367631
7	8	0	8.05406	-1.561534	-0.270543
8	8	0	3.799903	1.24216	-0.689691
9	6	0	-2.141772	1.457869	0.292732
10	6	0	-3.19736	0.825825	2.922924
11	6	0	-3.218387	3.082495	4.64932
12	6	0	-5.116519	4.731583	5.005503
13	6	0	-4.820559	6.953695	6.762534
14	6	0	-7.649672	4.557428	3.722468
15	6	0	-0.869474	-0.036177	-4.055188
16	6	0	-6.077614	-2.50981	-2.048333
17	8	0	-4.026428	2.938998	-0.935978
18	1	0	0.914985	-1.841606	1.929771
19	1	0	-3.389539	-7.026344	-0.874548
20	1	0	1.110278	-6.225194	1.196571
21	1	0	1.487804	-5.878874	-2.102005
22	1	0	3.736758	-1.877464	-3.071132
23	1	0	5.470666	-2.429806	2.417024
24	1	0	5.721087	-4.760759	-0.009358
25	1	0	8.49848	-2.07871	-1.965795
26	1	0	5.607653	1.559616	-0.669691
27	1	0	-0.39986	2.57856	0.530359
28	1	0	-5.105813	0.040788	2.685144
29	1	0	-2.054998	-0.681626	3.783942
30	1	0	-1.442292	3.443196	5.657583
31	1	0	-5.114047	8.75321	5.74499
32	1	0	-6.251363	6.903126	8.282364
33	1	0	-2.938946	7.007964	7.644723
34	1	0	-9.190488	4.514418	5.130066
35	1	0	-7.96948	6.246344	2.540245
36	1	0	-7.818785	2.908394	2.482276
37	1	0	-0.211612	-1.632629	-5.214686
38	1	0	-2.604564	0.696455	-4.928693
39	1	0	0.584681	1.449048	-4.106105
40	1	0	-6.216708	-1.792555	-4.00072
41	1	0	-7.199581	-4.255848	-1.923237
42	1	0	-6.952699	-1.053787	-0.852163
43	1	0	-3.20558	4.040671	-2.130523

10B000018_en_		Standard Orientation (A.U.)			
Center number	Atomic number	Atomic Type	X	Y	Z
0	6	0	-0.246502	-1.415732	0.073518
1	6	0	-1.552658	-0.131234	-2.246763
2	6	0	-3.511827	-2.153269	-2.959253
3	6	0	-2.85593	-4.403245	-1.989814
4	6	0	-0.480871	-4.274729	-0.435474
5	6	0	2.383185	-0.508072	0.803667
6	6	0	3.485613	-1.947331	3.061268
7	8	0	5.761958	-0.84024	3.848894
8	8	0	2.399935	2.125762	1.414539
9	6	0	-2.689373	2.50246	-1.604206
10	6	0	-4.497467	2.529009	0.68146
11	6	0	-3.25353	3.37832	3.091505
12	6	0	-3.044462	2.148195	5.311251
13	6	0	-1.692204	3.331411	7.521692
14	6	0	-4.081398	-0.453504	5.811647
15	6	0	0.312719	0.182593	-4.465336
16	6	0	-5.705697	-1.691252	-4.684913
17	8	0	-4.060479	3.520567	-3.683645
18	1	0	-1.470133	-1.051317	1.706982
19	1	0	-3.870495	-6.164549	-2.359264
20	1	0	-0.579275	-5.384468	1.324672
21	1	0	1.161815	-5.014209	-1.503156
22	1	0	3.712779	-0.709834	-0.786219
23	1	0	2.052581	-1.969612	4.603469
24	1	0	3.86524	-3.931001	2.555741
25	1	0	5.501284	0.965478	3.64888
26	1	0	0.948026	2.444416	2.496239
27	1	0	-1.075065	3.752278	-1.171668
28	1	0	-5.987253	3.900117	0.192397
29	1	0	-5.431878	0.684763	0.889265
30	1	0	-2.433363	5.282506	2.995133
31	1	0	-0.076275	2.152602	8.120282
32	1	0	-0.979608	5.232463	7.076278
33	1	0	-2.953736	3.48062	9.176612
34	1	0	-5.055057	-1.282347	4.179435
35	1	0	-2.549991	-1.75119	6.381154
36	1	0	-5.427354	-0.401314	7.404024
37	1	0	-0.675776	0.711138	-6.21962
38	1	0	1.734198	1.644733	-4.048995

39	1	0	1.307683	-1.59847	-4.862252
40	1	0	-6.761202	-3.452129	-5.017195
41	1	0	-6.999684	-0.255404	-3.921407
42	1	0	-5.078495	-0.952662	-6.529843
43	1	0	-2.912903	3.713425	-5.087279

10B000019_en_		Standard Orientation (A.U.)			
Center number	Atomic number	Atomic Type	X	Y	Z
0	6	0	1.677493	-1.347881	-1.570873
1	6	0	-0.983478	-0.031201	-1.572375
2	6	0	-2.742643	-2.290531	-2.071221
3	6	0	-1.438274	-4.272219	-2.962323
4	6	0	1.341554	-3.758064	-3.174254
5	6	0	4.069055	0.134741	-2.285716
6	6	0	4.632845	2.42439	-0.594651
7	8	0	4.588682	1.855824	2.016876
8	8	0	4.218867	0.847862	-4.862299
9	6	0	-1.533855	1.355536	0.94783
10	6	0	-1.44596	-0.327314	3.327587
11	6	0	-2.213262	1.151784	5.625439
12	6	0	-4.478477	1.156036	6.789212
13	6	0	-4.971507	2.831683	9.039316
14	6	0	-6.679286	-0.48134	6.020883
15	6	0	-1.33926	1.874051	-3.757214
16	6	0	-5.56108	-2.161242	-1.837139
17	8	0	-3.938888	2.524691	0.724712
18	1	0	2.001898	-1.983698	0.377407
19	1	0	-2.310326	-6.041315	-3.579139
20	1	0	2.539159	-5.32117	-2.487025
21	1	0	1.902794	-3.414299	-5.156849
22	1	0	5.653594	-1.207112	-2.030532
23	1	0	6.46337	3.237449	-1.19364
24	1	0	3.194618	3.89767	-0.891036
25	1	0	5.934842	0.673925	2.370112
26	1	0	2.791001	1.919491	-5.232179
27	1	0	-0.074121	2.82974	1.197051
28	1	0	-2.648304	-1.996242	3.038782
29	1	0	0.505033	-0.984194	3.592354
30	1	0	-0.763134	2.442685	6.353643
31	1	0	-3.331483	4.021592	9.501199

32	1	0	-6.609765	4.07877	8.698147
33	1	0	-5.445427	1.692223	10.722627
34	1	0	-8.297927	0.692409	5.424336
35	1	0	-6.231172	-1.777458	4.467152
36	1	0	-7.34337	-1.613412	7.642287
37	1	0	-0.149474	3.566225	-3.505191
38	1	0	-0.916978	0.989298	-5.591174
39	1	0	-3.298181	2.550318	-3.802384
40	1	0	-6.162463	-1.678479	0.091463
41	1	0	-6.348002	-0.661996	-3.050626
42	1	0	-6.426783	-3.96988	-2.389523
43	1	0	-4.381632	3.126741	2.395984

10B000020_en_		Standard Orientation (A.U.)			
Center number	Atomic number	Atomic Type	X	Y	Z
0	6	0	0.808047	-0.384295	-0.422161
1	6	0	-1.812021	-0.367559	-1.831908
2	6	0	-2.261403	-3.187414	-2.315262
3	6	0	-0.210451	-4.57841	-1.797135
4	6	0	1.98234	-3.016538	-0.91476
5	6	0	2.717866	1.765012	-0.821587
6	6	0	1.806463	4.387014	-0.009851
7	8	0	1.048887	4.466167	2.54788
8	8	0	3.628229	2.0417	-3.318969
9	6	0	-3.892178	0.839991	-0.10943
10	6	0	-4.724525	-0.742944	2.210067
11	6	0	-2.824475	-1.170566	4.280207
12	6	0	-2.296677	0.438519	6.175757
13	6	0	-0.282214	-0.162135	8.092779
14	6	0	-3.548142	2.97389	6.48444
15	6	0	-1.770581	1.004713	-4.40244
16	6	0	-4.66158	-4.188296	-3.443578
17	8	0	-6.08366	1.520923	-1.507642
18	1	0	0.374457	-0.299323	1.601868
19	1	0	-0.086364	-6.619267	-2.098205
20	1	0	2.922596	-3.779379	0.784032
21	1	0	3.465125	-2.970408	-2.398242
22	1	0	4.329888	1.300472	0.457489
23	1	0	3.338959	5.748314	-0.420823
24	1	0	0.160313	4.965282	-1.133796

25	1	0	2.432015	3.881337	3.587152
26	1	0	4.18642	0.409849	-3.911523
27	1	0	-3.136244	2.641586	0.600997
28	1	0	-6.379706	0.268092	2.963395
29	1	0	-5.418739	-2.58907	1.543668
30	1	0	-1.771393	-2.95148	4.203615
31	1	0	0.595759	-2.016028	7.753272
32	1	0	1.223649	1.286524	8.060438
33	1	0	-1.053042	-0.148064	10.032216
34	1	0	-2.250675	4.479373	5.856858
35	1	0	-5.282818	3.152694	5.358691
36	1	0	-4.022628	3.336266	8.481597
37	1	0	-3.596047	0.794299	-5.362638
38	1	0	-1.43104	3.039545	-4.190844
39	1	0	-0.274103	0.242968	-5.619601
40	1	0	-6.311107	-3.889075	-2.202277
41	1	0	-5.089025	-3.255645	-5.257006
42	1	0	-4.529723	-6.232755	-3.795728
43	1	0	-6.958971	-0.008772	-1.978538
